# Exploring CO_2_ activation mechanisms with triphenylphosphine derivatives: insights from energy decomposition and deformation density analyses†

**DOI:** 10.1039/d5ra00804b

**Published:** 2025-04-22

**Authors:** Hossein Sabet-Sarvestani, Shadi Bolourian, Fereshteh Hosseini, Mohammad Javad Seddighi, Hamed Hosseini, Hossein Eshghi

**Affiliations:** a Department of Food Additives, Food Science and Technology Research Institute, Research Center for Iranian Academic Center for Education, Culture and Research (ACECR) Khorasan Razavi Branch Mashhad Iran bozorgmehr1388@gmail.com +98 9371411532 +98 9371411532; b Department of Chemistry, Faculty of Science, Ferdowsi University of Mashhad Mashhad Iran

## Abstract

This study focuses on the reaction mechanisms involving triphenylphosphine (PPh_3_) derivatives, benzyne, and CO_2_, giving mechanistic insights into two competing pathways: Path a, which involves direct C–P bond formation, and Path b, which progresses *via* a [2 + 2] cycloaddition. Comprehensive computational analysis by energy decomposition analysis (EDA) and deformation density insights was employed to elucidate the electronic and steric factors influencing the reactivity and selectivity of PPh_3_ derivatives. The results reveal that Path b is energetically and kinetically favored. In Path a, substantial repulsive interactions (Δ*E*_rep_), especially for electron-withdrawing substituents, hinder C–P bond formation, making this pathway unfavorable, while Path b benefits from compensatory effects between interaction energies, with electron-releasing *para*-substituents, such as NHMe and OMe, increasing stabilization by enhancing Δ*E*_orb_ contributions. Substituents in *meta* positions show greater distortion energies (Δ*E*_dist_), which limit their stabilizing effects compared to *para*-substituents. The deformation density analysis of transition states (TS1(b) and TS2(b)) emphasizes the crucial role of Pauli deformation (Δ*ρ*^Pauli^) and orbital deformation (Δ*ρ*^Orb^) in modulating stability. *Para*-substituents exhibit stronger electronic effects, reducing Δ*E*_int_ more effectively than *meta*-substituents, which increase Δ*E*_dist_. This positional dependence underscores the importance of substituent design in optimizing reactivity.

## Abstract 2

Developing mechanistic study in CO_2_ transformations *via* energy decomposition and deformation density analyses.

## Graphical abstract

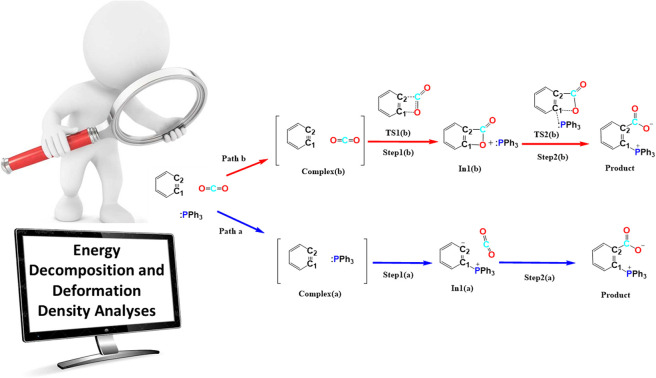

## 1. Introduction

Efficiency in the development of reaction mechanisms with the participation of triphenylphosphine derivatives remains one of the main goals of organic chemistry owing to their versatility in promoting complex transformations. Energy Decomposition Analysis (EDA) has been instrumental in elucidating key interactions in transition states and intermediates, providing a deeper understanding of selectivity in organic reactions.^1–5^ EDA is widely used in organic chemistry to break down the total interaction energy between reacting species into several components, such as electrostatic, exchange, polarization, and charge transfer contributions.^6^ These methods quantize the individual contributions of factors affecting reactivity, reaction mechanisms, and product formation.^7^ Thus, the forces operating are more clearly understood. Most importantly, EDA allows the differentiation between various interaction types that influence both the stability of the reaction's intermediates and transition states.^8^ A possible example in that direction is catalysis: EDA can identify crucial interactions between catalysts and substrates useful in finding optimal reaction conditions or in the design of more efficient catalytic systems.^9–11^ In any case, EDA holds a special position in the investigation of electron transfer processes, since it is able to dissect the role of charge transfer in the case of reactions involving radical or ionic intermediates. The EDA methods have also been applied to the analysis of other reaction types, including noncovalent interactions, hydrogen bonding, and π–π stacking in organic synthesis. The capability to visually quantify such interactions has become important in designing new organic materials, including catalysts and molecular devices. Because of this, EDA has become a very integral part of mechanistic studies in organic chemistry due to the view it presents about the molecular nature of interactions and its power as an approach to improvements in efficiency and selectivity in chemical reactions.^12–16^

Understanding the mechanisms of CO_2_ transformations is not only important for the development of green chemistry but also for organic chemists to develop efficient strategies for carbon incorporation. Mechanistic studies in CO_2_ transformation, play a critical role in environmental sustainability, economic viability, and the advancement of green chemistry.^17,18^ For further details, researchers often look into mechanistic research using tools like Density Functional Theory (DFT) calculations to model and predict these catalytic processes, driving progress in this field. Despite its broad utility, EDA has been underutilized in understanding the mechanistic pathways of CO_2_ transformations, especially in the context of triphenylphosphine derivatives. This work tries to fill this gap by using EDA to provide mechanistic insights and guide substituent design. However, a literature review shows that in some limited investigations, the EDA has been applied very successfully. Notably, the absolutely localized molecular orbital (ALMO)-EDA approach has been applied to evaluate electronic interactions in catalytic systems involving CO_2_, examining factors like polarization, charge transfer, and dispersion that contribute to the reaction's efficiency.^12^ Also, another study utilizes the ALMO-EDA (solv) approach, incorporating solvation effects to analyze intermolecular interactions in CO_2_ reduction catalysts. This method allows researchers to se*para*te interaction energies into components—such as electrostatics, polarization, and charge transfer—thereby offering insights into catalyst behavior in solution and the stabilization of intermediates involved in CO_2_ reduction.^19^

Beyond CO_2_ transformation, triphenylphosphine derivatives are critical in several organic transformations like Wittig reactions and cross-coupling, thereby underlining their importance in the design of reactions and catalysis.^20^ Their role in developing more sustainable chemistry processes is increasingly relevant as researchers look for efficient ways to convert CO_2_ into commercially valuable compounds.^21–24^

Lin He and coworkers reported the synthesis of some zwitterionic phosphonium salts *via* the reaction of benzynes, some phosphine derivatives, and CO_2_.^24^ They proposed two paths (a, b) for the reaction, and regarding the experimental facts, they reported that benzyne first undergoes a [2 + 2] reaction with CO_2_ to generate an intermediate having a four-membered ring. Then owing to the high ring strain of the four-membered ring, the intermediate undergoes a ring-opening reaction by a nucleophilic attack of triphenylphosphines. The experimental observations in this report were an inspiration for us to study the reported mechanistic paths and also the substituent effects on the mechanism. Thus, in this study, we provide a comprehensive computational analysis of the reaction mechanisms involving PPh_3_ derivatives, including electron-releasing and withdrawing group, benzyne, and CO_2_, with a focus on two competing pathways: one involving direct C–P bond formation and the other progressing through a [2 + 2] cycloaddition intermediate. By leveraging EDA and deformation density methodologies, we systematically investigate the influence of substituent effects on transition state stability and intermediate formation. Notably, we explore how electron-withdrawing and electron-donating groups impact repulsive (Δ*E*_rep_) and attractive (Δ*E*_orb_, Δ*E*_els_) interaction energies, offering insights into the electronic and steric factors that govern the reactivity and selectivity of PPh_3_ derivatives in complex organic reactions. The results not only advance the mechanistic understanding of PPh_3_-based transformations but also suggest strategic modifications to enhance reaction efficiency and selectivity. Furthermore, the results illustrate the ability of EDA and deformation density methodologies in studying CO_2_ transformation mechanisms. This work provides a valuable foundation for future studies aimed at designing PPh_3_ derivatives tailored for specific organic transformations, potentially broadening the scope of PPh_3_ applications in CO_2_ transformations.

## 2. Computational details

Several methods have been worked out for approximating the exchange-correlation functional in Kohn–Sham DFT, including a division into local and nonlocal classes. Local spin-density approximation means a functional that depends only on the local values of the spin densities. However, LSDA is not sufficiently accurate for many applications, and the inclusion of some fraction of orbital-dependent nonlocal Hartree–Fock exchange led to the so-called hybrid functionals, including M06 and M06-2X.^25^ These hybrid functionals are distinguished by the % Hartree–Fock exchange, denoted X here. Several previous studies appeared to demonstrate that this family of hybrid functionals, M06, had better performance than the B3LYP in most applications involving main-group thermochemistry, energy barriers for reactions, and noncovalent interactions. The M06 family has been applied to both closed-shell and open-shell systems, accounting for the ground-state spin quantum number as well as excited states of atoms.^26,27^ The geometries of reactants, TS, and products were optimized by M06-2X/def2SVP^27,28^ using the Gaussian 09 package.^29^ Frequency calculations were performed on all structures at 298 K to confirm the minimum of the potential energy surface. The TS structures were obtained using Schlegel's synchronous transit-guided quasi-Newton (STQN) method to ensure that just one imaginary frequency existed for the calculated TSs. Moreover, the Intrinsic Reaction Coordinate (IRC) has been done to confirm the TSs are correctly assigned.^30^ The solvent effects were evaluated in tetrahydrofuran (THF) as the solvent by a conductor-like polarizable continuum model (CPCM).^31^ Single-point energy calculations have also been performed at the M06-2X/def2tzvp level of theory for further improvement in the accuracy of the results.

Generally, the activation energy (Δ*E*^≠^) of the studied transition states (TSs) could be decomposed into the distortion energy (Δ*E*_dist_) of the involved fragments and the interaction energy (Δ*E*_int_) between them (eqn (1)) using the distortion–interaction model.^6,32^1
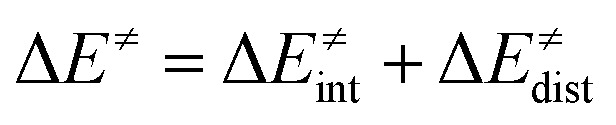


Based on the energy decomposition analysis (EDA) method using dispersion-corrected DFT strategy (or the so-called sobEDA),^33^ the interaction energy (Δ*E*_int_) between the involved segments was divided into six terms including electrostatic (Δ*E*_els_), exchange-reciprocal (Δ*E*_*x*_ + Δ*E*_rep_), orbital interaction (Δ*E*_orb_) and the sum of the DFT correlation energy (Δ*E*_DTFc_) and dispersion interaction (Δ*E*_dc_) reflects coulomb correlation (Δ*E*_c_) terms (eqn (2)). Thus, with respect to eqn (3), the overall activation energy (Δ*E*^≠^) is composed of the portions of steric effects (Δ*E*_steric_), electronic effects (Δ*E*_elec_), and Coulomb correlation effects (Δ*E*_c_) in the transition state.

2 Δ*E*_int_ = Δ*E*_c_ + Δ*E*_rep_ + Δ*E*_els_ + Δ*E*_orb_ + Δ*E*_DFTc_ + Δ*E*_dc_

3
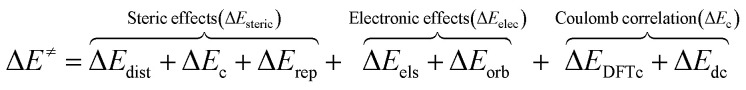


The other concept utilized for an explanation of the observed energies in transition states is deformation density.^34,35^ This concept describes the shifts in electron density that occur when atoms interact to form a molecule, capturing the redistribution of electron density that distinguishes bonded atoms from those that are separate. Deformation density (Δ*ρ*^Total^) can be decomposed into various components, with Pauli deformation density (Δ*ρ*^Pauli^) and orbital deformation density (Δ*ρ*^Orb^) being among the most informative (eqn (4)).

4 Δ*ρ*^Total^ = Δ*ρ*^Orb^ + Δ*ρ*^Pauli^

Δ*ρ*^Pauli^ is due to electron repulsion in terms of kinetic energy pressure and steric exclusion, while Δ*ρ*^Orb^ causes orbital relaxation, mixing, and consequent electron density redistribution. Fakhraee and Azami^36^ specified kinetic energy pressure as a measurable form of steric repulsion, while Frenking *et al.*^37^ associated Δ*ρ*^Pauli^ with like-spin electron repulsive interactions and equated Δ*ρ*^Orb^ with orbital mixing which leads to bonding rearrangements. Together, the reported findings underpin a mechanistic picture wherein the interaction between Δ*ρ*^Pauli^ and Δ*ρ*^Orb^ determines repulsive and attractive forces governing chemical bonding and material properties. The two-faced perspective supports analyses ranging from interpreting metal stacking fault energies to describing intermolecular interactions in molecular clusters.

These components are highly valuable when analyzed through the Natural Orbitals for Chemical Valence (NOCV) theory.^38,39^ NOCV theory systematically dissects electron density to reveal bonding interactions, constructing natural orbitals that optimally represent chemical bonding. In this framework, Δ*ρ*^Pauli^ arises primarily due to constraints from the Pauli exclusion principle, showing electron density redistribution as electrons with the same spin repel each other to avoid overlap, thereby preserving spatial separation. Meanwhile, Δ*ρ*^Orb^ accounts for the changes in electron density due to the interaction of orbitals as a result of bonding and electron delocalization. This shows those areas of space where electrons have either concentrated or depleted due to the formation of bonds or simple overlap, whether a sigma or pi bond it is. Deformation density components allow for a more instructive view on the nature of molecular bonding both qualitatively and quantitatively. The research within the density functional theory with the use of NOCV theory and deformation density decomposition has been developed in understanding specific interactions affecting the stability of a molecule and bonding energy accordingly.^40^ EDA analysis and deformation density components were evaluated using the MultiWFN 3.8.^41^

## 3. Results and discussion

**Fig. 1:**
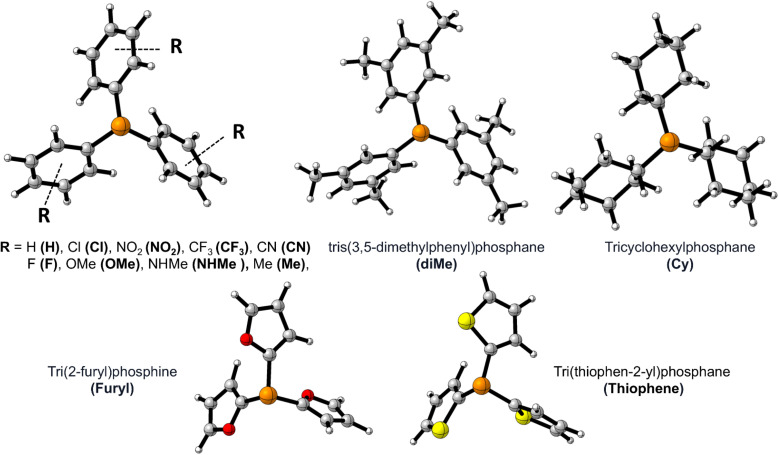
The optimized structure of PPh_3_ derivatives; and the applied abbreviation during the studies in bolded form in parentheses.

**Fig. 2:**
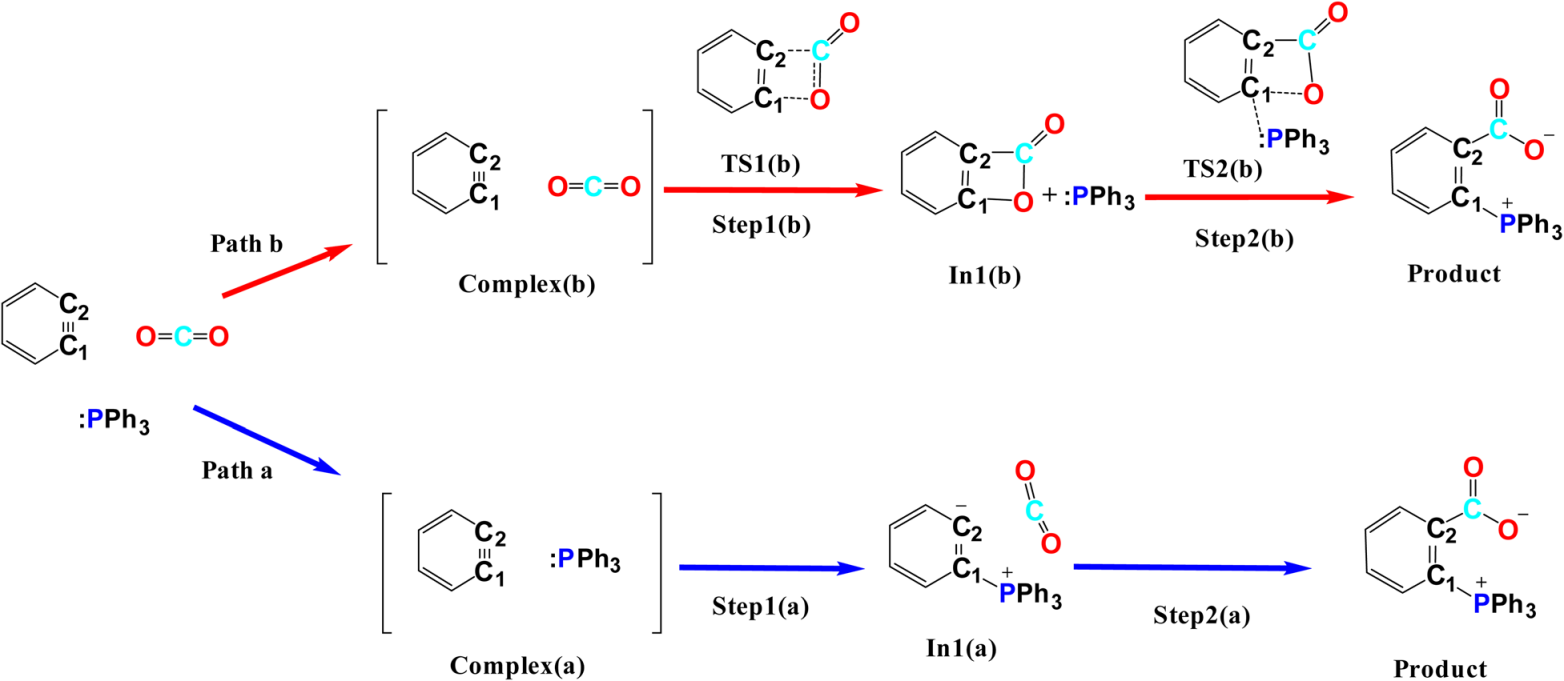
The studied mechanisms a and b for the reaction.

**Fig. 3:**
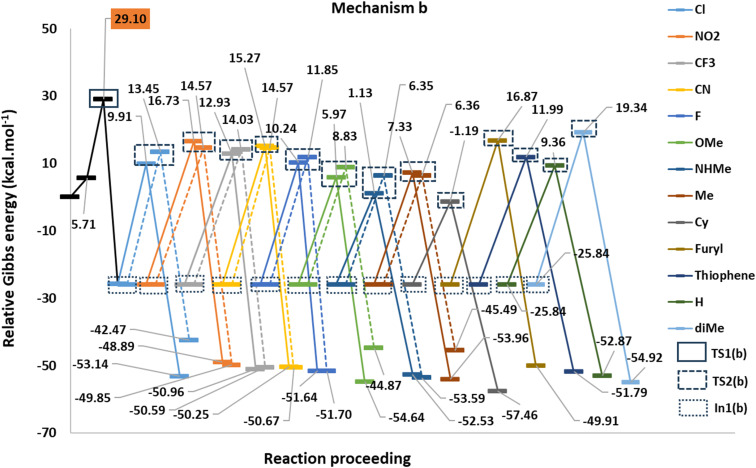
The PED of the mechanism b.

Fig. 1 shows the studied triphenylphosphine derivatives in the reaction. Fig. 2 depicts both considered mechanisms a and b in which solid and dashed lines depict the progress of the reaction for *para* and *meta* substitutions, respectively. Fig. 3 and 1(S)† depict the corresponding Potential Energy Diagrams (PED) for mechanism b and a, respectively, in which the total Gibbs energies of CO_2_, PPh_3_, and benzyne are considered as the zero state, thus the energies of the other species are abstracted from the zero state.

Path a progresses through forming a complex (complex(a)) of benzyne structure and triphenylphosphine derivatives, then, zwitterion In1(a) (depicted in solid rectangular boxes in the PED of Mechanism a), suffering a negatively charged carbon atom, is formed *via* forming a C–P bond in Step1(a). Step2(a) includes developing a C–C bond between the carbon atom of CO_2_ and the C2 atom of In1(a). None of these steps in Path a do not pass transition states, because they go through an electrostatic interaction between charged or partially charged atoms. Thus, our attempts to find corresponding transition states were not successful. Path b initiates by forming a complex between benzyne and CO_2_ (complex(b)). Step1(b) is a [2 + 2] cycloaddition reaction which leads to In1(b) (depicted in dotted rectangular boxes in the PED of Mechanism b) after descending of TS1(b) (depicted in a solid rectangular box in the PED of Mechanism b). In Step2(b) the apt four-membered ring of In1(b) passes through TS2(b) (depicted in dashed rectangular boxes in the PED of Mechanism b) by nucleophilic attack of the phosphorous atom of PPh_3_ to C1 atom, the final product is the outcome of this step. Table 1(S)† shows the calculated thermodynamic and kinetic parameters for the mechanisms.

### 3.1 HOMO/LUMO band gaps

**Fig. 4:**
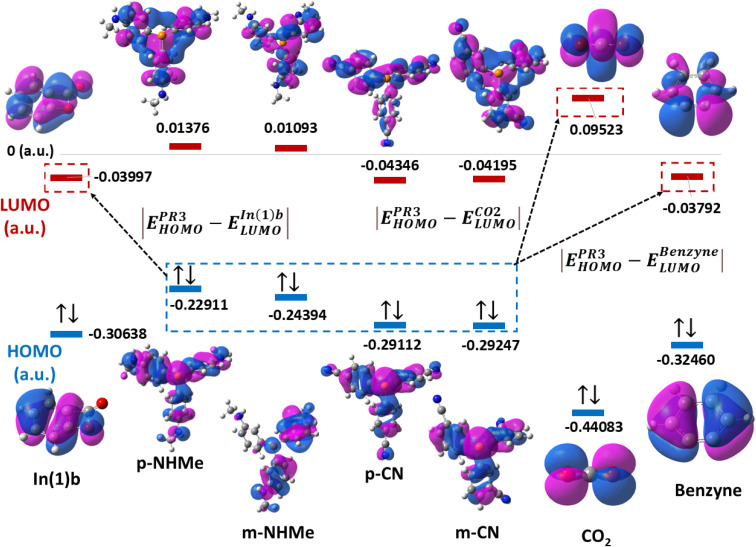
HOMO/LUMO band gaps of CO_2_, benzyne, and In(1)b in p/mCN and p/mNHMe derivatives.

Based on the Frontier Orbital Theory (FMO), the reaction can be considered as an interaction between high occupied orbital (HOMO) of PR_3_ derivatives and low unoccupieolecular orbital (LUMO) of CO_2_, benzyne or In(1)b. Table 2(S) shows the calculated HOMO/LUMO values for the involved species (in a.u.). In this table the energy gaps between 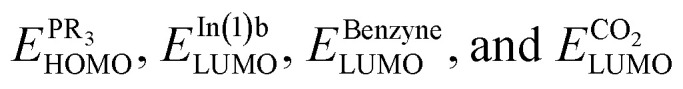 are useful criteria to find whether the reaction progresses through Path a or b. In fact, the absolute value of the energy gap 
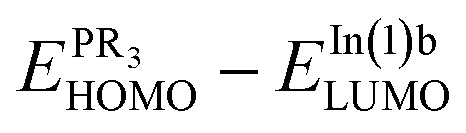
 is lower 
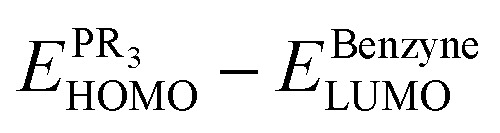
 for all derivatives. On the other hand, the values of the energy gap of 
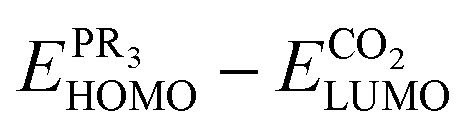
 is higher than the two others. Thus, it is unlike the reaction going through a reaction between PR_3_ derivatives and CO_2_. Also, the reaction between PR_3_ and In(1)b is more favored. Fig. 4 illustrates a schematic picture for HOMO/LUMO orbitals of CO_2_, benzyne, and In(1)b in p/mCN (as a sample of electron-withdrawing groups) and p/mNHMe (as a sample of electron-releasing groups).

**Fig. 5:**
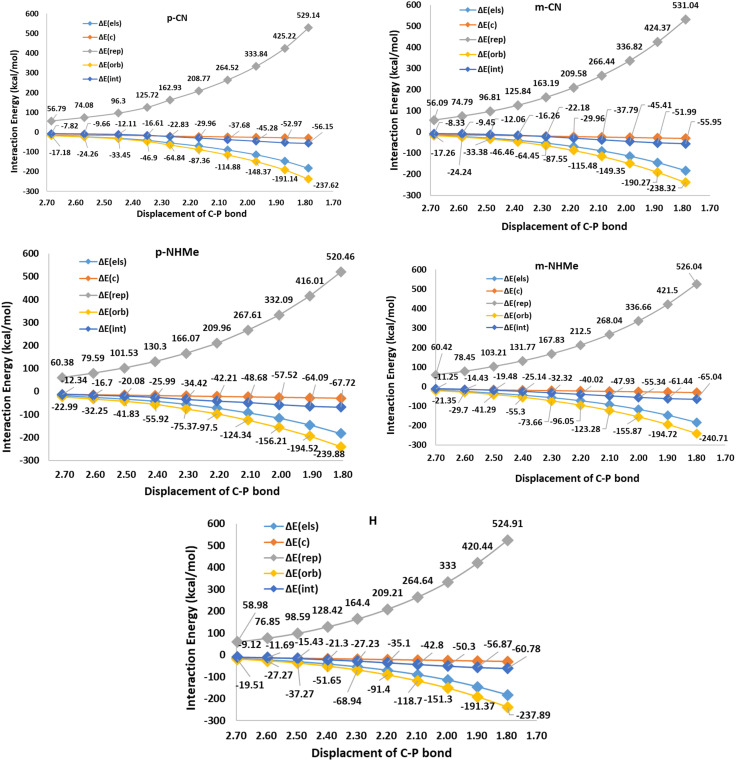
The EDA analyses for triphenylphosphine (H) and the derivatives p/mCN (as a sample of electron-withdrawing groups), p/mNHMe (as a sample of electron-realizing groups) in the first step of mechanism a.

In respect to the reported experimental fact,^24^ benzyne can react with CO_2_ in the absence of triphenylphosphine. On the other hand, In1(b) is more stable (−25.84 kcal mol^−1^) than all derivatives of In1(a). It can be concluded that forming zwitterion In1(a), which accomplishes charge developing, is not a possible approach in the reaction. Thus, C–P bond formation in Path a is not an appropriate phenomenon which is an interesting question. We decided to study the phenomenon by EDA analyses. For this purpose, some triphenylphosphine derivatives, having electron-withdrawing and electron-releasing groups are chosen and relaxed scans of the C–P bond between benzyne and PPh_3_ are performed, perturbing the optimized C–P bond by 0.1 Å in each step for 10 times. Then the geometry of each step (10 steps for each derivative) was extracted as an xyz format file for sobEDA analyses. Fig. 5 depicts the performed analyses for p/mCN and p/mNHMe substituents also Fig. 2(S)† depicts the analyses for the others.

### 3.2 EDA and deformation density analyses

Fig. 5 shows that Δ*E*_rep_ and Δ*E*_orb_ are the first and the second effective factors on Δ*E*_int_. Also, the growth of Δ*E*_rep_ in CN, as an electron-withdrawing substitute, is more considerable than NHMe as an electron-releasing one. Generally, it can be concluded that the C–P bond formation causes more instability due to growing repulsion interactions. This negative effect is more remarkable in electron-withdrawing groups such as CN and NO_2_. When it comes to Δ*E*_orb_, as a reducing factor on Δ*E*_int_, the electron-releasing groups induce more decline during the C–P bond development. However, Δ*E*_orb_ cannot compensate for the remarkable increase in Δ*E*_rep_. Another interesting observation can be concluded by the compression of *meta* and *para* positions. Fig. 5 and 2(S)† depict that rising Δ*E*_rep_ values in *meta* positions are more significant. These reveal that the substituents in the *meta* position are an effective hindrance in C–P bond evolution. The comparison of diMe, Furyl, Thiopene, and Cy (Fig. 2(S)†) reveals that the effects of diMe, Furyl, and Cy are similar to electron-releasing groups, whereas Thiopen plays as a potent electron-withdrawing group. Also, as a notable observation among all of the studied PPh_3_ derivatives, by C–P developing the growth of Δ*E*_rep_ in Furyl and Thiopen are the minimum and maximum values, respectively. In conclusion, regarding the discussed studies, can be deduced that the progress of the reaction *via* Path A is not favored, because the increase in Δ*E*_rep_ value during C–P development is a limiting factor, so other favorable factors such as Δ*E*_orb_ and Δ*E*_els_ cannot offset the unfavorable nature of Δ*E*_int_.

**Fig. 6:**
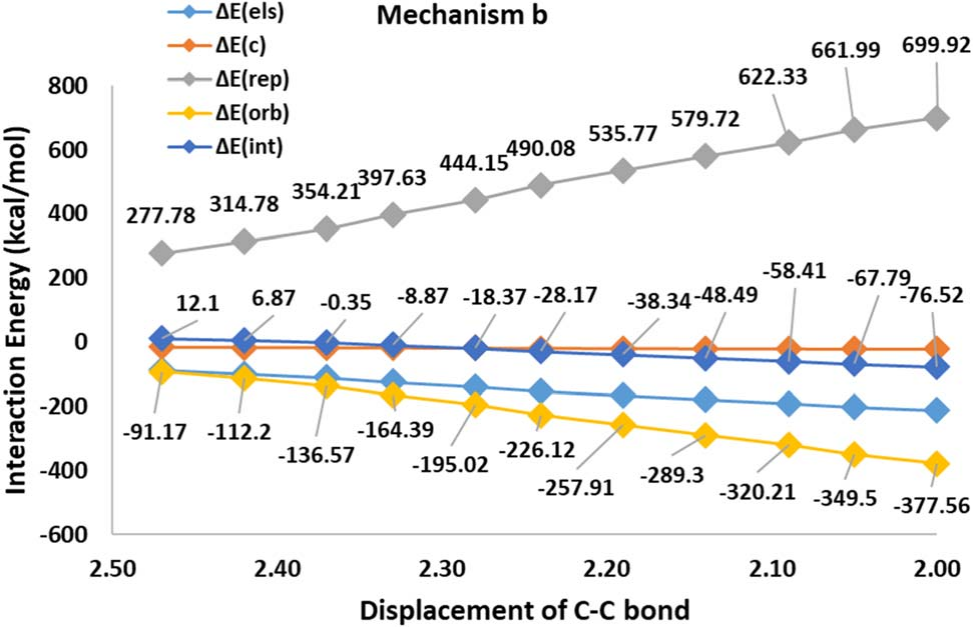
The EDA analyses for the first step of Path b.

**Fig. 7:**
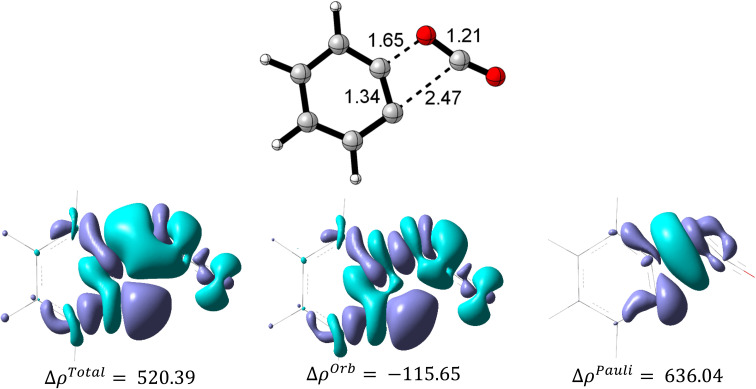
The optimized structure of TS1(b) and the corresponding deformation density map components (isovalue = 0.005).

The first step in mechanism b is In1(b) formation *via* a [2 + 2] cycloaddition reaction in which the reaction goes through a pericyclic transition state. Fig. 6 depicts the EDA study for mechanism b which reveals that both Δ*E*_rep_ and Δ*E*_orb_ have greater growth than the first step in mechanism a. Also, Δ*E*_els_ values have a more significant decline than the corresponding values in In1(a) formation in mechanism a. The sum of Δ*E*_els_ and Δ*E*_orb_ has a compensatory effect in Δ*E*_rep_ which decreases Δ*E*_int_ more remarkably than that one in mechanism a. Thus, a decrease in the Δ*E*_int_ values, as a favored factor to progress the reaction during C–C bond formation in mechanism b, is more meaningful than C–P bond development in mechanism a. It can be considered as a good justification to progress the reaction *via* mechanism b and not mechanism a. Fig. 7 shows the optimized TS1(b), corresponding deformation density maps and the calculated values. It is clear that Δ*ρ*^Pauli^ value has a more significant role in the Δ*ρ*^Total^.

**Fig. 8:**
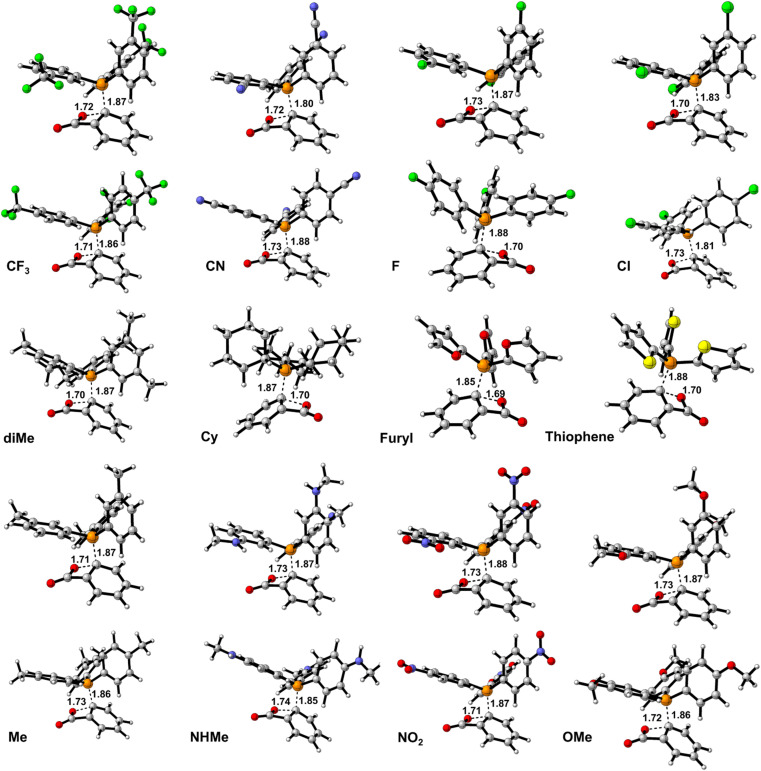
The calculated transition states for the Step2(b).

**Fig. 9:**
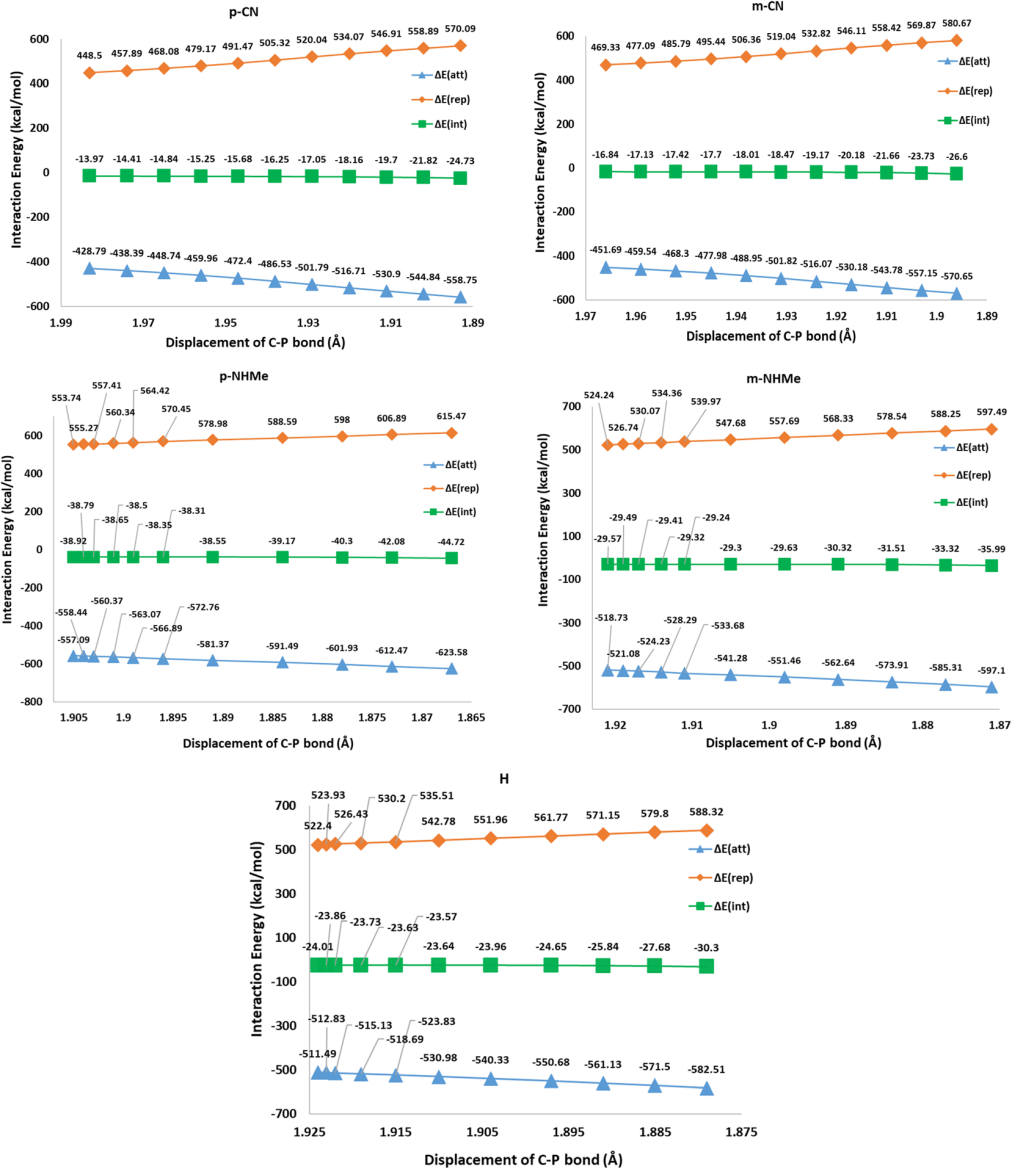
The EDA analyses H, p/mCN, and p/mNHMe in the Step2(b).

Step2(b) is accomplished *via* a nucleophilic attack of the phosphorous atom of PPh_3_ to the carbon atom of In1(b) resulting in the formation of the final product. Fig. 8 shows the calculated transition states for this step. The step progression was also investigated *via* the EDA method. Generally, two factors can be considered effective factors in Δ*E*_int_ of the intermediate formation in which one factor decreases and another factor grows the Δ*E*_int_. Table 3 (S)† shows the calculated EDA parameters for all the studied derivatives. The parameters such as Δ*E*_c_, Δ*E*_elec_, and Δ*E*_orb_, as attraction parameters, decline Δ*E*_int_ values, however, Δ*E*_rep_ has an inverse effect which increases Δ*E*_int_ values. On the other hand, Δ*E*_rep_ values are more powerful than each attraction parameter, individually. It can be considered all of the attraction parameters as Δ*E*_att_ = Δ*E*_c_ + Δ*E*_elec_ + Δ*E*_orb_ which against Δ*E*_rep_, has a stabilizing effect on Δ*E*_int_ values. Fig. 9 and 3(S)† depict the calculated EDA analyses for H, p/mCN and p/mNHMe. Concerning the parameters of H, it is clear that Δ*E*_int_ of p/mCN and p/mNHMe are more positive and more negative values, respectively. On the other hand, p/mNHMe possesses lower Δ*E*_att_ than the corresponding values of p/mCN. Notably, Δ*E*_rep_ for the p/mNHMe has larger values than H and p/mCN. Fig. 3(S)† depicts the same trend for other derivatives. As a result, electron-releasing groups cause an increase in Δ*E*_rep_ and a decrease in Δ*E*_att_ values, simultaneously. However, the role of electron-withdrawing groups such as CN, NO_2_, CF_3_, *etc* in altering the Δ*E*_rep_ and Δ*E*_att_ values is not as significant as that of electron-donating groups such as NHMe, OMe, Me, *etc.*

When it comes to the comparison of *meta* and *para* positions, it is obvious that the changes in Δ*E*_rep_ values for *meta* and *para* positions for electron-withdrawing and electron-releasing groups are different. Indeed, in electron-withdrawing groups changing the position from *para* to *meta* leads to a growth in Δ*E*_rep_ values. However, the change for electron-releasing groups has inverse effects on the values. In the case of Δ*E*_att_ values the trend is *vice versa* of Δ*E*_rep_. As a matter of fact, changing *para* to *meta* positions causes a decline and a growth in the Δ*E*_att_ values in electron-withdrawing and electron-releasing groups, respectively.

**Fig. 10:**
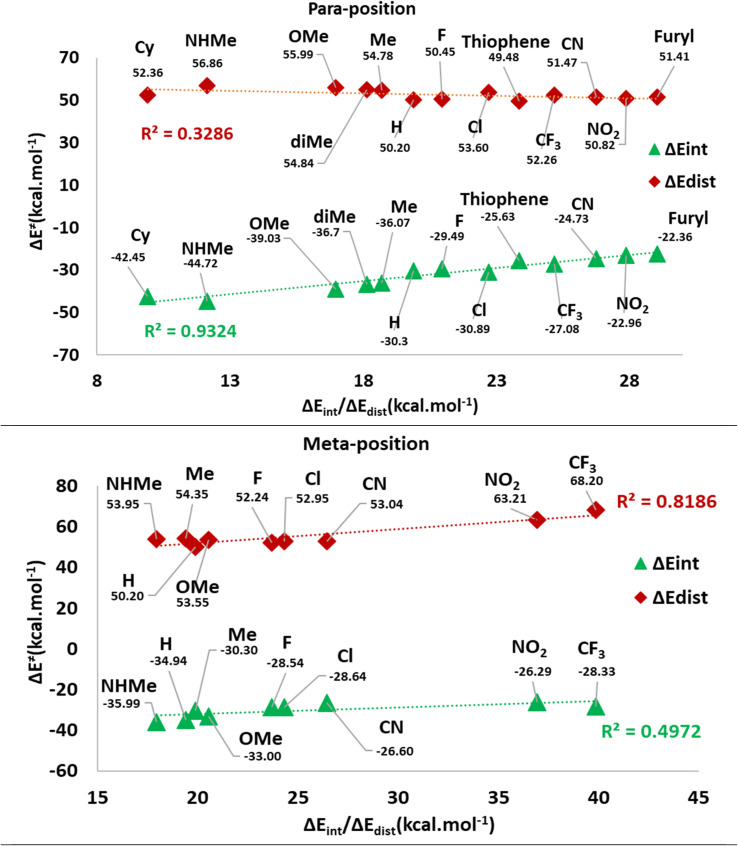
The plots of Δ*E*^≠^ against the Δ*E*_int_ and Δ*E*_dist_ values in the *meta* and *para* positions.

Table 1 shows the calculated distortion (Δ*E*_dist_) and interaction (Δ*E*_int_) parameters for the TS2(b). Fig. 10 illustrates the plots of Δ*E*^≠^ against the Δ*E*_int_ and Δ*E*_dist_ values in two separate categories for the *meta* and *para* positions of substitutions. These plots show a possible correlation between Δ*E*^≠^ and Δ*E*_int_ or Δ*E*_dist_ values. Indeed, the *meta*-position plot includes an acceptable correlation between Δ*E*^≠^ and Δ*E*_dist_ values (*R*^2^ = 0.82). However, the *para*-position one depicts a remarkable correlation between Δ*E*^≠^ and Δ*E*_int_ values (*R*^2^ = 0.93). Thus, the observed Δ*E*^≠^ values corresponding to *para* substituents are affected by Δ*E*_int_, while *meta* position groups have their effects on Δ*E*^≠^ values by influence on Δ*E*_dist_. It may be related to the direct electronic effects of *para* substituents through the direct resonance effect which causes a higher role of Δ*E*_int_ values for this position in the C–P bond developing in TS2(b). However, in mete substituents, Δ*E*_dist_ is the determining factor because the direct resonance effect has diminished. Another fact is that Δ*E*_dist_ values in *meta*-position groups generally possess higher values than *para* position.

**Table 1:** Δ*E*^≠^, Δ*E*_int_ and Δ*E*_dist_ values for the TS2(b) (all in kcal mol^−1^)

	*R*	Δ*E*^≠^	Δ*E*_int_	Δ*E*_dist_		Δ*E*^≠^	Δ*E*_int_	Δ*E*_dist_
*Para*-position	NO2	27.86	−22.96	50.82	*Meta*-position	36.92	−26.29	63.21
	CN	26.74	−24.73	51.47		26.44	−26.6	53.04
	CF3	25.18	−27.08	52.26		39.87	−28.33	68.20
	F	20.96	−29.49	50.45		23.70	−28.54	52.24
	H	19.90	−30.3	50.20		19.90	−30.3	50.20
	Cl	22.71	−30.89	53.60		24.31	−28.64	52.95
	Me	18.71	−36.07	54.78		19.41	−34.94	54.35
	OMe	16.96	−39.03	55.99		20.55	−33	53.55
	NHMe	12.14	−44.72	56.86		17.96	−35.99	53.95
	Thiophene	23.85	−25.63	49.48		—	—	—
	Furyl	29.05	−22.36	51.41		—	—	—
	Cy	9.91	−42.45	52.36		—	—	—
	diMe	18.14	−36.7	54.84		—	—	—

**Fig. 11:**
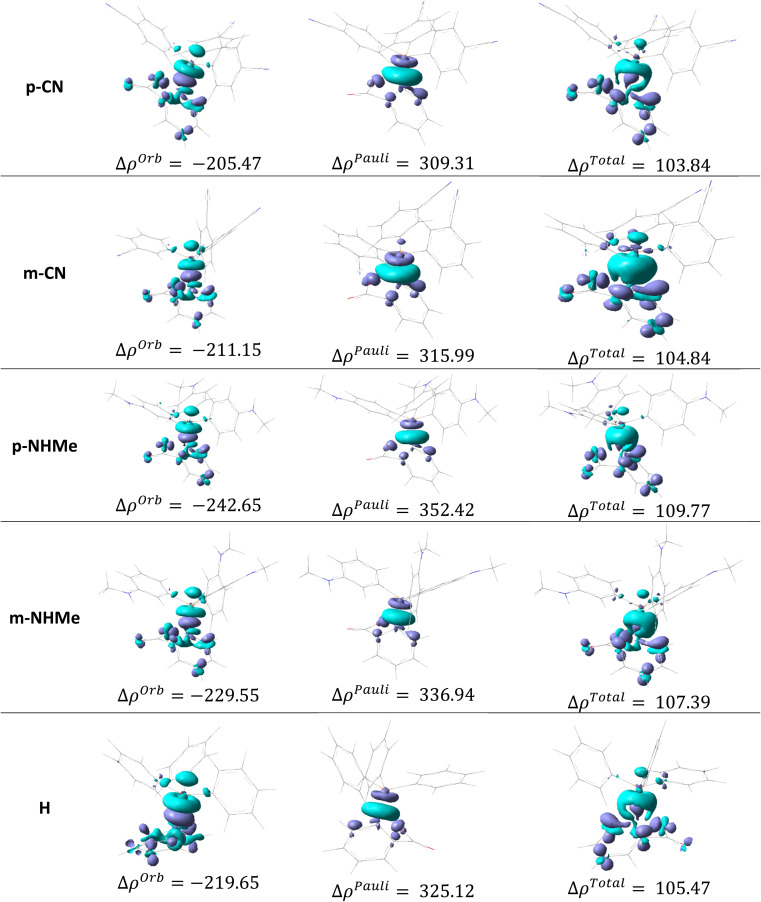
The deformation density components for H, p/mCN and p/mNHMe derivatives in TS2(b).

Fig. 11 and 4(S)† depict the deformation density components for H, p/mCN and p/mNHMe derivatives in TS2(b). In both p/mCN and p/mNHMe the Δ*ρ*^Pauli^ is a higher value than Δ*ρ*^Orb^ which has more effects on the Δ*ρ*^Total^. On the other hand, it can be found that the Δ*ρ*^Total^ and Δ*ρ*^Orb^ in p/mNHMe groups possess higher and lower values than the p/mCN ones, respectively. Thus, with respect to H, it can be concluded that electron-releasing groups vary all the components of the deformation density more remarkably than electron-withdrawing groups. When it comes to the comparison of *para* and *meta* positions, again considering the H, it is conceivable that *para* derivatives result in more variation for the components in both electron-releasing and electron-withdrawing groups. Indeed, in relation to the NHMe, Δ*ρ*^Orb^ value from −219.65 kcal mol^−1^ in H has been reached to −229.55 kcal mol^−1^ (ΔΔ*ρ*^Orb^ = −9.90 kcal mol^−1^) in *meta* position, but for the *para* position the value reaches −242.65 kcal mol^−1^ (ΔΔ*ρ*^Orb^ = −23 kcal mol^−1^). Also, the Δ*ρ*^Pauli^ value in H changes from 325.12 kcal mol^−1^ to 336.94 kcal mol^−1^ in *meta* position (ΔΔ*ρ*^Pauli^ = 11.82 kcal mol^−1^) and to 352.42 kcal mol^−1^ in *para* position (ΔΔ*ρ*^Pauli^ = 27.30 kcal mol^−1^). Similar to NHMe, for CN group variations in *meta* position include ΔΔ*ρ*^Orb^ = 8.5 kcal mol^−1^ and, ΔΔ*ρ*^Pauli^ = −9.13 kcal mol^−1^, while in the *para* position, the variations are ΔΔ*ρ*^Orb^ = 14.18 kcal mol^−1^ and, ΔΔ*ρ*^Pauli^ = −15.81 kcal mol^−1^. The same trend can be deduced in other derivatives.

Investigation of possible correlation between deformation density components and Δ*E*^≠^ values of TS2(b) inFig. 12 reveals that a good correlation is observable in Δ*E*^≠^*vs.* Δ*ρ*^Pauli^ (*R*^2^ = 0.93) and Δ*ρ*^Orb^ (*R*^2^ = 0.94), in *para* positions. However, the correlation with the Δ*ρ*^Total^ (*R*^2^ = 0.65) is not acceptable. In the *meta* position, all of the correlations are not satisfactory. Indeed, it seems that the substituted derivatives in *para* positions enforce their electronic effects as Δ*ρ*^Pauli^ and Δ*ρ*^Orb^terms more remarkably than in the *meta* position.

**Fig. 12:**
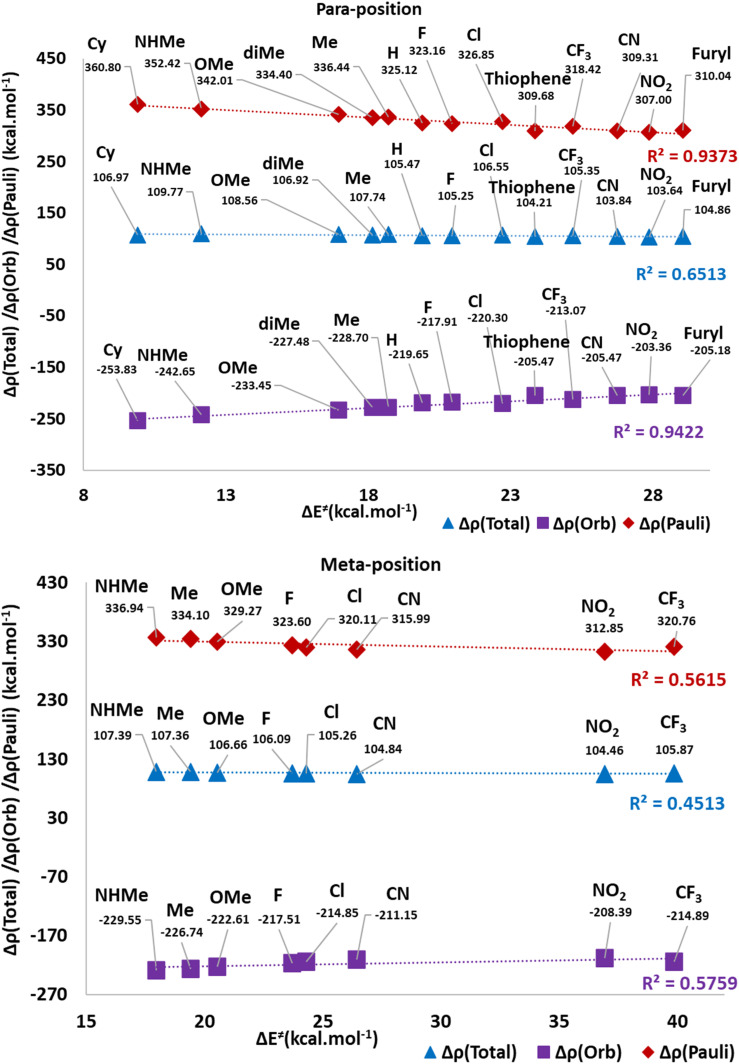
The plots of Δ*E*^≠^ against Δ*ρ*^Pauli^, Δ*ρ*^Orb^ and Δ*ρ*^Total^ in the *para* and *meta* positions.

## 4. Conclusion

This study provides an in-depth computational investigation of the reaction mechanisms involving triphenylphosphine (PPh_3_) derivatives, benzyne, and CO_2_, presenting new insights into the electronic and steric factors influencing reactivity and selectivity. Two competing mechanisms were investigated: Path a, involving direct C–P bond formation, and Path b, which progresses *via* a [2 + 2] cycloaddition. The results indicate that Path b is energetically favored, supported by more favorable interaction energies, and the enhanced stability of intermediates. Key findings from Energy Decomposition Analysis (EDA) and deformation density studies reveal C–P bond formation in Path a is hindered by substantial repulsive interactions (Δ*E*_rep_), especially for electron-withdrawing substituents like CN and NO_2_. The rise in Δ*E*_rep_ outweighs stabilizing factors such as orbital (Δ*E*_orb_) and electrostatic (Δ*E*_els_) interactions, making this pathway unfavorable. On the other hand, the formation of In1(b) through a [2 + 2] cycloaddition shows compensatory effects between attractive and repulsive forces, leading to reduced Δ*E*_int_ values. EDA analysis of TS2(b) highlights the competing influences of interaction and distortion energies. *Para*-substituents demonstrate a strong correlation between activation energy (Δ*E*^≠^) and interaction energy (Δ*E*_int_). In contrast, *meta*-substituents predominantly influence Δ*E*^≠^ through distortion energy (Δ*E*_dist_). Electron-releasing groups like NHMe enhance deformation density components such as Pauli deformation (Δ*ρ*^Pauli^) and orbital deformation (Δ*ρ*^Orb^) further stabilizing TS2(b). Thus, Deformation density analysis emphasizes the critical role of Δ*ρ*^Pauli^ and Δ*ρ*^Orb^ in determining transition state stability. *Para*-substituents exhibit stronger electronic effects, as shown by higher Δ*ρ*^Pauli^ and Δ*ρ*^Orb^ contributions compared to *meta*-substituents. This reinforces the importance of substituent design in modulating reactivity. Generally, this study identifies Path b as the preferred mechanism for PPh_3_ derivatives, supported by both thermodynamic and kinetic favorability. The findings highlight the utility of EDA and deformation density methodologies in dissecting reaction pathways, providing mechanistic aspects that guide the rational design of PPh_3_ derivatives for optimized reactivity and selectivity in CO_2_ transformation reactions.

## Data availability

The data underlying this study are available in the published article and its ESI.†

## Conflicts of interest

The authors declare no conflict of interest.

## Supplementary Material

RA-015-D5RA00804B-s001

RA-015-D5RA00804B-s002
