# Supplementary material for: Exploring CO2 activation mechanisms with triphenylphosphine derivatives: insights from energy decomposition and deformation density analyses
Source: RSC Adv. 2025 Apr 22;15(17):12917–30. doi: 10.1039/d5ra00804b (PMC12013607; doi:10.1039/d5ra00804b)
Supplement: RA-015-D5RA00804B-s001 [file RA-015-D5RA00804B-s001.pdf]

Supplementary Information (SI) for RSC Advances.
This journal is © The Royal Society of Chemistry 2025

Supporting Information 1

Exploring CO_2 Activation Mechanisms with Triphenylphosphine Derivatives: Insights from Energy Decomposition and Deformation Density Analyses

Hossein Sabet-Sarvestani*[a], Shadi Bolourian [a], Fereshteh Hosseini[a], Mohammad Javad Seddighi[b], Hamed Hosseini[a], and Hossein Eshghi [b]

[a] Department of Food Additives, Food Science and Technology Research Institute, Research Center for Iranian Academic Center for Education, Culture and Research (ACECR), Khorasan Razavi Branch, Mashhad, IRAN

[b] Department of Chemistry, Faculty of Science, Ferdowsi University of Mashhad, Mashhad, IRAN

** Correspondence to: Hossein Sabet-Sarvestani. TeleFax: ++989371411532 (E-mail address: bozorgmehr1388@gmail.com)*

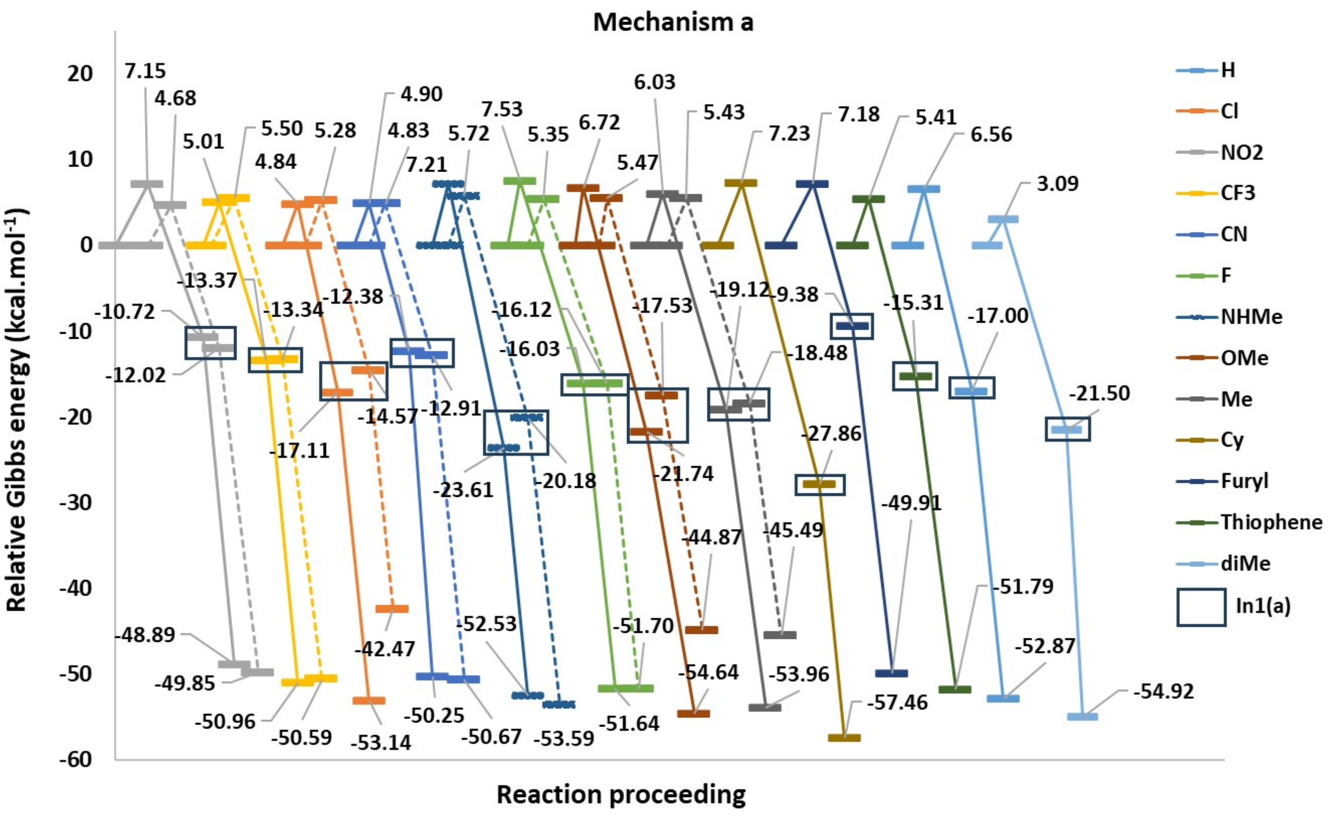

Figure 1(S). The PED of the mechanism **a**

Table 1(S). Thermodynamic and kinetic parameters of the reaction steps in both paths **a** and **b**

G	Steps	M06-2X/def2SVP										M06-2X/def2tzvp		
		Path A				Path B						Path A	Path B	
		ΔG[1]	ΔH[1]	ΔS[2]	ΔE[1]	ΔG[1]	ΔH[1]	ΔS[2]	ΔG≠[1]	ΔE[1]	ΔE≠[1]	ΔE[1]	ΔE[1]	ΔE≠[1]
H	1	-17.00	-26.81	-10.93	-27.74	-31.55	-36.18	-15.53	29.10	-37.44	28.43	-29.84	-34.60	28.43
	2	-35.88	-48.78	-43.29	-50.87	-27.03	-41.00	-46.84	35.21	-42.95	20.29	-47.99	-80.58	19.90
Cl	1(p)	-17.11	-28.24	-6.66	-24.57	-31.55	-36.18	-15.53	29.10	-37.44	28.43	-29.84	-34.60	28.43
	2(p)	-36.03	-49.20	-44.17	-51.39	-27.29	-39.16	-39.79	35.75	-41.70	22.71	-48.68	-43.36	22.50
	1(m)	-14.57	-27.88	-44.65	-29.77	-31.55	-36.18	-15.53	29.10	-37.44	28.43	-29.84	-34.60	28.43
	2(m)	-35.96	-49.58	-45.67	-51.78	-26.03	-38.29	-41.11	39.30	-40.88	24.31	-49.15	-42.70	24.04
NO_2	1(p)	-10.72	-24.83	-47.34	-26.62	-31.55	-36.18	-15.53	29.10	-37.44	28.43	-29.84	-34.60	28.43
	2(p)	-38.17	-51.45	-44.54	-53.76	-23.05	-38.00	-50.16	42.57	-39.92	27.86	-51.21	-41.31	28.30
	1(m)	-12.02	-25.62	-45.62	-27.32	-31.55	-36.18	-15.53	29.10	-37.44	28.43	-29.84	-34.60	28.43
	2(m)	-37.83	-50.67	-43.06	-52.94	-24.01	-38.01	-46.97	40.41	-39.80	36.92	-50.34	-41.32	27.32
diMe	1	-21.50	-30.61	-30.56	-33.76	-31.55	-36.18	-15.53	29.10	-37.44	28.43	-29.84	-34.60	28.43
	2	-33.42	-48.22	-49.64	-50.65	-29.08	-40.55	-38.48	30.80	-43.96	18.14	-47.74	-45.52	18.01
CF_3	1(p)	-13.37	-26.91	-45.41	-28.77	-31.55	-36.18	-15.53	29.10	-37.44	28.43	-29.84	-34.60	28.43
	2(p)	-37.59	-50.54	-43.42	-52.64	-25.12	-39.17	-47.11	38.77	-40.96	25.18	-49.95	-42.64	25.23
	1(m)	-13.34	-27.42	-47.22	-29.05	-31.55	-36.18	-15.53	29.10	-37.44	28.43	-29.84	-34.60	28.43
	2(m)	-37.25	-50.03	-42.86	-52.36	-24.74	-39.16	-48.36	39.87	-40.96	39.87	-49.71	-42.59	24.68
CN	1(p)	-12.38	-25.63	-44.47	-27.38	-31.55	-36.18	-15.53	29.10	-37.44	28.43	-29.84	-34.60	28.43
	2(p)	-37.88	-51.00	-44.02	-53.23	-24.41	-38.36	-46.77	41.11	36.76	26.74	-50.64	-41.91	26.86
	1(m)	-12.91	-26.16	-44.44	-28.03	-31.55	-36.18	-15.53	29.10	-37.44	28.43	-29.84	-34.60	28.43
	2(m)	-37.76	-50.43	-42.51	-52.58	-24.83	-38.31	-45.22	40.41	-40.16	26.44	-50.05	-41.73	26.63
F	1(p)	-16.03	-30.34	-47.98	-32.18	-31.55	-36.18	-15.53	29.10	-37.44	28.43	-29.84	-34.60	28.43
	2(p)	-35.61	-48.40	-42.91	-50.60	-25.80	-40.46	-49.17	36.09	-42.33	20.96	-48.07	-44.01	20.49
	1(m)	-16.12	-28.34	-41.00	-30.16	-31.55	-36.18	-15.53	29.10	-37.44	28.43	-29.84	-34.60	28.43
	2(m)	-35.59	-49.32	-46.08	-51.61	-25.86	-39.38	-45.36	37.69	-41.32	23.70	-48.97	-42.88	23.62
OMe	1(p)	-21.74	-33.37	-38.98	-35.16	-31.55	-36.18	-15.53	29.10	-37.44	28.43	-29.84	-34.60	28.43
	2(p)	-32.89	-47.08	-47.58	-49.51	-28.79	-42.16	-44.84	31.81	-44.21	16.96	-46.74	-45.90	16.54
	1(m)	-17.53	-30.73	-44.28	-32.67	-31.55	-36.18	-15.53	29.10	-37.44	28.43	-29.84	-34.60	28.43
	2(m)	-35.85	-48.56	-42.62	-50.81	-27.53	-41.01	-45.18	34.67	-43.03	20.55	-48.13	-44.58	20.43
NHMe	1(p)	-23.61	-37.16	-45.45	-39.03	-31.55	-36.18	-15.53	29.10	-37.44	28.43	-29.84	-34.60	28.43
	2(p)	-28.93	-42.24	-44.64	-44.73	-26.69	-41.12	-48.38	26.97	-43.30	12.14	-42.50	-45.05	12.18
	1(m)	-20.18	-32.92	-42.74	-35.03	-31.55	-36.18	-15.53	29.10	-37.44	28.43	-29.84	-34.60	28.43
	2(m)	-33.40	-47.31	-46.64	-49.83	-27.74	-41.95	-47.66	32.20	-44.41	17.96	-47.19	-43.88	19.89
Me	1(p)	-19.12	-31.88	-42.79	-33.83	-31.55	-36.18	-15.53	29.10	-37.44	28.43	-29.84	-34.60	28.43
	2(p)	-34.84	-48.03	-44.24	-50.31	-28.12	-41.63	-45.32	33.17	-43.68	18.71	-47.05	-45.01	18.22
	1(m)	-18.48	-31.15	-42.49	-33.07	-31.55	-36.18	-15.53	29.10	-37.44	28.43	-29.84	-34.60	28.43
	2(m)	-35.63	-48.70	-43.83	-50.85	-28.28	-41.57	-44.60	32.20	-43.46	19.41	-47.84	-44.77	19.22
Cy	1	-27.86	-40.57	-42.62	-42.89	-31.55	-36.18	-15.53	29.10	-37.44	28.43	-29.84	-34.60	28.43
	2	-29.60	-42.06	-41.78	-44.64	-31.62	-44.34	-42.67	24.65	-47.08	9.88	-43.44	-49.74	9.91
Furyl	1	-9.38	-22.06	-42.52	-23.83	-31.55	-36.18	-15.53	29.10	-37.44	28.43	-29.84	-34.60	28.43
	2	-49.91	-55.81	-51.26	-58.58	-24.07	-39.59	-52.05	42.71	-41.95	28.31	-54.94	-42.90	29.05
Thiophene	1	-15.31	-28.41	-43.93	-30.33	-31.55	-36.18	-15.53	29.10	-37.44	28.43	-29.84	-34.60	28.43
	2	-36.48	-49.55	-43.86	-51.98	-25.95	-39.68	-46.07	37.83	-41.86	24.10	-61.98	-42.87	23.85

1) kcal.mol^{-1}
2) cal.mol-1K^{-1}

Table 2(s). The calculated HOMO/LUMO values for the starting materials and **In(1)b**

Species	E_{HOMO} (a.u.)	E_{LUMO} (a.u.)	$\left\|E_{HOMO}^{PR3} - E_{LUMO}^{In(1)b}\right\|$	$\left\|E_{HOMO}^{PR3} - E_{LUMO}^{Benzyne}\right\|$	$\left\|E_{HOMO}^{PR3} - E_{LUMO}^{CO2}\right\|$
**In(1)b**	-0.30638	-0.03997			
**CO_2**	-0.44083	0.09523			
**Benzyne**	-0.32460	-0.03792			
**diMe**	-0.26468	0.00462	0.22471	0.22676	0.35991
**p-CF_3**	-0.28776	-0.02377	0.24779	0.24984	0.38299
**m-CF_3**	-0.28627	-0.01972	0.24630	0.24835	0.38150
**p-CN**	-0.29112	-0.04346	0.25115	0.25320	0.38635
**m-CN**	-0.29247	-0.04195	0.25250	0.25455	0.38770
**p-Cl**	-0.27532	-0.00978	0.23535	0.23740	0.37055
**m-Cl**	-0.28216	-0.01167	0.24219	0.24424	0.37739
**p-Me**	-0.26388	0.00467	0.22391	0.22596	0.35911
**m-Me**	-0.26735	0.00249	0.22738	0.22943	0.36258
**Cy**	-0.25588	0.08003	0.21591	0.21796	0.35111
**p-F**	-0.27311	-0.00631	0.23314	0.23519	0.36834
**m-F**	-0.28074	-0.00901	0.24077	0.24282	0.37597
**p-NHMe**	-0.22911	0.01376	0.18914	0.19119	0.32434
**m-NHMe**	-0.24394	0.01093	0.20602	0.20602	0.33917
**p-NO_2**	-0.29749	-0.06627	0.25752	0.25957	0.39272
**m-NO_2**	-0.29628	-0.06521	0.25631	0.25836	0.39151
**p-OMe**	-0.25425	0.00425	0.21428	0.21633	0.34948
**m-OMe**	-0.26863	0.00045	0.22866	0.23071	0.36386
**H**	-0.27097	0.00078	0.23100	0.23305	0.36620
**Thiopene**	-0.27174	-0.00606	0.23177	0.23382	0.36697
**Furyl**	-0.27228	0.01106	0.23231	0.23436	0.36751

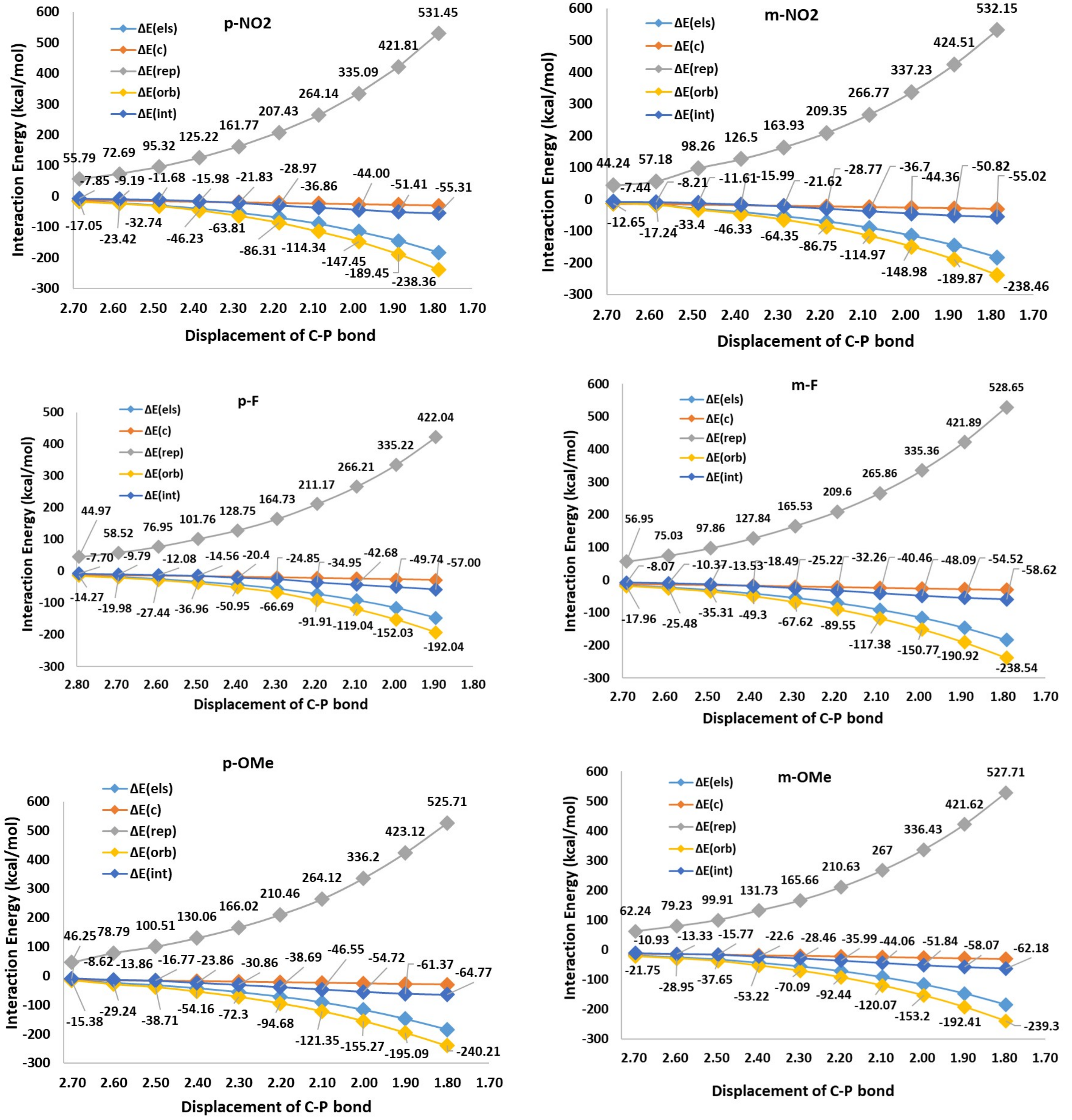
p-NO2
ΔE(els)
ΔE(c)
ΔE(rep)
ΔE(orb)
ΔE(int)
Interaction Energy (kcal/mol)
Displacement of C-P bond
600
500
400
300
200
100
0
-100
-200
-300
2.70 2.60 2.50 2.40 2.30 2.20 2.10 2.00 1.90 1.80 1.70
55.79 72.69 95.32 125.22 161.77 207.43 264.14 335.09 421.81 531.45
-7.85 -9.19 -11.68 -15.98 -21.83 -28.97 -36.86 -44.00 -51.41 -55.31
-17.05 -23.42 -32.74 -46.23 -63.81 -86.31 -114.34 -147.45 -189.45 -238.36
m-NO2
ΔE(els)
ΔE(c)
ΔE(rep)
ΔE(orb)
ΔE(int)
Interaction Energy (kcal/mol)
Displacement of C-P bond
600
500
400
300
200
100
0
-100
-200
-300
2.70 2.60 2.50 2.40 2.30 2.20 2.10 2.00 1.90 1.80 1.70
44.24 57.18 98.26 126.5 163.93 209.35 266.77 337.23 424.51 532.15
-7.44 -8.21 -11.61 -15.99 -21.62 -28.77 -36.7 -44.36 -50.82 -55.02
-12.65 -17.24 -33.4 -46.33 -64.35 -86.75 -114.97 -148.98 -189.87 -238.46
p-F
ΔE(els)
ΔE(c)
ΔE(rep)
ΔE(orb)
ΔE(int)
Interaction Energy (kcal/mol)
Displacement of C-P bond
500
400
300
200
100
0
-100
-200
-300
2.80 2.70 2.60 2.50 2.40 2.30 2.20 2.10 2.00 1.90 1.80
44.97 58.52 76.95 101.76 128.75 164.73 211.17 266.21 335.22 422.04
-7.70 -9.79 -12.08 -14.56 -20.4 -24.85 -34.95 -42.68 -49.74 -57.00
-14.27 -19.98 -27.44 -36.96 -50.95 -66.69 -91.91 -119.04 -152.03 -192.04
m-F
ΔE(els)
ΔE(c)
ΔE(rep)
ΔE(orb)
ΔE(int)
Interaction Energy (kcal/mol)
Displacement of C-P bond
600
500
400
300
200
100
0
-100
-200
-300
2.70 2.60 2.50 2.40 2.30 2.20 2.10 2.00 1.90 1.80 1.70
56.95 75.03 97.86 127.84 165.53 209.6 265.86 335.36 421.89 528.65
-8.07 -10.37 -13.53 -18.49 -25.22 -32.26 -40.46 -48.09 -54.52 -58.62
-17.96 -25.48 -35.31 -49.3 -67.62 -89.55 -117.38 -150.77 -190.92 -238.54
p-OMe
ΔE(els)
ΔE(c)
ΔE(rep)
ΔE(orb)
ΔE(int)
Interaction Energy (kcal/mol)
Displacement of C-P bond
600
500
400
300
200
100
0
-100
-200
-300
2.70 2.60 2.50 2.40 2.30 2.20 2.10 2.00 1.90 1.80 1.70
46.25 78.79 100.51 130.06 166.02 210.46 264.12 336.2 423.12 525.71
-8.62 -13.86 -16.77 -23.86 -30.86 -38.69 -46.55 -54.72 -61.37 -64.77
-15.38 -29.24 -38.71 -54.16 -72.3 -94.68 -121.35 -155.27 -195.09 -240.21
m-OMe
ΔE(els)
ΔE(c)
ΔE(rep)
ΔE(orb)
ΔE(int)
Interaction Energy (kcal/mol)
Displacement of C-P bond
600
500
400
300
200
100
0
-100
-200
-300
2.70 2.60 2.50 2.40 2.30 2.20 2.10 2.00 1.90 1.80 1.70
62.24 79.23 99.91 131.73 165.66 210.63 267 336.43 421.62 527.71
-10.93 -13.33 -15.77 -22.6 -28.46 -35.99 -44.06 -51.84 -58.07 -62.18
-21.75 -28.95 -37.65 -53.22 -70.09 -92.44 -120.07 -153.2 -192.41 -239.3

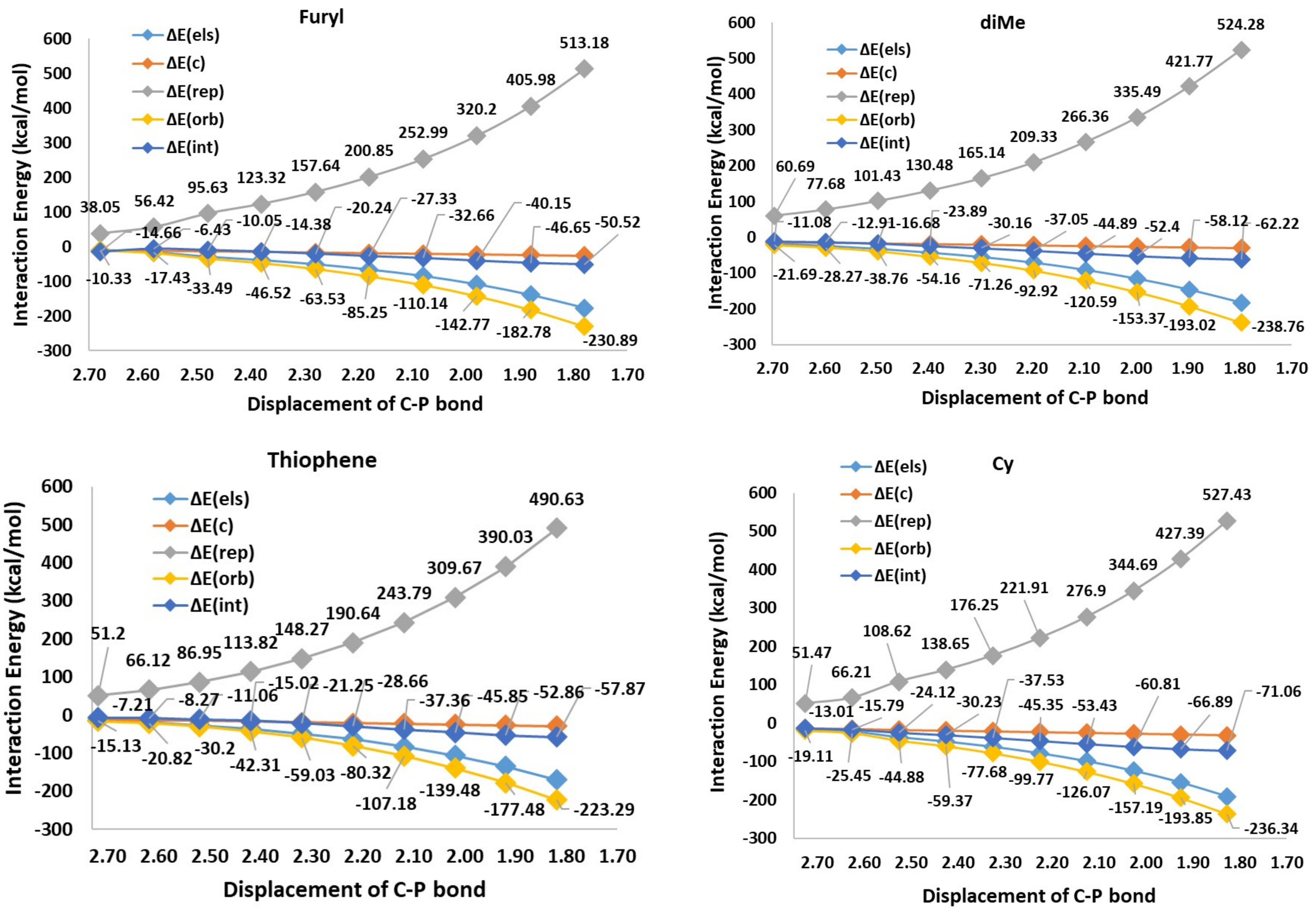

Figure 2(S) The EDA analyses for other derivatives

**Table3(S)**. The calculated energy decomposition parameters for C-P bond formation during step2(b)					
p-CN					
Displacement of C-P bond	ΔE(els)	ΔE(c)	ΔE(rep)	ΔE(orb)	ΔE(int)
1.983	-134	-162.2	448.5	-132.59	-13.97
1.974	-137.06	-165.22	457.89	-136.11	-14.41
1.965	-140.36	-168.48	468.08	-139.9	-14.84
1.956	-143.93	-172.01	479.17	-144.02	-15.25
1.947	-147.85	-175.91	491.47	-148.64	-15.68
1.938	-152.22	-180.25	505.32	-154.06	-16.25
1.929	-156.82	-184.78	520.04	-160.19	-17.05
1.92	-161.14	-189.04	534.07	-166.53	-18.16
1.911	-165.04	-192.86	546.91	-173	-19.7
1.902	-168.66	-196.37	558.89	-179.81	-21.82
1.893	-172.02	-199.58	570.09	-187.15	-24.73

**Table3(S)** (continued)					
m-CN					
Displacement of C-P bond	ΔE(els)	ΔE(c)	ΔE(rep)	ΔE(orb)	ΔE(int)
1.966	-141.31	-169.07	469.33	-141.31	-16.84
1.959	-143.85	-171.55	477.09	-144.14	-17.13
1.952	-146.68	-174.32	485.79	-147.3	-17.42
1.945	-149.79	-177.39	495.44	-150.8	-17.7
1.938	-153.29	-180.84	506.36	-154.82	-18.01
1.931	-157.29	-184.79	519.04	-159.74	-18.47
1.924	-161.59	-189.02	532.82	-165.46	-19.17
1.917	-165.68	-193.03	546.11	-171.47	-20.18
1.91	-169.41	-196.67	558.42	-177.7	-21.66
1.903	-172.86	-200	569.87	-184.29	-23.73
1.896	-176.11	-203.08	580.67	-191.46	-26.6

**Table3(S)** (continued)					
p-F					
Displacement of C-P bond	ΔE(els)	ΔE(c)	ΔE(rep)	ΔE(orb)	ΔE(int)
1.928	-158.56	-185.67	519.16	-162.79	-22.92
1.927	-159.24	-186.3	521.03	-163.17	-22.79
1.925	-160.25	-187.26	523.94	-163.93	-22.69
1.922	-161.67	-188.62	528.12	-165.14	-22.62
1.918	-163.57	-190.47	533.85	-166.95	-22.59
1.912	-166.06	-192.9	541.51	-169.62	-22.69
1.905	-169.07	-195.88	551.06	-173.36	-23.04
1.898	-172.14	-198.94	561.1	-177.83	-23.76
1.892	-175	-201.8	570.66	-182.75	-24.97
1.886	-177.55	-204.36	579.41	-188.14	-26.85
1.88	-180.09	-206.86	588.09	-194.3	-29.49

**Table3(S)** (continued)					
m-F					
Displacement of C-P bond	ΔE(els)	ΔE(c)	ΔE(rep)	ΔE(orb)	ΔE(int)
1.939	-150.26	-177.92	496.66	-154.69	-20.62
1.936	-151.62	-179.21	500.69	-155.96	-20.61
1.933	-153.32	-180.86	505.85	-157.64	-20.63
1.928	-155.4	-182.9	512.24	-159.78	-20.67
1.922	-157.95	-185.41	520.2	-162.56	-20.75
1.914	-161.15	-188.56	530.32	-166.38	-21.01
1.905	-164.88	-192.22	542.31	-171.32	-21.56
1.897	-168.59	-195.85	554.41	-176.8	-22.44
1.888	-172.03	-199.19	565.77	-182.58	-23.79
1.881	-175.26	-202.3	576.52	-188.82	-25.76
1.873	-178.35	-205.21	586.8	-195.72	-28.54

**Table3(S)** (continued)					
p-NHMe					
Displacement of C-P bond	ΔE(els)	ΔE(c)	ΔE(rep)	ΔE(orb)	ΔE(int)
1.905	-172.61	-197.71	553.74	-186.77	-38.92
1.904	-173.17	-198.2	555.27	-187.07	-38.79
1.903	-173.92	-198.88	557.41	-187.57	-38.65
1.901	-174.91	-199.82	560.34	-188.34	-38.5
1.899	-176.24	-201.11	564.42	-189.54	-38.35
1.896	-178.15	-202.99	570.45	-191.62	-38.31
1.891	-180.78	-205.57	578.98	-195.02	-38.55
1.884	-183.7	-208.42	588.59	-199.37	-39.17
1.878	-186.52	-211.15	598.00	-204.26	-40.3
1.873	-189.16	-213.67	606.89	-209.64	-42.08
1.867	-191.71	-216.06	615.47	-215.81	-44.72

**Table3(S)** (continued)					
m-NHMe					
Displacement of C-P bond	ΔE(els)	ΔE(c)	ΔE(rep)	ΔE(orb)	ΔE(int)
1.921	-160.41	-187.5	524.24	-170.82	-29.57
1.919	-161.28	-188.3	526.74	-171.5	-29.49
1.917	-162.4	-189.37	530.07	-172.46	-29.41
1.914	-163.81	-190.73	534.36	-173.75	-29.32
1.911	-165.62	-192.51	539.97	-175.55	-29.24
1.905	-168.05	-194.91	547.68	-178.32	-29.3
1.898	-171.17	-197.95	557.69	-182.34	-29.63
1.891	-174.43	-201.11	568.33	-187.1	-30.32
1.884	-177.51	-204.09	578.54	-192.31	-31.51
1.877	-180.44	-206.87	588.25	-198	-33.32
1.871	-183.21	-209.46	597.49	-204.43	-35.99

**Table3(S)** (continued)					
p-NO_2					
Displacement of C-P bond	ΔE(els)	ΔE(c)	ΔE(rep)	ΔE(orb)	ΔE(int)
1.987	-131.86	-160.15	443.02	-129.57	-12.19
1.976	-135.31	-163.55	453.64	-133.58	-12.71
1.966	-138.84	-167.06	464.61	-137.7	-13.18
1.956	-142.58	-170.77	476.25	-142.04	-13.6
1.945	-146.61	-174.78	488.94	-146.82	-14.04
1.933	-151.05	-179.19	503.03	-152.31	-14.59
1.921	-155.66	-183.74	517.81	-158.44	-15.37
1.91	-159.97	-187.98	531.79	-164.72	-16.43
1.9	-163.85	-191.78	544.54	-171.11	-17.92
1.891	-167.29	-195.11	555.95	-177.74	-20.08
1.883	-170.74	-198.42	567.43	-185.16	-22.96

**Table3(S)** (continued)					
m-NO_2					
Displacement of C-P bond	ΔE(els)	ΔE(c)	ΔE(rep)	ΔE(orb)	ΔE(int)
1.973	-139.12	-166.69	461.99	-137.62	-15.94
1.965	-141.88	-169.4	470.45	-140.74	-16.3
1.957	-144.86	-172.34	479.62	-144.11	-16.65
1.949	-148.12	-175.56	489.74	-147.82	-16.98
1.939	-151.79	-179.2	501.26	-152.11	-17.34
1.928	-156	-183.37	514.58	-157.31	-17.86
1.917	-160.48	-187.78	528.94	-163.29	-18.63
1.906	-164.73	-191.94	542.73	-169.53	-19.71
1.897	-168.6	-195.72	555.44	-175.94	-21.24
1.888	-172.18	-199.16	567.26	-182.71	-23.38
1.88	-175.49	-202.32	578.27	-189.99	-26.29

**Table3(S)** (continued)					
p-OM					
Displacement of C-P bond	ΔE(els)	ΔE(c)	ΔE(rep)	ΔE(orb)	ΔE(int)
1.914	-166	-192.11	537.75	-176.83	-32.54
1.913	-166.75	-192.79	539.85	-177.35	-32.43
1.911	-167.75	-193.72	542.78	-178.14	-32.33
1.909	-169.05	-194.97	546.67	-179.27	-32.21
1.905	-170.74	-196.63	551.92	-180.92	-32.11
1.9	-173.09	-198.93	559.32	-183.56	-32.15
1.894	-176.16	-201.92	569.15	-187.52	-32.49
1.887	-179.4	-205.08	579.77	-192.34	-33.23
1.88	-182.47	-208.05	590	-197.63	-34.48
1.874	-185.34	-210.8	599.62	-203.4	-36.36
1.867	-188.11	-213.39	608.87	-209.82	-39.03

**Table3(S)** (continued)					
m-OM					
Displacement of C-P bond	ΔE(els)	ΔE(c)	ΔE(rep)	ΔE(orb)	ΔE(int)
1.929	-155.69	-182.71	510.15	-162.59	-25.72
1.927	-156.71	-183.67	513.14	-163.45	-25.66
1.925	-158.03	-184.93	517.08	-164.66	-25.62
1.921	-159.7	-186.55	522.17	-166.28	-25.58
1.916	-161.82	-188.64	528.77	-168.5	-25.58
1.91	-164.6	-191.38	537.55	-171.72	-25.73
1.902	-167.99	-194.69	548.39	-176.12	-26.18
1.894	-171.46	-198.07	559.68	-181.21	-26.99
1.887	-174.72	-201.23	570.42	-186.7	-28.29
1.88	-177.77	-204.16	580.58	-192.68	-30.23
1.873	-180.72	-206.93	590.38	-199.39	-33

**Table3(S)** (continued)					
p-CF_3					
Displacement of C-P bond	ΔE(els)	ΔE(c)	ΔE(rep)	ΔE(orb)	ΔE(int)
1.952	-145.34	-173.11	482.23	-147.9	-18.47
1.947	-147.13	-174.83	487.6	-149.73	-18.58
1.942	-149.3	-176.94	494.21	-152.01	-18.7
1.936	-151.85	-179.44	502.09	-154.77	-18.84
1.928	-154.87	-182.41	511.51	-158.14	-19.01
1.919	-158.46	-185.95	522.86	-162.46	-19.34
1.909	-162.44	-189.87	535.63	-167.69	-19.92
1.9	-166.3	-193.66	548.22	-173.36	-20.84
1.892	-169.85	-197.12	559.93	-179.28	-22.22
1.884	-173.12	-200.28	570.83	-185.63	-24.25
1.876	-176.32	-203.32	581.48	-192.71	-27.08

**Table3(S)** (continued)					
m-CF_3					
Displacement of C-P bond	ΔE(els)	ΔE(c)	ΔE(rep)	ΔE(orb)	ΔE(int)
1.947	-147.82	-175.22	488.38	-150.24	-19.62
1.944	-149.48	-176.81	493.35	-151.91	-19.7
1.939	-151.46	-178.73	499.38	-153.97	-19.8
1.933	-153.82	-181.04	506.64	-156.48	-19.91
1.925	-156.68	-183.86	515.57	-159.67	-20.07
1.918	-160.21	-187.34	526.74	-163.93	-20.4
1.908	-164.23	-191.29	539.62	-169.24	-21.01
1.898	-168.15	-195.13	552.39	-175.01	-21.98
1.89	-171.76	-198.64	564.3	-181.08	-23.42
1.882	-175.2	-201.94	575.67	-187.59	-25.46
1.874	-178.37	-204.95	586.24	-194.7	-28.33

**Table3(S)** (continued)					
p-Me					
Displacement of C-P bond	ΔE(els)	ΔE(c)	ΔE(rep)	ΔE(orb)	ΔE(int)
1.92	-161.4	-187.93	525.71	-170.71	-29.36
1.919	-162.26	-188.71	528.17	-171.36	-29.27
1.916	-163.41	-189.8	531.57	-172.35	-29.18
1.914	-164.9	-191.23	536.07	-173.72	-29.1
1.91	-166.82	-193.11	542.02	-175.66	-29.04
1.904	-169.37	-195.62	550.08	-178.57	-29.12
1.897	-172.56	-198.73	560.31	-182.69	-29.49
1.89	-175.86	-201.96	571.12	-187.56	-30.23
1.883	-178.98	-204.98	581.47	-192.87	-31.47
1.876	-181.92	-207.79	591.26	-198.68	-33.34
1.869	-184.74	-210.45	600.71	-205.23	-36.07

**Table3(S)** (continued)					
m-Me					
Displacement of C-P bond	ΔE(els)	ΔE(c)	ΔE(rep)	ΔE(orb)	ΔE(int)
1.926	-158.34	-185.2	517.25	-166.51	-27.84
1.924	-159.38	-186.17	520.28	-167.4	-27.78
1.921	-160.76	-187.48	524.37	-168.66	-27.74
1.918	-162.49	-189.15	529.65	-170.35	-27.7
1.913	-164.67	-191.29	536.41	-172.62	-27.69
1.907	-167.49	-194.06	545.33	-175.89	-27.82
1.899	-170.89	-197.38	556.25	-180.32	-28.24
1.891	-174.34	-200.74	567.5	-185.39	-29.02
1.884	-177.56	-203.85	578.15	-190.84	-30.29
1.877	-180.53	-206.71	588.08	-196.74	-32.22
1.87	-183.4	-209.4	597.63	-203.31	-34.94

**Table3(S)** (continued)					
p-Cl					
Displacement of C-P bond	ΔE(els)	ΔE(c)	ΔE(rep)	ΔE(orb)	ΔE(int)
1.935	-153.55	-180.7	504.87	-159.15	-23.29
1.932	-154.8	-181.88	508.55	-160.29	-23.28
1.928	-156.4	-183.41	513.37	-161.84	-23.29
1.924	-158.4	-185.35	519.46	-163.86	-23.31
1.918	-160.86	-187.76	527.11	-166.5	-23.36
1.911	-163.95	-190.8	536.86	-170.13	-23.56
1.903	-167.56	-194.34	548.44	-174.84	-24.04
1.895	-171.17	-197.87	560.23	-180.13	-24.86
1.887	-174.51	-201.13	571.3	-185.76	-26.17
1.88	-177.61	-204.11	581.64	-191.85	-28.13
1.872	-180.63	-206.97	591.72	-198.67	-30.89

**Table3(S)** (continued)					
m-Cl					
Displacement of C-P bond	ΔE(els)	ΔE(c)	ΔE(rep)	ΔE(orb)	ΔE(int)
1.949	-147.15	-175.14	487.59	-150.85	-20.28
1.945	-148.67	-176.6	492.16	-152.34	-20.32
1.941	-150.56	-178.44	497.91	-154.27	-20.39
1.935	-152.83	-180.68	504.93	-156.67	-20.47
1.929	-155.59	-183.42	513.56	-159.72	-20.6
1.92	-158.99	-186.79	524.35	-163.81	-20.9
1.911	-162.87	-190.61	536.81	-168.94	-21.48
1.902	-166.69	-194.37	549.3	-174.58	-22.41
1.893	-170.27	-197.87	561.1	-180.54	-23.8
1.885	-173.58	-201.07	572.14	-186.94	-25.83
1.878	-176.71	-204.04	582.59	-193.94	-28.64

**Table3(S)** (continued)					
diMe					
Displacement of C-P bond	ΔE(els)	ΔE(c)	ΔE(rep)	ΔE(orb)	ΔE(int)
1.923	-166	-192.11	537.75	-176.83	-32.54
1.919	-166.75	-192.79	539.85	-177.35	-32.43
1.916	-161.68	-189.01	527.83	-170.56	-29.8
1.913	-163.31	-190.57	532.74	-172.08	-29.74
1.91	-165.34	-192.55	539.03	-174.16	-29.7
1.906	-167.99	-195.15	547.41	-177.22	-29.8
1.901	-171.25	-198.32	557.85	-181.46	-30.19
1.894	-174.61	-201.58	568.81	-186.39	-30.93
1.887	-177.77	-204.64	579.29	-191.74	-32.16
1.88	-180.68	-207.42	589	-197.52	-34.04
1.873	-183.46	-210.03	598.29	-203.95	-36.7

**Table3(S)** (continued)					
Cy					
Displacement of C-P bond	ΔE(els)	ΔE(c)	ΔE(rep)	ΔE(orb)	ΔE(int)
1.914	-182.4	-209.95	588.54	-197.15	-39.2
1.912	-182.39	-209.93	588.41	-196.78	-38.97
1.91	-182.53	-210.08	588.79	-196.56	-38.72
1.9087	-182.89	-210.46	589.89	-196.58	-38.42
1.908	-183.58	-211.19	592.16	-197.02	-38.1
1.9058	-184.96	-212.62	596.75	-198.49	-37.89
1.9017	-187.35	-215.04	604.82	-201.61	-37.88
1.8958	-190.24	-217.94	614.7	-205.84	-38.14
1.889	-193.05	-220.75	624.47	-210.59	-38.85
1.883	-195.69	-223.35	633.73	-215.89	-40.24
1.877	-198.18	-225.75	642.36	-221.73	-42.45

**Table3(S)** (continued)					
Furyl					
Displacement of C-P bond	ΔE(els)	ΔE(c)	ΔE(rep)	ΔE(orb)	ΔE(int)
1.959	-139.62	-164.51	457.86	-136.01	-12.38
1.954	-141.76	-166.65	464.46	-138.38	-12.58
1.947	-144.29	-169.16	472.29	-141.22	-12.84
1.939	-147.19	-172.04	481.27	-144.49	-13.12
1.931	-150.45	-175.26	491.4	-148.25	-13.47
1.921	-154.08	-178.84	502.76	-152.63	-13.94
1.911	-157.96	-182.67	515.07	-157.73	-14.67
1.902	-161.71	-186.36	527.13	-163.25	-15.76
1.893	-165.14	-189.74	538.38	-169.08	-17.3
1.886	-168.25	-192.8	548.76	-175.31	-19.48
1.878	-171.29	-195.74	558.89	-182.2	-22.36

**Table3(S)** (continued)					
H					
Displacement of C-P bond	ΔE(els)	ΔE(c)	ΔE(rep)	ΔE(orb)	ΔE(int)
1.924	-159.49	-186.79	522.4	-165.21	-24.01
1.923	-160.05	-187.31	523.93	-165.47	-23.86
1.922	-160.93	-188.14	526.43	-166.06	-23.73
1.919	-162.21	-189.37	530.2	-167.11	-23.63
1.915	-163.98	-191.09	535.51	-168.76	-23.57
1.91	-166.33	-193.39	542.78	-171.26	-23.64
1.904	-169.23	-196.25	551.96	-174.85	-23.96
1.897	-172.23	-199.24	561.77	-179.21	-24.65
1.891	-175.04	-202.04	571.15	-184.05	-25.84
1.885	-177.57	-204.57	579.8	-189.36	-27.68
1.879	-180.06	-207.02	588.32	-195.43	-30.3

**Table3(S)** (continued)					
Thiophen					
Displacement of C-P bond	ΔE(els)	ΔE(c)	ΔE(rep)	ΔE(orb)	ΔE(int)
1.942	-147.52	-173.14	483.62	-147.3	-18.03
1.94	-148.55	-174.15	486.67	-148.18	-18
1.936	-150.01	-175.59	491.06	-149.56	-18.01
1.932	-151.88	-177.44	496.76	-151.43	-18.06
1.926	-154.18	-179.71	503.84	-153.85	-18.17
1.919	-157.01	-182.51	512.65	-157.1	-18.41
1.911	-160.29	-185.76	523.07	-161.3	-18.92
1.903	-163.56	-189.01	533.69	-166.08	-19.75
1.895	-166.51	-191.96	543.51	-171.14	-21.03
1.889	-169.15	-194.62	552.52	-176.67	-22.95
1.882	-171.65	-197.1	561.11	-182.82	-25.63

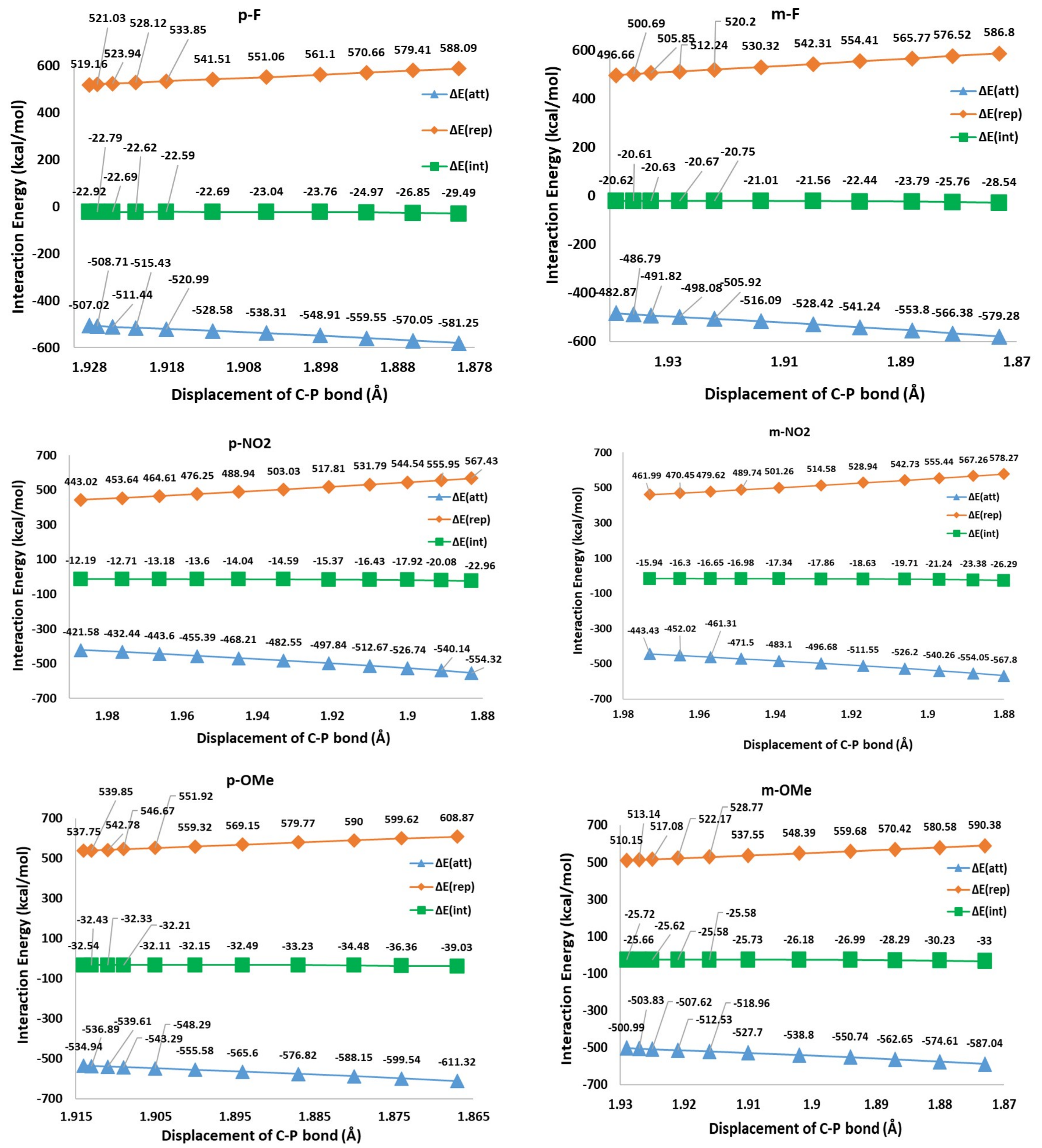

Figure 3(S) The EDA analyses for other derivatives in the step2(b)

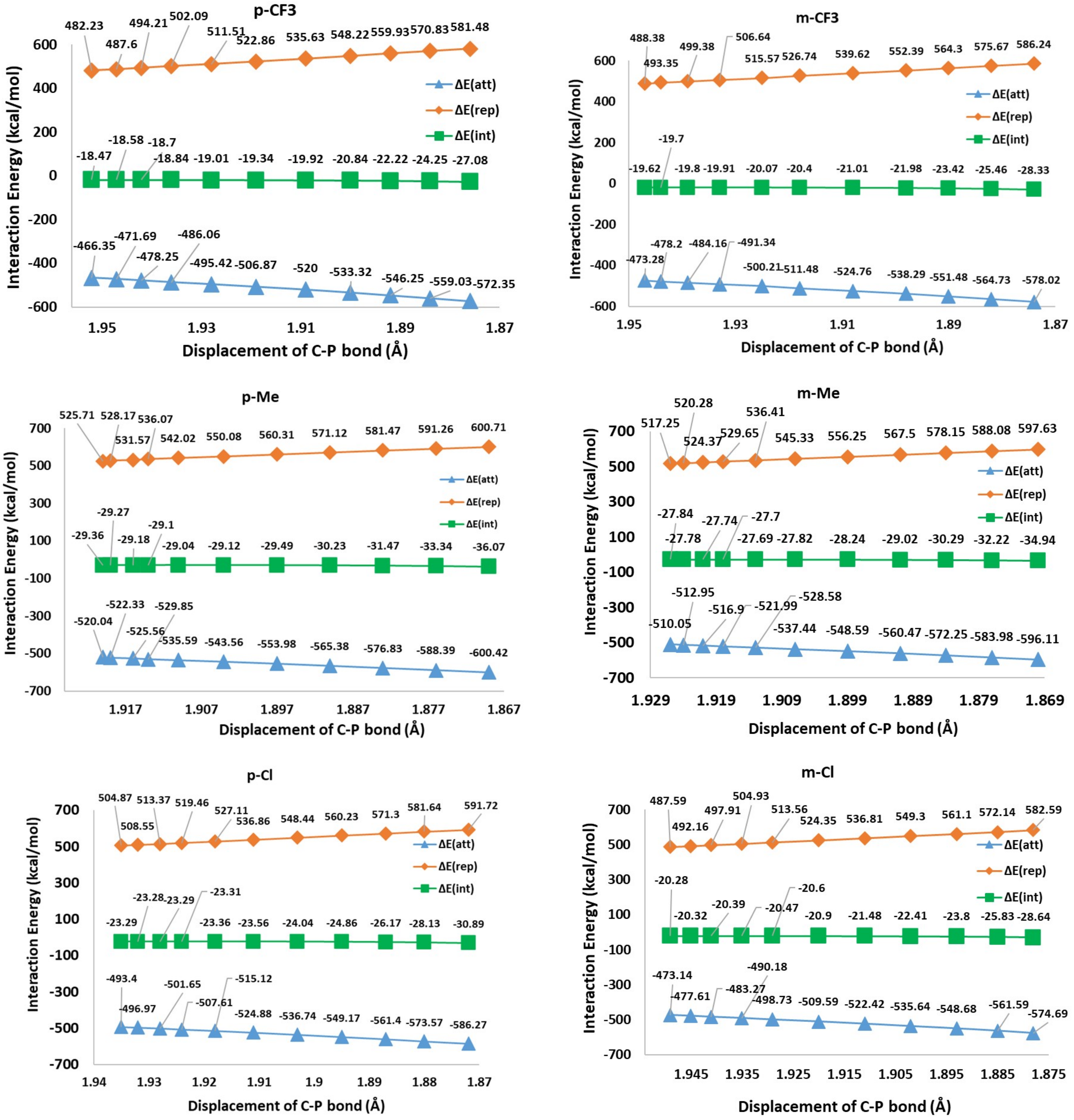

Figure 3(S). Countinued

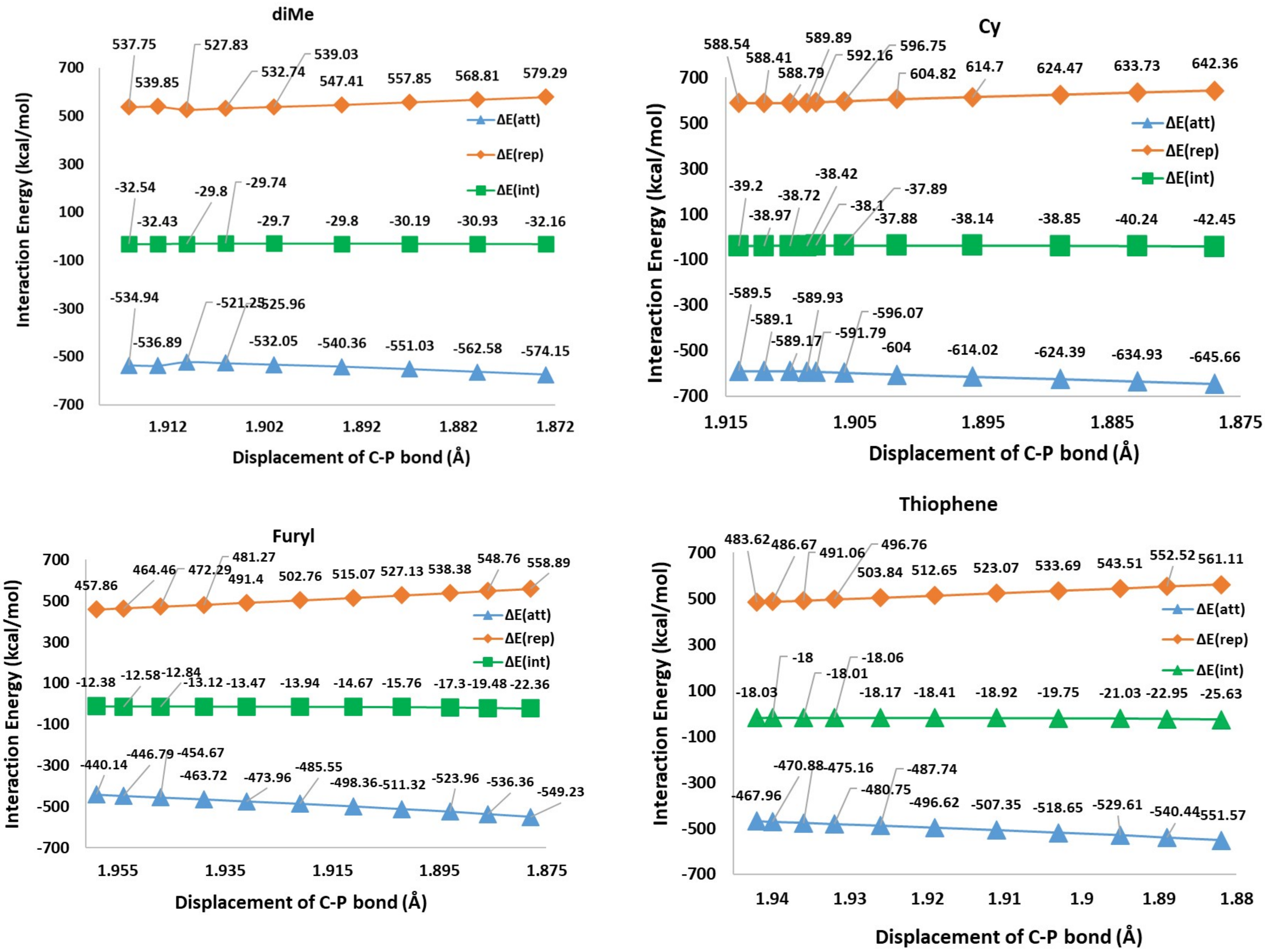

Figure 3(S). Countinued

**p-NO$_2$**	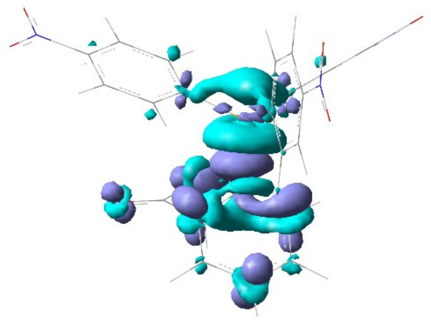<br>$\Delta\rho^{Orb} = -203.36$	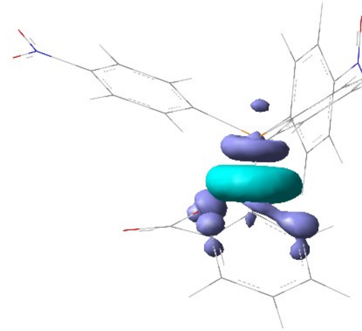<br>$\Delta\rho^{Pauli} = 307.00$	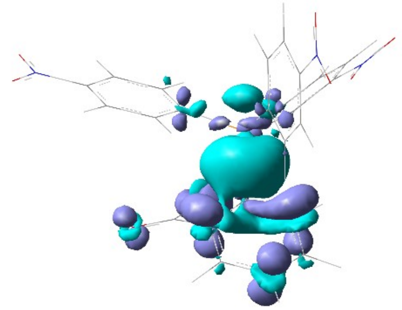<br>$\Delta\rho^{Total} = 103.64$
**m-NO$_2$**	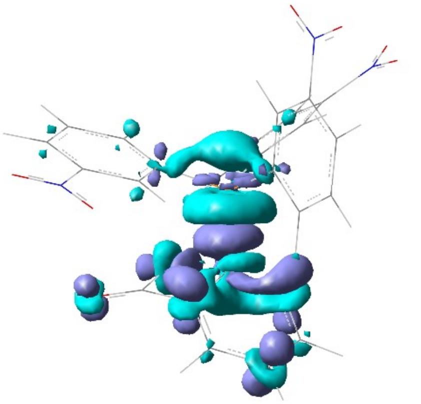<br>$\Delta\rho^{Orb} = -208.39$	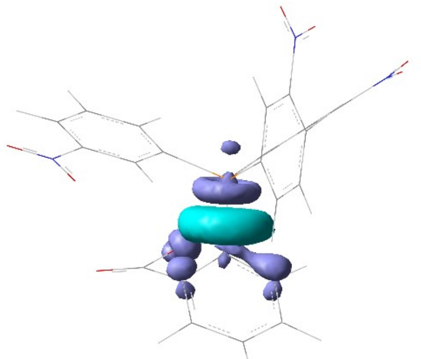<br>$\Delta\rho^{Pauli} = 312.85$	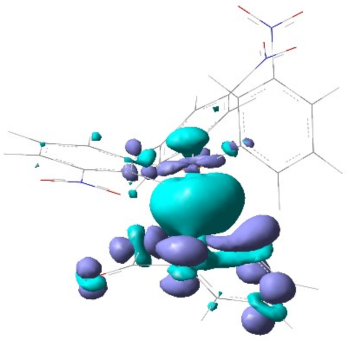<br>$\Delta\rho^{Total} = 104.46$
**p-CF$_3$**	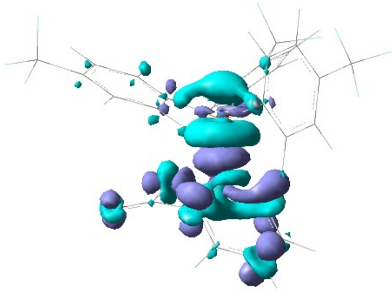<br>$\Delta\rho^{Orb} = -213.07$	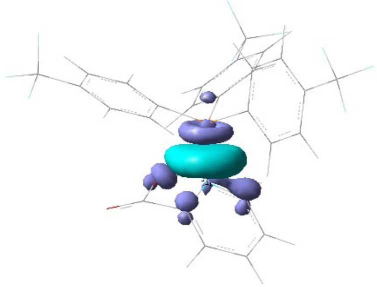<br>$\Delta\rho^{Pauli} = 318.42$	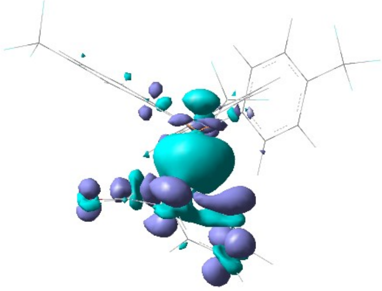<br>$\Delta\rho^{Total} = 105.35$
**m-CF$_3$**	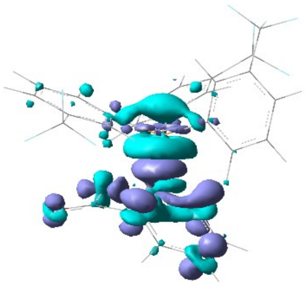<br>$\Delta\rho^{Orb} = -214.89$	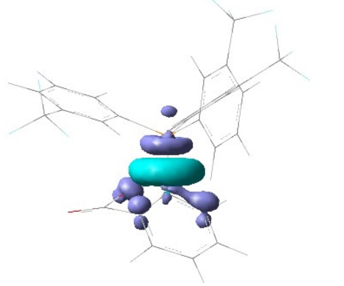<br>$\Delta\rho^{Pauli} = 320.76$	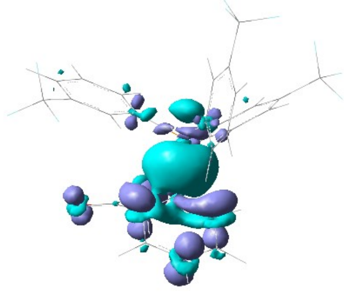<br>$\Delta\rho^{Total} = 105.87$

Figure 4(S). The deformation density components for the derivatives in **TS2(b)**

**p-F**	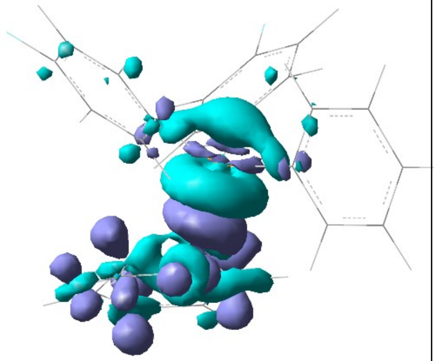 $\Delta\rho^{Orb} = -217.91$	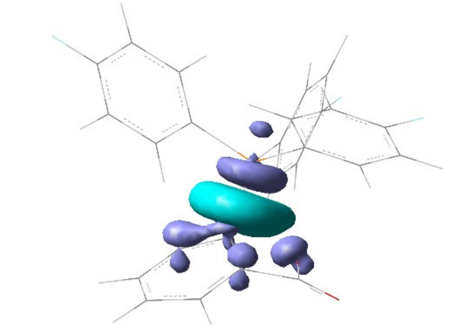 $\Delta\rho^{Pauli} = 323.16$	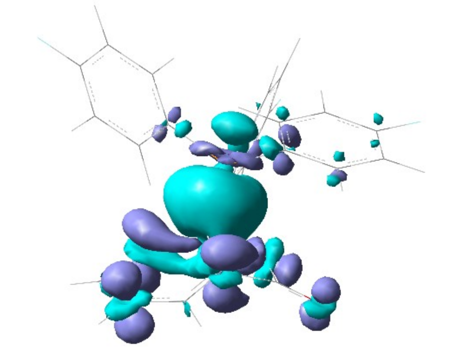 $\Delta\rho^{Total} = 105.25$
**m-F**	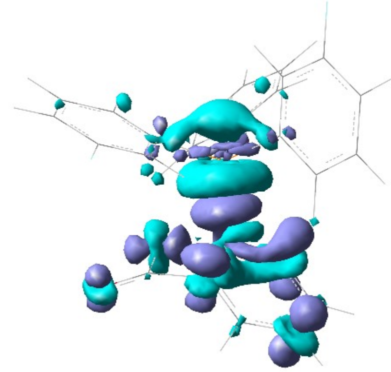 $\Delta\rho^{Orb} = -217.51$	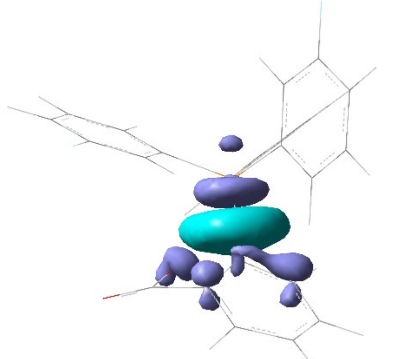 $\Delta\rho^{Pauli} = 323.60$	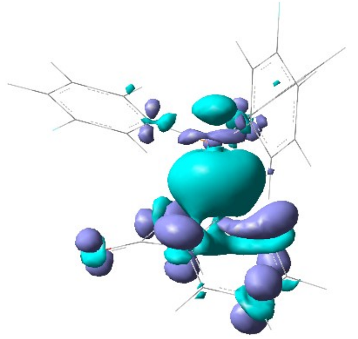 $\Delta\rho^{Total} = 106.09$
**p-Cl**	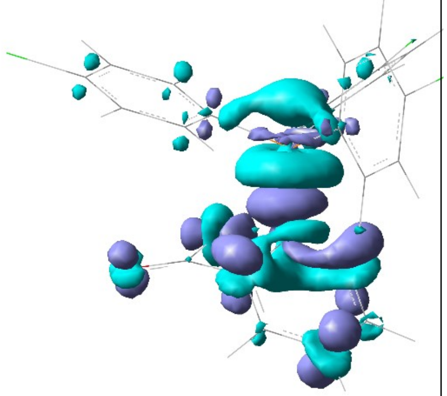 $\Delta\rho^{Orb} = -220.30$	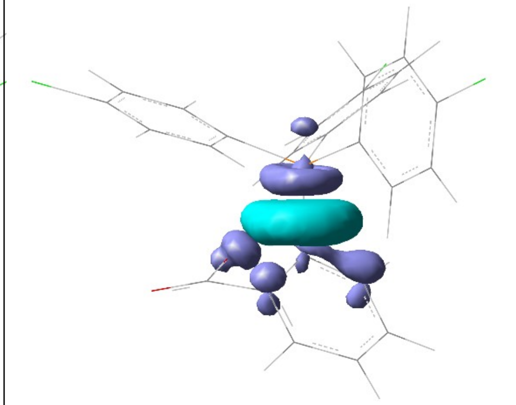 $\Delta\rho^{Pauli} = 326.85$	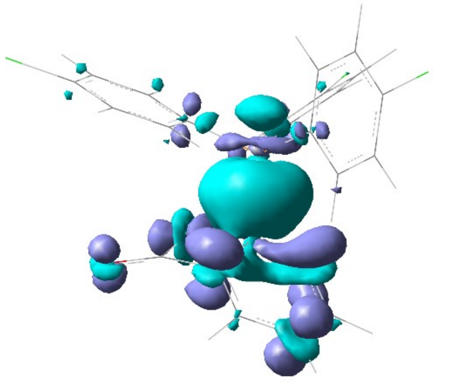 $\Delta\rho^{Total} = 106.55$
**m-Cl**	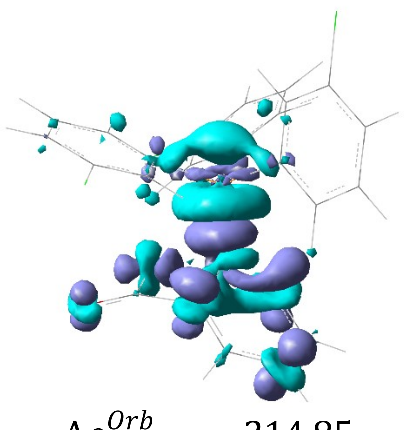 $\Delta\rho^{Orb} = -214.85$	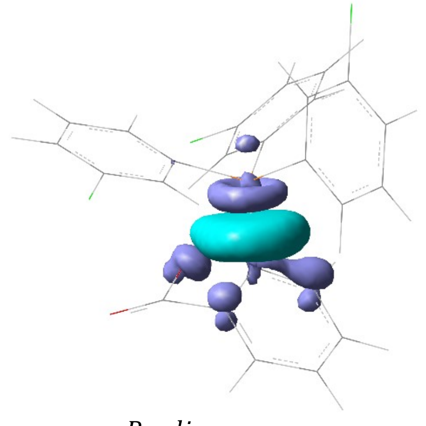 $\Delta\rho^{Pauli} = 320.11$	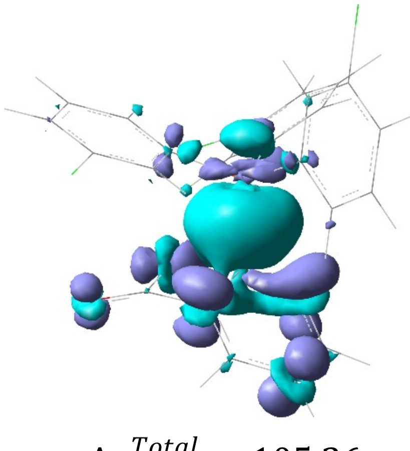 $\Delta\rho^{Total} = 105.26$

Figure 4(S). Countinued

**p-Me**	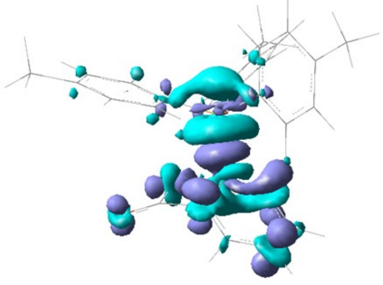 $\Delta\rho^{Orb} = -228.7$	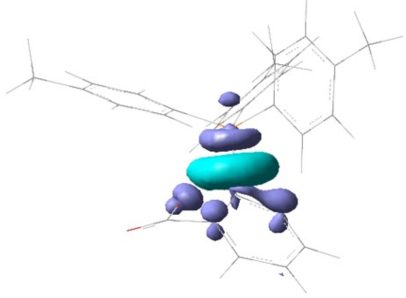 $\Delta\rho^{Pauli} = 336.44$	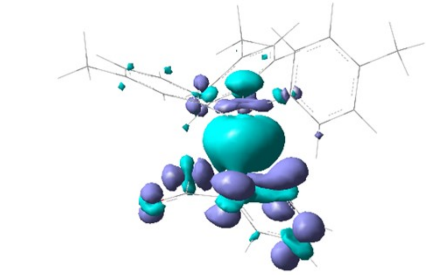 $\Delta\rho^{Total} = 107.74$
**m-Me**	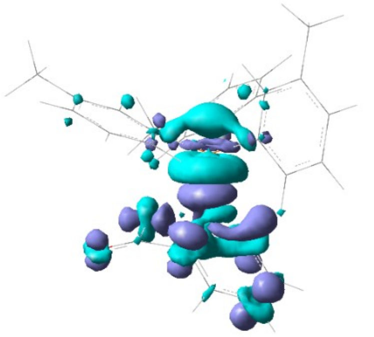 $\Delta\rho^{Orb} = -226.74$	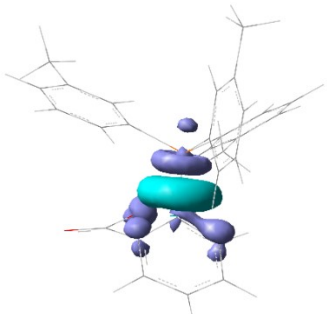 $\Delta\rho^{Pauli} = 334.10$	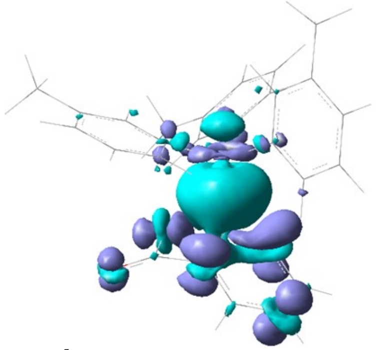 $\Delta\rho^{Total} = 107.36$
**p-OMe**	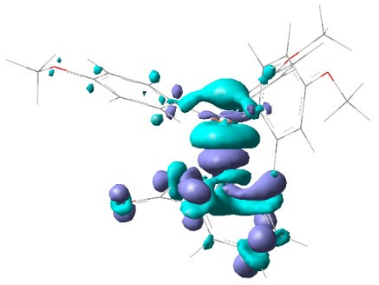 $\Delta\rho^{Orb} = -233.45$	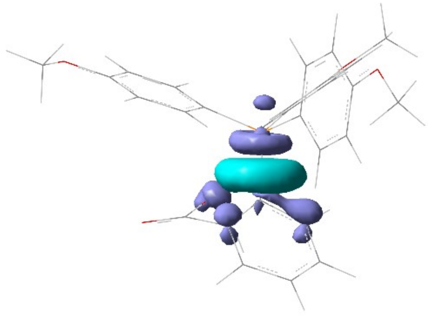 $\Delta\rho^{Pauli} = 342.01$	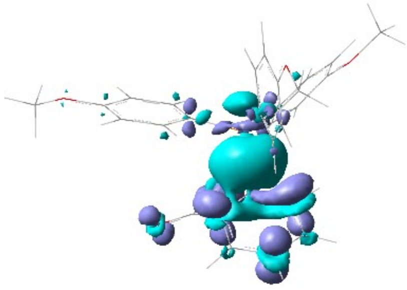 $\Delta\rho^{Total} = 108.56$
**m-OMe**	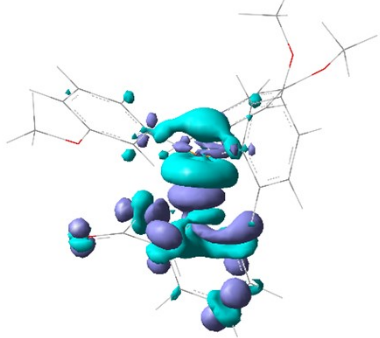 $\Delta\rho^{Orb} = -222.61$	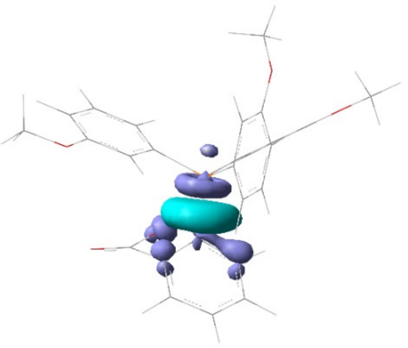 $\Delta\rho^{Pauli} = 329.27$	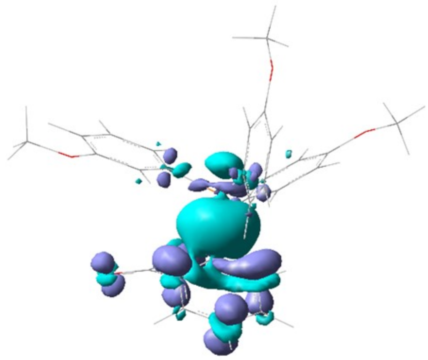 $\Delta\rho^{Total} = 106.66$

Figure 4(S). Countinued

**Furyl**	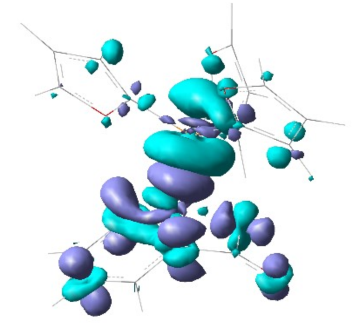 $\Delta\rho^{Orb} = -205.18$	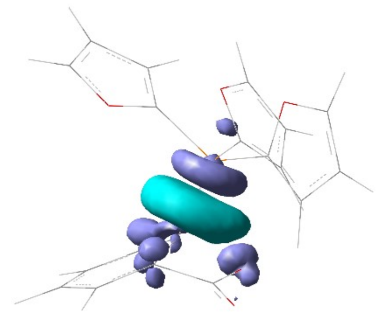 $\Delta\rho^{Pauli} = 310.04$	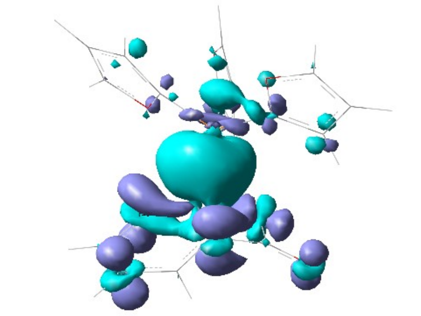 $\Delta\rho^{Total} = 104.86$
**diMe**	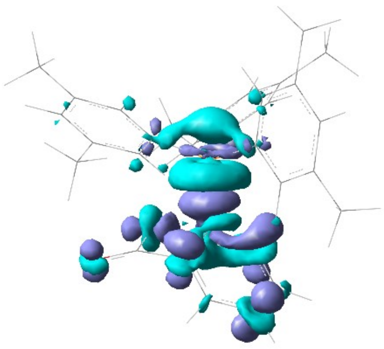 $\Delta\rho^{Orb} = -227.48$	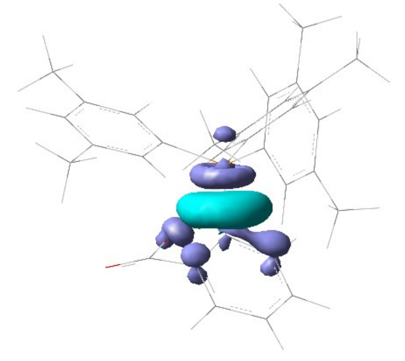 $\Delta\rho^{Pauli} = 334.40$	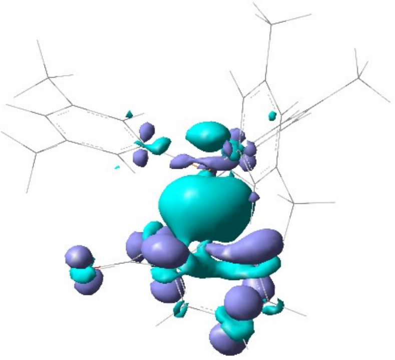 $\Delta\rho^{Total} = 106.92$
**Cy**	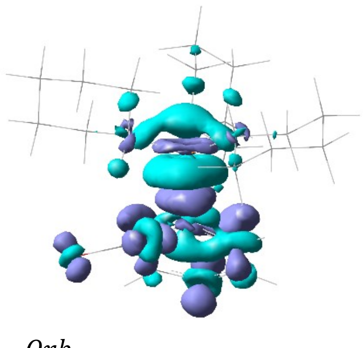 $\Delta\rho^{Orb} = -253.83$	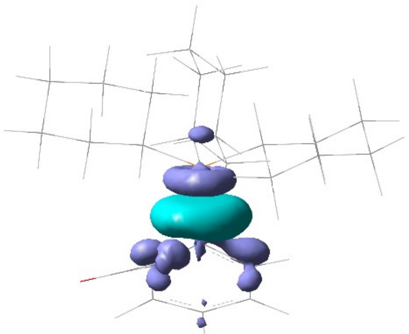 $\Delta\rho^{Pauli} = 360.80$	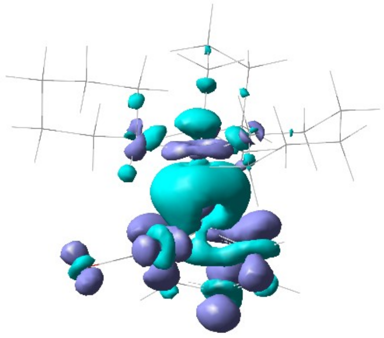 $\Delta\rho^{Total} = 106.97$
**Thiopene**	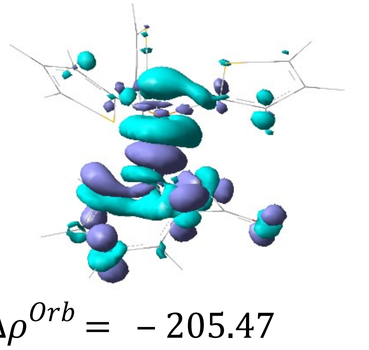 $\Delta\rho^{Orb} = -205.47$	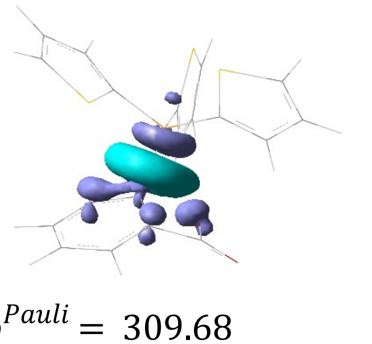 $\Delta\rho^{Pauli} = 309.68$	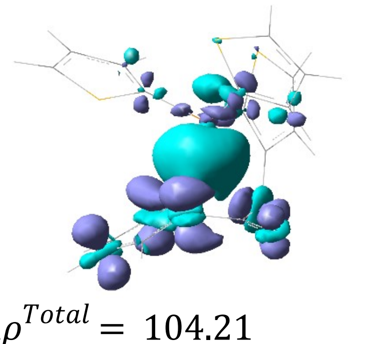 $\Delta\rho^{Total} = 104.21$

Figure 4(S). Countinued
