# Supplementary material for: Exploring CO2 activation mechanisms with triphenylphosphine derivatives: insights from energy decomposition and deformation density analyses
Source: RSC Adv. 2025 Apr 22;15(17):12917–30. doi: 10.1039/d5ra00804b (PMC12013607; doi:10.1039/d5ra00804b)
Supplement: RA-015-D5RA00804B-s002 [file RA-015-D5RA00804B-s002.pdf]

Supplementary Information (SI) for RSC Advances.
This journal is © The Royal Society of Chemistry 2025

Supporting Information 2

Exploring CO_2 Activation Mechanisms with Triphenylphosphine Derivatives: Insights from Energy Decomposition and Deformation Density Analyses

Hossein Sabet-Sarvestani*[a], Shadi Bolourian [a], Fereshteh Hosseini[a], Mohammad Javad Seddighi[b], Hamed Hosseini[a], and Hossein Eshghi [b]

[a] Department of Food Additives, Food Science and Technology Research Institute, Research Center for Iranian Academic Center for Education, Culture and Research (ACECR), Khorasan Razavi Branch, Mashhad, IRAN

[b] Department of Chemistry, Faculty of Science, Ferdowsi University of Mashhad, Mashhad, IRAN

** Correspondence to: Hossein Sabet-Sarvestani. TeleFax: ++989371411532 (E-mail address: bozorgmehr1388@gmail.com)*

Cartesian coordinate of the involved species in M062X/def2svp

Benzyne:

0 1

C	-0.70557600	1.05327900	0.00000200
C	0.70305900	1.05475200	0.00000100
C	1.46553800	-0.13068900	0.00000000
C	0.62584200	-1.23556200	-0.00000100
C	-0.62235800	-1.23531300	-0.00000200
C	-1.46571300	-0.13378200	-0.00000100
H	-1.23245600	2.01039400	0.00000300
H	2.55547800	-0.12676900	0.00000000
H	-2.55560000	-0.13272500	-0.00000100
H	1.22783100	2.01299200	0.00000300

CO_2:

0 1

C	-4.04783400	0.67837100	0.12208900
O	-3.50907100	-0.26771600	-0.26684300
O	-4.58660300	1.62445400	0.51102100

In1(b):

0 1

C	-0.50640600	0.14914600	0.32753300
C	0.55841000	0.33606000	-0.56009300
C	1.55890300	-0.63523500	-0.78811800
C	1.37337500	-1.77699200	-0.04896300
C	0.32440800	-1.98268200	0.83493600
C	-0.65640500	-1.04074300	1.06583900
H	-1.23789000	0.94821200	0.44835600
H	2.38153700	-0.47505800	-1.48240400
H	-1.48459500	-1.18743100	1.75855200
H	0.62135000	1.27982200	-1.10459900
C	0.96534700	-3.32327100	1.07008700
O	0.85212700	-4.32269000	1.68057000
O	1.99809400	-3.01381200	0.14387100

TS1(b): (ν = 609.43i(cm^{-1}))

0 1

C	2.41771900	0.63962400	0.00327800
C	2.28633100	-0.75667800	0.00378100
C	1.01407400	-1.33898800	0.00015400
C	0.02510800	-0.38184000	-0.00487000
C	-0.02507500	0.95752000	-0.00766400
C	1.29324100	1.47521800	-0.00178700
H	3.41867200	1.07619700	0.00735200
H	0.84376000	-2.41463000	0.00145000
H	1.46280800	2.55777300	-0.00153200
H	3.16896400	-1.39768200	0.00754400
C	-2.27198800	-0.07191900	0.00104700
O	-3.16435900	0.62982300	0.00663900
O	-1.50197500	-0.99973400	-0.00394600

diMe:

$PPhe_3$:

0 1

P	-0.00165500	0.04015100	-1.48383500
C	1.53085200	-0.57803700	-0.66226200
C	2.75263200	-0.25092300	-1.26778800
C	1.53165400	-1.31080200	0.52592500
C	3.96672100	-0.62939900	-0.69208600
H	2.75685000	0.31516400	-2.20397800
C	2.73587500	-1.71324900	1.12129300
H	0.58502400	-1.57064400	1.00771500
C	3.93721900	-1.36123600	0.50347200
H	4.88175300	-1.66808300	0.96216400
C	-1.28531400	-1.02079700	-0.68737600
C	-2.08718400	-0.61951100	0.38088100
C	-1.44238300	-2.31220700	-1.21586700
C	-3.04073800	-1.49126300	0.93129800
H	-1.97558600	0.38247500	0.80383300
C	-2.37052200	-3.20340200	-0.67898700
H	-0.82359000	-2.62847400	-2.06141800
C	-3.16475600	-2.77265200	0.39487500
H	-3.90223800	-3.46012500	0.81926900
C	-0.25689200	1.65063700	-0.61961000
C	-1.30236800	2.46383800	-1.08578500
C	0.53060600	2.09705400	0.44166100
C	-1.58044300	3.69358200	-0.48937500
H	-1.91603000	2.12814300	-1.92756400
C	0.28516800	3.33884800	1.04850700
H	1.34974100	1.47726400	0.81436900
C	-0.77230800	4.11606500	0.57722600
H	-0.97476800	5.08263500	1.04765100
C	-3.90291100	-1.03614200	2.08036800
H	-3.28496300	-0.71066300	2.92993600
H	-4.52641700	-0.17895800	1.78587100
H	-4.56599800	-1.83974000	2.42536300
C	-2.53822600	-4.59264300	-1.23760400
H	-2.40064100	-5.35055800	-0.45273600
H	-3.54938200	-4.73021900	-1.64856100
H	-1.81370400	-4.79022200	-2.03784000
C	1.16289300	3.81528500	2.17687000
H	2.15941700	4.09547300	1.80291200
H	0.73042600	4.69208500	2.67597000
H	1.30734200	3.02431200	2.92633000
C	-2.71246300	4.56136000	-0.97457900
H	-3.45450100	4.71971600	-0.17802700

H	-2.34540700	5.55244300	-1.27889700
H	-3.22365700	4.10565200	-1.83227800
C	5.28268400	-0.27174200	-1.33226200
H	5.88979100	0.35143600	-0.65924600
H	5.86900300	-1.17495300	-1.55605700
H	5.13274600	0.28076200	-2.26853400
C	2.71870800	-2.51788300	2.39538900
H	2.05113000	-2.06233700	3.14039900
H	2.34760600	-3.53677500	2.20718600
H	3.72267800	-2.59850900	2.83154800

In1(a):

0 1

P	-1.00930400	-0.14603100	-1.52940500
C	0.67675400	-0.72117600	-1.20244500
C	1.67802500	-0.43355800	-2.13329300
C	0.98301200	-1.36346300	-0.00009300
C	3.00561800	-0.78083400	-1.86493100
H	1.42131900	0.05377400	-3.07722100
C	2.30294700	-1.72942800	0.28314000
H	0.19382200	-1.59041100	0.72105400
C	3.29386000	-1.42570700	-0.65684300
H	4.32903400	-1.70692600	-0.44290000
C	-2.15429300	-1.19250400	-0.57814300
C	-2.53086800	-0.80203300	0.71354400
C	-2.60411900	-2.40258500	-1.11382800
C	-3.36786900	-1.61806100	1.47963000
H	-2.17576400	0.14154300	1.13408100
C	-3.45363900	-3.22546100	-0.36541400
H	-2.27736400	-2.68341400	-2.11983000
C	-3.81966700	-2.81923600	0.92176200
H	-4.47880900	-3.46078600	1.51406000
C	-1.15474700	1.52071800	-0.82105300
C	-2.41381000	2.14051700	-0.84336700
C	-0.05236700	2.18479100	-0.28730200
C	-2.57516300	3.42660100	-0.33143700
H	-3.27879900	1.61500000	-1.25831900
C	-0.19169800	3.48141200	0.23269900
H	0.92666600	1.70022700	-0.26511900
C	-1.45151300	4.07758500	0.20225200
H	-1.57076900	5.08559600	0.60949300
C	-3.76236100	-1.22685500	2.87983400
H	-3.20319100	-1.81900400	3.62005200
H	-3.55510100	-0.16614300	3.07047200
H	-4.83143400	-1.41053500	3.05394800
C	-3.98317600	-4.50957200	-0.94783800
H	-4.12861600	-5.26987400	-0.16888400
H	-4.95755300	-4.34069500	-1.43170800
H	-3.30034400	-4.91002800	-1.70814600
C	1.00526900	4.20286200	0.79290600
H	1.67595800	4.52565500	-0.01761500
H	0.70534300	5.09342600	1.35937900
H	1.58612800	3.54639400	1.45542700
C	-3.91817900	4.10663200	-0.33094700
H	-4.28852100	4.23338300	0.69719300
H	-3.84882600	5.10802300	-0.77865300
H	-4.66025200	3.52539000	-0.89225000
C	4.10106000	-0.44746800	-2.84294300
H	4.51650400	0.55010800	-2.63246900
H	4.92464500	-1.17028500	-2.77698600
H	3.72326200	-0.43793900	-3.87360000
C	2.64864400	-2.45899300	1.55436500
H	1.86635500	-2.32980700	2.31327600
H	2.75687700	-3.53755900	1.36348200
H	3.60126700	-2.10103400	1.96781700
C	-1.72415400	-0.23718200	-5.99075800
C	-1.84180400	0.99898300	-5.34144400
C	-1.64210600	1.05527500	-3.96712300
C	-1.32824200	-0.14350600	-3.29790900
C	-1.17471600	-1.42177800	-3.87497900
C	-1.40706200	-1.39339000	-5.27333200
H	-1.87932000	-0.28788500	-7.07259100
H	-1.72236700	2.00848300	-3.43835600
H	-1.33113100	-2.32363300	-5.85281200
H	-2.08336700	1.90357200	-5.90231300

Product:

0 1

C	-2.99865900	-0.01688300	-3.91320300
C	-1.71537800	-0.22053000	-4.42424700
C	-0.60896000	-0.26340600	-3.57431800
C	-0.79371000	-0.09408500	-2.19695300
C	-2.08365400	0.09464500	-1.69056300
C	-3.18400300	0.13682500	-2.54039200
H	-3.85443600	0.01442800	-4.58914200
H	0.38273000	-0.43030900	-3.99351400
H	-4.17168800	0.28432100	-2.10097600
H	-1.56958600	-0.35052300	-5.49730100
C	-2.21775000	0.22027100	-0.18757200
O	-3.31473500	0.35990600	0.33340500
O	-1.08120100	0.16273100	0.38945400
P	0.56995400	-0.18412600	-0.97518300
C	1.16673200	1.35811400	-0.20073300
C	2.45850700	1.39230900	0.32509500
C	0.37720700	2.51093200	-0.21608700
C	2.96462200	2.57494900	0.87983600
H	3.09358900	0.50283700	0.29458400
C	0.87714800	3.71110200	0.29210500
H	-0.63109100	2.47892200	-0.63250100
C	2.16456800	3.71932200	0.84435700
H	2.55795900	4.65405100	1.25442600
C	2.02406500	-0.44382600	-2.11797600

C	2.67460800	-1.66760900	-2.26291600
C	2.46019100	0.65388900	-2.88105500
C	3.74682600	-1.81560300	-3.16049900
H	2.36580300	-2.53396300	-1.67472200
C	3.51341500	0.53470400	-3.78444500
H	1.96827800	1.62535300	-2.77048500
C	4.14597000	-0.71290400	-3.91106000
H	4.97666500	-0.81755100	-4.61475200
C	0.60982100	-1.72410800	-0.01128900
C	0.04682900	-2.87356800	-0.56959300
C	1.21811400	-1.76424700	1.24239900
C	0.09477000	-4.08658400	0.12583800
H	-0.42687300	-2.83768700	-1.55466600
C	1.28049700	-2.96892600	1.95179900
H	1.63632300	-0.85757200	1.68514400
C	0.72228600	-4.11478400	1.37565400
H	0.77522500	-5.06149200	1.92052100
C	4.44570900	-3.14435200	-3.28331800
H	4.98762300	-3.38622700	-2.35676600
H	3.72275800	-3.95384800	-3.45801400
H	5.16832600	-3.14006800	-4.10910600
C	3.97641300	1.70653100	-4.60948100
H	3.86440200	1.49725800	-5.68343600
H	3.40245000	2.61171100	-4.37391700
H	5.04074800	1.91556100	-4.42832000
C	1.90770400	-3.02352000	3.31975800
H	1.14349300	-2.88110000	4.09920300
H	2.38399700	-3.99680000	3.49797300
H	2.66121400	-2.23519100	3.44524300
C	-0.53325800	-5.32901200	-0.44866400
H	0.05532900	-6.22189500	-0.19865800
H	-1.54379600	-5.47412300	-0.03692500
H	-0.62448300	-5.26207900	-1.54050500
C	4.33317100	2.60464400	1.50734300
H	4.79743100	3.59374100	1.39862200
H	4.26910100	2.38497400	2.58416500
H	4.99522600	1.85471600	1.05479800
C	0.05600600	4.97341100	0.26135500
H	-0.09566600	5.36416800	1.27801500
H	0.56702200	5.75755200	-0.31611400
H	-0.92859300	4.79888000	-0.19020900

TS2(b): (ν = 485.39i(cm^{-1}))

0 1

C	-2.54652500	0.33114300	3.64833600
C	-1.42171400	1.17307100	3.57118100
C	-0.30753000	0.91573400	2.77651700
C	-0.34161600	-0.23525700	1.92097800
C	-1.39974000	-1.20113500	2.20027700
C	-2.47343400	-0.92497700	3.02704600
H	-3.38997100	0.59574900	4.28471700
H	0.52758900	1.61825900	2.74569800
H	-3.18984700	-1.71490000	3.26876300
H	-1.42206000	2.09674500	4.15642100
C	-0.48275500	-2.34867100	2.06800200
O	-0.57957400	-3.54949000	2.04219800
O	0.67023100	-1.64175600	2.00299600
P	0.12971600	0.11031700	0.14076400
C	1.74698200	0.91325000	0.14643500
C	2.81562400	0.23042700	0.75243300
C	1.92592300	2.19725700	-0.37047700
C	4.07593700	0.82363100	0.81229900
H	2.65376800	-0.75851400	1.18819600
C	3.18465700	2.81145600	-0.30942900
H	1.09065800	2.73662600	-0.82122800
C	4.23917000	2.10891800	0.27414300
H	5.22610900	2.57786400	0.32100300
C	-1.12710200	1.18457700	-0.58865400
C	-2.26626000	1.54308100	0.13720300
C	-0.98685600	1.56836500	-1.92950900
C	-3.26901200	2.31022000	-0.46853800
H	-2.39454900	1.22682400	1.17336900
C	-1.97126200	2.34176900	-2.54687300
H	-0.10983400	1.25928400	-2.50558700
C	-3.10055200	2.70104700	-1.79893300
H	-3.88122200	3.29931900	-2.27803300
C	0.13161100	-1.39918000	-0.85007800
C	1.31300300	-1.97608000	-1.33033900
C	-1.09939200	-2.03314200	-1.03571900
C	1.26926900	-3.20065600	-1.99666000
H	2.27235400	-1.47409600	-1.19101100
C	-1.15819000	-3.27905800	-1.67232000
H	-2.02130100	-1.57113800	-0.67191100
C	0.02924900	-3.83894400	-2.14576200
H	-0.00832500	-4.80648600	-2.65413200
C	-4.50006800	2.70882900	0.30183600
H	-5.41234600	2.43547100	-0.24686800
H	-4.52456000	2.22231300	1.28542800
H	-4.52496100	3.79743900	0.45763400
C	-1.83268000	2.78351500	-3.97951900
H	-1.71804800	3.87594800	-4.03850600
H	-0.95926500	2.32175400	-4.45678500
H	-2.72739200	2.51685200	-4.55955500
C	-2.47248300	-4.00094700	-1.79917800
H	-2.41356000	-4.81308900	-2.53452400
H	-2.75463500	-4.43915500	-0.82948400
H	-3.27621400	-3.31349900	-2.09592700
C	2.51926000	-3.83489900	-2.54647700
H	3.41072800	-3.25010900	-2.28720300
H	2.64535900	-4.85263000	-2.15039400
H	2.46620500	-3.91471400	-3.64220200
C	5.24415400	0.10905000	1.43774500
H	6.03198100	-0.07054200	0.69153900
H	5.68742900	0.71440000	2.24117800
H	4.94065000	-0.85761900	1.85820300

C	3.37406500	4.20662300	-0.84356300
H	2.85156200	4.33806300	-1.80077000
H	2.96487500	4.94769600	-0.14035800
H	4.43738200	4.43345600	-0.99158900

CF_3:

Meta-$PPhe_3$:

0 1

P	0.23552000	0.01404300	-1.28861000
C	-0.48064400	1.42258100	-0.33448700
C	-1.62540500	2.02112600	-0.87966100
C	0.04786500	1.92939100	0.85685700
C	-2.23234300	3.08928100	-0.22799800
H	-2.04569100	1.65240400	-1.81834400
C	-0.55801200	3.01397600	1.49731300
H	0.93995600	1.47894500	1.29632400
C	-1.70099900	3.59656600	0.96087100
H	-0.13305900	3.40281400	2.42337700
C	1.77833500	-0.32833600	-0.33729300
C	1.88602100	-1.27926500	0.68231400
C	2.91036400	0.41867800	-0.69395700
C	3.10080900	-1.47639200	1.34407300
H	1.01803200	-1.87641400	0.96870700
C	4.11292100	0.22580900	-0.02237900
C	4.21756500	-0.72314900	0.99822000
H	3.17279600	-2.22277400	2.13583600
C	-0.85036600	-1.37259000	-0.73787300
C	-0.79490600	-2.55858400	-1.48404700
C	-1.71168900	-1.30225600	0.36079500
C	-1.56918700	-3.66162600	-1.13022900
H	-0.13589900	-2.61809500	-2.35391600
C	-2.49493400	-2.40616200	0.70100600
H	-1.77128300	-0.39357400	0.96405700
C	-2.42728500	-3.58854700	-0.03426500
H	-1.51148000	-4.57961500	-1.71591500
H	-3.04122600	-4.44409700	0.24925100
H	-2.17704600	4.44425400	1.45679700
H	5.16882200	-0.87380300	1.51196500
C	-3.48448200	3.71183800	-0.78195300
C	-3.45493800	-2.29530700	1.85390800
H	2.85250200	1.15416700	-1.49942300
C	5.33504600	1.02286600	-0.38863800
F	-3.39597000	5.04502300	-0.81492500
F	-4.55113500	3.41899300	-0.02936700
F	-3.75074900	3.29719800	-2.02097200
F	-2.96902100	-1.52497100	2.83076600
F	-4.61828800	-1.75193100	1.47858800
F	-3.73711500	-3.48700600	2.38395300
F	5.78622700	1.73257400	0.65053100
F	6.34010900	0.22855900	-0.77278600
F	5.10280400	1.87949400	-1.38359800

Meta-In1(a):

0 1

P	-0.16331500	-0.04865900	0.21101900
C	0.52036800	1.09929200	-1.01448000
C	1.27335900	2.18665500	-0.56667100
C	0.36964700	0.85741200	-2.38405000
C	1.87971200	3.02230000	-1.50239900
H	1.38190400	2.38001500	0.50320500
C	0.97811600	1.70420000	-3.30857000
H	-0.22566900	0.01142100	-2.73365100
C	1.73758800	2.78684200	-2.86979200
H	0.86009500	1.51793000	-4.37596900
C	-1.63853800	-0.82602100	-0.51927000
C	-1.52438700	-2.03186500	-1.22630700
C	-2.87776800	-0.19096100	-0.40729300
C	-2.65117500	-2.60296500	-1.81295900
H	-0.55875500	-2.53219200	-1.32168900
C	-3.99629100	-0.78233800	-0.99311500
C	-3.89341700	-1.98107400	-1.69568200
H	-2.56035300	-3.54061800	-2.36135000
C	1.05351700	-1.38361400	0.41651900
C	0.71690500	-2.47773600	1.22970000
C	2.31275600	-1.31824700	-0.17893500
C	1.63689200	-3.49937300	1.43869300
H	-0.26966900	-2.53499700	1.69670000
C	3.22782900	-2.34960400	0.04097500
H	2.58741700	-0.47743300	-0.81949900
C	2.89800100	-3.43830000	0.84298300
H	1.37149600	-4.35047700	2.06582800
H	3.62192500	-4.23880200	0.99737900
H	2.22107000	3.44956900	-3.58777800
H	-4.77969600	-2.42555200	-2.14891800
H	-2.93454000	0.75892900	0.13846900
C	-0.10956400	1.20835200	4.07291500
C	-1.02594200	2.26559600	3.97318400
C	-1.64576500	2.56724300	2.75882700
C	-1.40394500	1.86021100	1.55418200
C	-0.48619700	0.80948100	1.74635100
C	0.17290300	0.45662600	2.94002300
H	0.37697200	0.98051200	5.02261200
H	-2.34895300	3.41010700	2.76038100
H	0.88777900	-0.36883800	2.98875600
H	-1.25066300	2.86004800	4.86347200
C	-5.32215900	-0.08397200	-0.84454600
C	4.59330400	-2.24440900	-0.58620000
C	2.67240800	4.20462700	-1.01047400
F	3.45280800	3.87172000	0.02071900
F	3.45622800	4.70836100	-1.96412100
F	1.87577200	5.19091900	-0.58987500
F	-6.31130300	-0.75228200	-1.43917000
F	-5.29165000	1.14114000	-1.37536500

F	-5.65465400	0.06208300	0.44076400
F	5.23248400	-3.41340500	-0.58680600
F	5.36436800	-1.36976700	0.06524800
F	4.51538100	-1.82358700	-1.85126200

Meta-Product:

0 1

C	-2.73270000	0.41529300	-4.35208400
C	-1.43677400	0.08594600	-4.75583500
C	-0.40735500	-0.04220500	-3.82181200
C	-0.68874700	0.16891200	-2.46750200
C	-1.99134700	0.48061800	-2.07089100
C	-3.01647400	0.60853700	-3.00080300
H	-3.52357800	0.51395600	-5.09688200
H	0.59262900	-0.30965600	-4.16055700
H	-4.01957800	0.85246600	-2.64834000
H	-1.22121000	-0.07627800	-5.81249600
C	-2.19988600	0.62979800	-0.58652300
O	-3.28794100	0.86918100	-0.10257900
O	-1.08828700	0.47042700	0.05038100
P	0.51400100	-0.01526500	-1.09683500
C	1.26335400	1.42497200	-0.24555500
C	2.52182000	1.28311200	0.34549400
C	0.62287300	2.66538900	-0.23769500
C	3.12989400	2.37322200	0.96852000
H	3.04378800	0.32396700	0.31433500
C	1.25832100	3.75573900	0.34940600
H	-0.35791400	2.79134100	-0.69730700
C	2.50473300	3.61587600	0.96326600
C	2.04497700	-0.39069700	-2.13659200
C	2.63619500	-1.65015800	-2.25924000
C	2.60993200	0.68204900	-2.84688400
C	3.75611600	-1.84297300	-3.07744200
H	2.23864400	-2.50824900	-1.71692600
C	3.71825400	0.48688500	-3.65971900
H	2.18396500	1.68563600	-2.76967300
C	4.30050400	-0.77935800	-3.78266500
C	0.39631300	-1.57590300	-0.16501600
C	-0.10540300	-2.70511400	-0.80901900
C	0.78696500	-1.64485200	1.17677600
C	-0.19337000	-3.90866200	-0.10645900
H	-0.42348200	-2.66298400	-1.85483300
C	0.69536500	-2.85190500	1.86375400
H	1.14995600	-0.75433100	1.69315300
C	0.20718900	-3.99152700	1.22376100
H	4.10473000	2.25287400	1.44124200
H	4.20139300	-2.83511500	-3.15707900
H	1.00139300	-2.90426300	2.90879200
H	5.17395100	-0.92365100	-4.42124600
H	0.13429800	-4.93954600	1.75652400
H	2.98599600	4.47983300	1.42406700
C	0.57884400	5.09877800	0.36649500
C	4.32903500	1.63362500	-4.41812800
C	-0.72410200	-5.11618400	-0.83078200
F	4.31779100	1.40968900	-5.73638600
F	5.60731100	1.81785200	-4.07284900
F	3.68802200	2.78254100	-4.20347200
F	1.45733500	6.09143700	0.20460800
F	-0.33513900	5.20728400	-0.59863100
F	-0.04638800	5.32186300	1.52660400
F	-0.00444400	-5.37840400	-1.92712400
F	-0.70479300	-6.20866800	-0.06825200
F	-1.98380400	-4.93184900	-1.23519100

Meta-TS2(b): (ν = 485.39i(cm^{-1}))

0 1

C	-1.59792500	-2.28867600	3.99825200
C	-0.26897400	-2.56240500	3.62772000
C	0.19274900	-2.54513700	2.31342800
C	-0.72680300	-2.15099400	1.29052900
C	-2.13201100	-2.09968200	1.66739400
C	-2.55661800	-2.15391000	2.98390800
C	-2.56538400	-2.74866300	0.41461800
O	-3.60695800	-2.98247200	-0.13976100
O	-1.33089100	-3.09138100	-0.03574300
P	-0.07021300	-0.85150700	0.11007000
C	-1.30772800	-0.39800000	-1.12586000
C	-2.42281400	0.31753100	-0.67187000
C	-1.23688700	-0.83029800	-2.45324000
C	-3.46485900	0.57897800	-1.55607600
C	-2.28390800	-0.55112600	-3.32860700
C	-3.40414300	0.14465800	-2.88051300
C	1.45004100	-1.47936400	-0.64181600
C	2.67184800	-0.82963600	-0.45575000
C	1.39686300	-2.69490400	-1.34778900
C	3.82969900	-1.38398600	-1.00299400
C	2.56044000	-3.22744100	-1.89237700
C	3.78159700	-2.57243000	-1.72481500
C	0.26644100	0.67049300	1.02880400
C	-0.06762400	0.78001300	2.38318900
C	0.77722600	1.77719500	0.33535100
C	0.13324800	1.99186100	3.04792000
C	0.98612900	2.96975800	1.01624400
C	0.66635500	3.08330000	2.37239800
H	-1.89043600	-2.30773600	5.04714200
H	1.24252000	-2.74571200	2.09025400
H	-3.62523900	-2.19338900	3.21051900
H	0.45655100	-2.78421400	4.41477300
H	-2.48855500	0.66974100	0.35943700
H	-0.36834200	-1.38407500	-2.81195100
H	2.73957800	0.09662300	0.11783100
H	-2.22628000	-0.88360900	-4.36490100
H	0.44701600	-3.22458200	-1.44542800
H	2.51850700	-4.16633800	-2.44434900

H	-0.49258800	-0.06518100	2.92735000
H	1.00621900	1.71998100	-0.73141200
H	-0.13283100	2.07806800	4.10122000
H	4.69622300	-2.99070600	-2.14541200
H	-4.22994200	0.35503200	-3.56250900
H	0.82536300	4.03002700	2.89157300
C	5.13181900	-0.65964600	-0.78271200
C	-4.66749100	1.36522400	-1.10554300
C	1.59265800	4.15329300	0.30912100
F	0.93889800	5.28063500	0.59531500
F	2.86287200	4.33686700	0.67923300
F	1.58230900	4.00340800	-1.01512300
F	6.16202300	-1.29827700	-1.33342500
F	5.39362600	-0.52015300	0.51976700
F	5.09650500	0.57056500	-1.30172700
F	-5.80458400	0.73588000	-1.40919200
F	-4.66246700	1.57842800	0.21025500
F	-4.71645200	2.55969800	-1.70228700

Para-PPhe$_3$:

0 1

P	0.00392300	0.10203500	1.88298300
C	1.57785000	-0.42708700	1.07624800
C	2.24007500	-1.51202300	1.66605100
C	2.12852400	0.17243000	-0.06329900
C	3.42161300	-2.00677000	1.12094000
H	1.82515600	-1.98097900	2.56157000
C	3.31721700	-0.31118400	-0.60621400
H	1.62721800	1.01700900	-0.53979800
C	3.95820700	-1.39942000	-0.01352700
H	3.92690100	-2.85758300	1.57879100
H	3.74086900	0.15346900	-1.49813900
C	-0.36657100	1.66509100	0.97638400
C	-1.24366900	1.75850700	-0.10823700
C	0.27077100	2.82572200	1.44333400
C	-1.47936800	2.98956800	-0.72275000
H	-1.75068500	0.86902400	-0.48595800
C	0.05114700	4.05105500	0.82611000
H	0.94714500	2.76967100	2.29974700
C	-0.83065600	4.12852000	-0.25509900
H	-2.16429000	3.05873600	-1.56811800
H	0.55637900	4.94806400	1.18884000
C	-1.19654400	-1.06786100	1.10695700
C	-2.47423200	-1.13698900	1.68370500
C	-0.89972300	-1.87976900	0.00781500
C	-3.44110200	-1.98871400	1.16201700
H	-2.71422000	-0.51986600	2.55283800
C	-1.86287900	-2.74499800	-0.51312600
H	0.08842000	-1.84337600	-0.45400500
C	-3.12823800	-2.79443800	0.06419900
H	-4.43293700	-2.03848200	1.61523200
H	-1.62607400	-3.37770100	-1.36865900
C	-1.08070000	5.47251100	-0.88176200
C	5.26441900	-1.87687600	-0.58588900
C	-4.18928500	-3.71480800	-0.47383400
F	-1.81575900	5.38558400	-1.99046600
F	0.06437400	6.08197300	-1.20544400
F	-1.72379900	6.29201300	-0.04285700
F	-5.24095500	-3.03190400	-0.93761100
F	-3.74129500	-4.47864700	-1.47033800
F	-4.65452200	-4.53083400	0.47806700
F	5.24404200	-1.88597000	-1.92187100
F	6.27820400	-1.08183900	-0.22388900
F	5.57030500	-3.10998900	-0.17946600

Para-In1(a):

0 1

P	0.94072300	-0.04541200	-0.05624500
C	0.38832300	-0.34450600	1.64584700
C	1.24003300	-0.00856300	2.70448400
C	-0.89632200	-0.83484000	1.89435300
C	0.79893200	-0.15848300	4.01479300
H	2.24717400	0.35831700	2.50091700
C	-1.33528400	-0.98818100	3.20825600
H	-1.56122100	-1.10378500	1.07131300
C	-0.48528000	-0.64910400	4.25846700
H	1.45172700	0.10674400	4.84746800
C	0.17920900	-1.31299100	-1.12101900
C	-1.06640600	-1.07397400	-1.71654200
C	0.82898100	-2.53926100	-1.30380900
C	-1.65632800	-2.05846000	-2.50506800
H	-1.58378000	-0.12360900	-1.57445900
C	0.23839800	-3.51731600	-2.09921400
H	1.79011600	-2.70284400	-0.80714500
C	-0.99708200	-3.27166200	-2.69741000
H	0.73694500	-4.47588400	-2.24725900
C	0.22697100	1.54415900	-0.57706200
C	0.33631400	1.91029500	-1.92500400
C	-0.33678600	2.42758400	0.34918200
C	-0.12210200	3.15463600	-2.34708700
H	0.77990200	1.22634800	-2.65251300
C	-0.79323800	3.67366300	-0.07374500
H	-0.42089700	2.15238400	1.40169400
C	-0.68468500	4.02868100	-1.41766100
H	-1.22839300	4.36883200	0.64581400
H	-2.62816000	-1.88105800	-2.96693000
H	-0.03806900	3.44579300	-3.39396900
H	-2.33648800	-1.36811000	3.41200200
C	4.80589200	1.07921500	-0.57693100
C	5.44139700	-0.09689600	-0.15230800
C	4.69961600	-1.20136700	0.27202300
C	3.28287400	-1.22899700	0.31826600
C	2.72773900	-0.01808900	-0.13846800

C	3.41830900	1.12955600	-0.57479900
H	5.39003300	1.94248200	-0.89983300
H	5.26964400	-2.08426300	0.58970800
H	2.89662300	2.03569000	-0.89372600
H	6.53433100	-0.13946700	-0.15204900
C	-0.92889900	-0.84659300	5.68455400
C	-1.60938100	-4.31348600	-3.59714000
C	-1.21381500	5.37005600	-1.85529600
F	-1.24508800	-4.13309600	-4.87031900
F	-1.23188800	-5.54550000	-3.25362200
F	-2.94261900	-4.27384300	-3.56399600
F	-0.42721600	-1.97271200	6.19996900
F	-2.25530800	-0.92127600	5.78870500
F	-0.51635400	0.15363700	6.46623500
F	-0.81448800	6.34222600	-1.03260400
F	-0.81190400	5.69023400	-3.08440500
F	-2.54994700	5.38779600	-1.85558600

Para-Product:

0 1

C	-2.70187100	0.43428200	-4.38582300
C	-1.40980700	0.09097300	-4.79047500
C	-0.37973500	-0.04200300	-3.85789600
C	-0.65617000	0.17786600	-2.50401200
C	-1.95556700	0.50094000	-2.10606300
C	-2.98119100	0.63500500	-3.03475700
H	-3.49324600	0.53693800	-5.12955500
H	0.61702200	-0.32033800	-4.19722800
H	-3.98153400	0.88848000	-2.68116300
H	-1.19785900	-0.07864100	-5.84673600
C	-2.16214400	0.65166900	-0.62140400
O	-3.24718200	0.90583700	-0.13799000
O	-1.05351100	0.47573800	0.01590300
P	0.54708900	-0.00779200	-1.13413000
C	1.29090200	1.44398000	-0.29513000
C	2.54505100	1.30796600	0.30400100
C	0.64538800	2.68469500	-0.30301300
C	3.14441600	2.40412900	0.92380400
H	3.07320500	0.35230300	0.28443800
C	1.26088500	3.78701800	0.27829300
H	-0.33388800	2.79673100	-0.76893900
C	2.50232800	3.63822100	0.90113700
C	2.08026200	-0.38722200	-2.16873200
C	2.68802100	-1.64296400	-2.24683500
C	2.63091900	0.67127100	-2.91171000
C	3.81143100	-1.84404900	-3.05335800
H	2.30128900	-2.48908500	-1.67917400
C	3.74360400	0.48003900	-3.72028600
H	2.18051700	1.66664200	-2.86534400
C	4.33354800	-0.78486600	-3.78671600
C	0.42411100	-1.56929000	-0.20205800
C	-0.11234000	-2.68835100	-0.84132900
C	0.84271800	-1.64597500	1.13015800
C	-0.21992600	-3.89561800	-0.15182600
H	-0.44742000	-2.63267900	-1.87957500
C	0.74246500	-2.85284000	1.81327300
H	1.23400800	-0.76626100	1.64358700
C	0.21352200	-3.97318700	1.16851500
H	-0.63746100	-4.77329900	-0.64472700
H	4.11588500	2.29821200	1.40624900
H	4.15241800	1.30784600	-4.30123800
H	0.77068100	4.76153800	0.25352700
H	4.27313800	-2.83040500	-3.11021900
H	1.07124100	-2.91995600	2.85183900
C	3.12261900	4.83963000	1.56319300
C	5.56762400	-0.98191300	-4.62415500
C	0.11613900	-5.26326100	1.93781600
F	2.44290500	5.19651500	2.65748500
F	4.38269300	4.61677000	1.93481300
F	3.12354500	5.89828100	0.74825900
F	1.30981900	-5.63924100	2.40739100
F	-0.35213300	-6.26187000	1.19006100
F	-0.69113400	-5.14224700	2.99529100
F	6.67679600	-0.64709900	-3.95511300
F	5.53979400	-0.22816700	-5.72613900
F	5.71288800	-2.25164400	-5.00668600

Para-TS2(b): (ν = 487.22i(cm^{-1}))

0 1

C	0.69748300	-1.09440400	4.69733900
C	-0.62884300	-1.13722600	4.23187300
C	-1.09323100	-0.41495300	3.13470800
C	-0.14194700	0.35647700	2.39504200
C	1.15277400	0.56610300	3.02717200
C	1.56754900	-0.14740400	4.13858200
C	1.18944400	1.99094600	2.64410500
O	1.98497700	2.89198500	2.68638700
O	-0.08474800	2.07690900	2.18269700
P	-0.20271700	0.09120700	0.53837300
C	1.02338400	1.10530900	-0.32000600
C	2.36778700	0.75224200	-0.15365600
C	0.67285200	2.26003000	-1.02886100
C	3.36551000	1.56245600	-0.69124500
C	1.67037600	3.05764500	-1.57589300
C	3.01083600	2.70708900	-1.39977300
C	-1.88675100	0.43239100	-0.02806800
C	-2.65174900	-0.54504000	-0.67487000
C	-2.44578300	1.68307700	0.28062300
C	-3.96596800	-0.26378600	-1.03944700
C	-3.75712100	1.95950600	-0.09232200
C	-4.50743100	0.98775400	-0.75438900
C	0.26143700	-1.61909000	0.16280300
C	0.66085700	-2.49171200	1.18046300

C	0.32571700	-2.01844500	-1.18117900
C	1.10238900	-3.77338000	0.85094400
C	0.75712600	-3.29892900	-1.50338700
C	1.13901900	-4.17162900	-0.48189200
C	4.07764500	3.55991700	-2.03581700
C	-5.91372100	1.30330200	-1.19602200
C	1.57347500	-5.57025200	-0.83960200
F	-5.93266800	1.78828900	-2.44066500
F	-6.48550900	2.21589900	-0.41101800
F	-6.68723600	0.21679900	-1.18696700
F	2.27410200	-5.58736200	-1.97492400
F	2.32996400	-6.11349100	0.11293900
F	0.52235600	-6.37445900	-1.01757400
F	3.77241600	4.85662400	-1.97010800
F	5.26341600	3.39168700	-1.45111700
F	4.22868000	3.26005700	-3.32953100
H	0.99737100	-1.69382400	5.55573600
H	-2.13102400	-0.50518300	2.80803000
H	2.51337800	0.10270300	4.62617500
H	-1.34108900	-1.78813500	4.74565500
H	2.64487100	-0.14949200	0.39634100
H	-0.37284100	2.54108900	-1.15842100
H	-2.23940000	-1.53156100	-0.88769000
H	1.40671300	3.95678400	-2.13406800
H	4.41537800	1.30268500	-0.55617500
H	-1.86282200	2.42358800	0.83176200
H	-4.56925100	-1.02386900	-1.53649900
H	-4.20009200	2.92701700	0.14540600
H	0.64564400	-2.18557800	2.22752100
H	0.05284100	-1.32784600	-1.98268600
H	1.42652500	-4.45546300	1.63700300
H	0.81123100	-3.61363200	-2.54624300

Cl:

Meta-PPhe$_3$:

0 1

P	0.10328100	-0.59418600	-1.57495200
C	-1.44132200	-1.30383100	-0.85453300
C	-1.68513300	-2.66407500	-1.09208800
C	-2.36054200	-0.56290600	-0.10545000
C	-2.81952000	-3.28048400	-0.56867900
H	-0.97957700	-3.24683200	-1.68917600
C	-3.49738200	-1.19345800	0.39863200
H	-2.20449600	0.49783200	0.09667900
C	-3.73901900	-2.54747800	0.18109900
H	-2.99819100	-4.34088300	-0.75192300
H	-4.63551000	-3.01273000	0.59061800
C	-0.11664200	1.19808800	-1.19088600
C	0.34020200	1.79817900	-0.01255900
C	-0.79224100	1.97492600	-2.14143800
C	0.10681300	3.15599900	0.19728600
H	0.87002100	1.22356000	0.74886700
C	-1.02538800	3.32964000	-1.91020400
H	-1.14058900	1.51622000	-3.06988500
C	-0.57414600	3.93297300	-0.73726500
H	-1.55666700	3.92653200	-2.65263800
H	-0.74181700	4.99282100	-0.54542600
C	1.34387200	-1.07919500	-0.29457000
C	2.68960500	-0.87945400	-0.62905400
C	1.01981800	-1.61638500	0.95649200
C	3.68244600	-1.19836300	0.29288300
H	2.97119900	-0.47540400	-1.60409700
C	2.02945900	-1.94117600	1.86368200
H	-0.02354200	-1.78114400	1.23077600
C	3.36854200	-1.73212300	1.54173200
H	1.77068600	-2.35939400	2.83744400
H	4.16530800	-1.97920700	2.24313500
Cl	0.67402900	3.89513600	1.66913000
Cl	-4.63926300	-0.26295600	1.32913700
Cl	5.35158000	-0.93548100	-0.12912900

Meta-In1(a):

0 1

P	-0.15856300	-0.05974400	0.20893000
C	0.52427300	1.09070500	-1.01561300
C	1.21146200	2.22078700	-0.56343600
C	0.43344100	0.80573600	-2.38104500
C	1.81223300	3.05597600	-1.50070700
H	1.26642900	2.45579500	0.50047900
C	1.04150500	1.65882900	-3.30151800
H	-0.11141400	-0.07352300	-2.73034700
C	1.73462800	2.78646100	-2.86730300
H	0.97262200	1.44624600	-4.36864800
C	-1.64206800	-0.82250900	-0.52081800
C	-1.54465700	-2.03412400	-1.21632500
C	-2.86714600	-0.15344200	-0.41957200
C	-2.68387100	-2.58132100	-1.80612100
H	-0.59047800	-2.55671200	-1.30032800
C	-3.98814600	-0.72538000	-1.01228100
C	-3.91229300	-1.93295200	-1.70593800
H	-2.61426500	-3.52511000	-2.34770000
C	1.05127500	-1.40202100	0.40342300
C	0.70749600	-2.49966800	1.20567100
C	2.31369600	-1.32586800	-0.18916600
C	1.63085300	-3.52115500	1.40878400
H	-0.28006200	-2.55987600	1.66904900

C	3.22164400	-2.36201300	0.02676700
H	2.59986400	-0.47963600	-0.81547500
C	2.89390100	-3.45885200	0.81982100
H	1.36765900	-4.37737900	2.03045900
H	3.62425800	-4.25384800	0.97008200
H	2.21065700	3.46134000	-3.57869000
H	-4.80910800	-2.35389000	-2.16071700
H	-2.91336800	0.79835400	0.12002400
C	-0.06621500	1.15257700	4.08408200
C	-0.97353100	2.21930800	4.00902900
C	-1.60206000	2.54326800	2.80480800
C	-1.37941100	1.85170500	1.58741600
C	-0.46922200	0.78965400	1.75451700
C	0.19770000	0.41518600	2.93731100
H	0.42773300	0.90583500	5.02523900
H	-2.29723300	3.39271700	2.82543500
H	0.90464900	-0.41785800	2.96733100
H	-1.18404400	2.80306700	4.90986300
Cl	-5.51976900	0.08924000	-0.89075300
Cl	4.79060100	-2.28146000	-0.71442800
Cl	2.67077400	4.46400100	-0.95497000

Meta-Product:

0 1

C	-2.71293200	0.38920100	-4.38773300
C	-1.41709800	0.05700200	-4.78932200
C	-0.38603600	-0.05723600	-3.85534300
C	-0.66449400	0.17043200	-2.50302200
C	-1.96759200	0.48215400	-2.10826000
C	-2.99433700	0.59723300	-3.03825500
H	-3.50542700	0.47745500	-5.13215700
H	0.61388700	-0.32743000	-4.19183900
H	-3.99740600	0.84200200	-2.68625200
H	-1.20280100	-0.11861700	-5.84414200
C	-2.17794000	0.64151800	-0.62480200
O	-3.26836600	0.88491400	-0.14613300
O	-1.06940500	0.48511800	0.01494300
P	0.54353700	0.00107800	-1.13356300
C	1.28470300	1.45110500	-0.29028100
C	2.54700200	1.31588700	0.29520500
C	0.63030900	2.68449300	-0.28161300
C	3.14339100	2.41280400	0.91602200
H	3.07595300	0.36121900	0.26253600
C	1.26290900	3.77634300	0.30645800
H	-0.35618800	2.80856200	-0.72797100
C	2.50968900	3.65253600	0.91861600
C	2.07560100	-0.37353500	-2.16944100
C	2.69166500	-1.62525100	-2.24888000
C	2.61442300	0.69146300	-2.90875900
C	3.81638300	-1.81327900	-3.05926300
H	2.31234700	-2.47174700	-1.67692400
C	3.72861100	0.48905700	-3.71323300
H	2.17080600	1.68956900	-2.86667700
C	4.34243400	-0.76162700	-3.80000700
C	0.42749100	-1.55640900	-0.19535300
C	-0.09953600	-2.67800000	-0.83896500
C	0.84277400	-1.62781200	1.13623800
C	-0.19385100	-3.87485500	-0.13148100
H	-0.43354900	-2.64013500	-1.87808200
C	0.74366700	-2.83800800	1.82084800
H	1.22750400	-0.74354200	1.64662700
C	0.22663200	-3.96997300	1.19325700
H	4.11944600	2.30371700	1.38975000
H	4.28811500	-2.79542300	-3.11008400
H	1.06705100	-2.89994900	2.86048500
H	5.21815500	-0.89582900	-4.43498300
H	0.14293700	-4.91932600	1.72179000
Cl	4.37728400	1.82382400	-4.62409800
Cl	-0.84254700	-5.27821500	-0.92743900
H	2.97345100	4.52158200	1.38521200
Cl	0.47184800	5.32530400	0.28791000

Meta-TS2(b): (ν = 484.74i(cm^{-1}))

0 1

C	-0.15388900	-1.01935400	4.34520300
C	1.07355900	-0.60439600	3.79797000
C	1.40107100	-0.71126000	2.44813800
C	0.39546400	-1.20281400	1.55564000
C	-0.76114700	-1.83604700	2.17346400
C	-1.03761000	-1.73604700	3.52607500
C	-0.76432100	-2.94486900	1.19946300
O	-1.48202200	-3.86957300	0.92174400
O	0.42262400	-2.65448100	0.60686500
P	0.13173700	-0.15740300	0.01778900
C	-1.16585100	-0.85514700	-1.02812700
C	-2.46955900	-0.80070400	-0.52084100
C	-0.89142600	-1.52399800	-2.22519200
C	-3.48818700	-1.44335800	-1.22029300
C	-1.93034100	-2.14463500	-2.91355600
C	-3.23136700	-2.11758300	-2.41184900
C	1.70731900	-0.01600200	-0.85664600
C	2.34406900	-1.20532400	-1.24405300
C	2.31913400	1.22367500	-1.07422800
C	3.57816900	-1.12491100	-1.88032100
C	3.56156300	1.27334600	-1.70587300
C	4.19449300	0.10424400	-2.11835100
C	-0.47689700	1.47573100	0.50393700
C	-0.75429700	1.77004500	1.84333700
C	-0.77084500	2.40256200	-0.50665800
C	-1.30703900	3.01017900	2.16846100

C	-1.31410500	3.63271500	-0.15473600
C	-1.58335500	3.94769300	1.17855800
H	-0.35028800	-0.89639400	5.40932100
H	2.36417200	-0.35224700	2.07977700
H	-1.87309200	-2.29556900	3.95504400
H	1.81460000	-0.15380900	4.46357400
H	-2.70170000	-0.27152100	0.40531100
H	0.12282900	-1.56357200	-2.62348000
H	1.89246800	-2.17680600	-1.03457600
H	-1.72601700	-2.66197900	-3.85121800
H	1.84195000	2.14862200	-0.74981900
H	4.04262800	2.23674100	-1.87668400
H	-0.55572500	1.04455600	2.63313500
H	-0.59192200	2.17693200	-1.56025600
H	-1.52837600	3.24465100	3.20997500
H	5.16400400	0.13616800	-2.61568600
H	-4.04701800	-2.61055700	-2.94104800
H	-2.01523400	4.91741400	1.42656800
Cl	4.36639000	-2.58791500	-2.38246900
Cl	-5.10534700	-1.39547400	-0.59038700
Cl	-1.67602800	4.79051000	-1.39654400

Para-PPhe$_3$:

0 1

P	0.00325000	0.00106800	1.63897900
C	-0.99002800	1.31364300	0.80676100
C	-0.91622600	2.60429700	1.34970200
C	-1.80400000	1.09895100	-0.31167200
C	-1.62002700	3.66378800	0.78383100
H	-0.29776600	2.79007000	2.23153100
C	-2.52529500	2.14773500	-0.88179700
H	-1.88397500	0.10352900	-0.75287400
C	-2.42337600	3.42253800	-0.32986100
H	-1.55732400	4.66749200	1.20415100
C	-0.63948300	-1.51564400	0.80856300
C	-0.05464300	-2.10899100	-0.31619400
C	-1.79042900	-2.09833900	1.35789000
C	-0.60807800	-3.25469200	-0.88762800
H	0.84366300	-1.67754500	-0.76182300
C	-2.36125500	-3.23455900	0.79128900
H	-2.25386800	-1.65826300	2.24458100
C	-1.75928600	-3.80485100	-0.32965800
H	-3.25820300	-3.68435900	1.21695900
C	1.63812100	0.20412400	0.80938500
C	2.72082300	-0.49581600	1.36094500
C	1.85723900	1.00560200	-0.31710700
C	3.98996900	-0.42108800	0.79335500
H	2.57338500	-1.11366700	2.25045800
C	3.12566200	1.10075600	-0.88893600
H	1.03365200	1.56623600	-0.76386800
C	4.18002400	0.38215200	-0.33036500
H	3.29794400	1.72563400	-1.76540700
H	-0.15195600	-3.71821400	-1.76249100
H	4.82934100	-0.97008700	1.22039500
H	-3.16134300	1.98001300	-1.75121900
Cl	5.76227600	0.49406700	-1.04377000
Cl	-2.45450600	-5.23555000	-1.03717300
Cl	-3.31839100	4.73651300	-1.03706200

Para-In1(a):

0 1

P	0.95019700	-0.04210500	-0.05975400
C	0.40279100	-0.34815600	1.63845800
C	1.24405400	0.00198100	2.70053700
C	-0.87198200	-0.86412800	1.89225100
C	0.81097800	-0.15563400	4.01332400
H	2.24602300	0.38623500	2.50126400
C	-1.31119200	-1.02772600	3.20363900
H	-1.53159100	-1.14985000	1.07014700
C	-0.46423700	-0.66880400	4.25111500
H	1.45537900	0.11072300	4.85066100
C	0.19044000	-1.30042500	-1.13003700
C	-1.04907200	-1.05521500	-1.73591400
C	0.82663800	-2.53543500	-1.31019100
C	-1.64828400	-2.03340100	-2.52541800
H	-1.55824600	-0.09872100	-1.60319800
C	0.23426500	-3.51301200	-2.10377600
H	1.78681600	-2.70691800	-0.81339900
C	-0.99747200	-3.25297200	-2.70358600
H	0.72077500	-4.47619300	-2.25728300
C	0.24036700	1.54746300	-0.57359900
C	0.37324800	1.93745700	-1.91453400
C	-0.36882600	2.40866300	0.34334400
C	-0.10164500	3.17363800	-2.33676700
H	0.84780900	1.27350300	-2.64141100
C	-0.84785300	3.65021700	-0.07187100
H	-0.47782600	2.11906700	1.38999900
C	-0.70981500	4.01976600	-1.40742800
H	-1.32637600	4.32744600	0.63510900
H	-2.61106300	-1.85290300	-3.00289000
H	-0.00570500	3.48459800	-3.37669300
H	-2.29978300	-1.43439000	3.41489800
Cl	-1.31069200	5.56060100	-1.92994300
Cl	-1.73804600	-4.47487200	-3.69029000
Cl	-1.00457200	-0.87276900	5.88793700
C	4.81770200	1.09266600	-0.55384900
C	5.45782800	-0.08827500	-0.15193600
C	4.71881700	-1.20291800	0.25105200
C	3.30225500	-1.23817600	0.29660500
C	2.74079400	-0.02094200	-0.13789400
C	3.42939700	1.13630800	-0.55091600
H	5.39786000	1.96473800	-0.86014700

H	5.29250800	-2.08941100	0.55255100
H	2.90457600	2.04672400	-0.85199300
H	6.55098700	-0.12721300	-0.15210500

Para-Product:

0 1

C	-2.70708800	0.40654700	-4.38260100
C	-1.41173100	0.07751100	-4.78792100
C	-0.38044000	-0.04403500	-3.85511400
C	-0.65677300	0.17362800	-2.50057300
C	-1.95951600	0.48293700	-2.10181200
C	-2.98636800	0.60442300	-3.03119800
H	-3.50054000	0.50022400	-5.12539400
H	0.61914800	-0.31239900	-4.19411100
H	-3.98885800	0.84648700	-2.67557400
H	-1.19811000	-0.09030000	-5.84417700
C	-2.17458200	0.63469600	-0.61666400
O	-3.27131000	0.87279200	-0.14584300
O	-1.07136300	0.47995600	0.02621500
P	0.56316800	-0.00499700	-1.14298200
C	1.28680200	1.45112200	-0.30320900
C	2.55493200	1.33456300	0.27468700
C	0.62384200	2.68130200	-0.27982000
C	3.14750600	2.42875000	0.90101900
H	3.10058500	0.38905700	0.23672400
C	1.22495900	3.79003400	0.30823800
H	-0.36582300	2.78549500	-0.72507900
C	2.47747500	3.64983200	0.90548900
C	2.08763000	-0.38248000	-2.17681600
C	2.70044500	-1.63663100	-2.24791200
C	2.64081200	0.67018000	-2.92544500
C	3.82745200	-1.84449800	-3.04747400
H	2.31734800	-2.48017700	-1.67325800
C	3.75702500	0.48064200	-3.73104600
H	2.19317700	1.66728100	-2.88378700
C	4.34363000	-0.78503400	-3.78378700
C	0.43699900	-1.55681700	-0.20194300
C	-0.10142900	-2.68341800	-0.82934800
C	0.85666600	-1.62866100	1.12845200
C	-0.21259500	-3.88551100	-0.13388800
H	-0.43931500	-2.63757600	-1.86751100
C	0.75635100	-2.82909800	1.82692900
H	1.25158500	-0.74721000	1.63690700
C	0.22316400	-3.94754400	1.18813900
H	-0.63292900	-4.76979600	-0.61235700
H	4.12740000	2.33975700	1.36936500
H	4.17767300	1.30192300	-4.31129700
H	0.72211900	4.75669400	0.31598900
H	4.30266000	-2.82421100	-3.09534900
H	1.08199800	-2.89836400	2.86465900
Cl	3.21638100	5.02556200	1.66466500

Cl	0.09308800	-5.44488700	2.05973600
Cl	5.74759000	-1.03394300	-4.77970800

Para-TS2(b): (ν = 486.73i(cm^{-1}))

0 1

C	0.71003400	-1.07513900	4.69123400
C	-0.61967000	-1.11418900	4.23394600
C	-1.08736400	-0.39760400	3.13502000
C	-0.13753000	0.36500300	2.38176600
C	1.16323700	0.57218500	3.00616700
C	1.58020400	-0.13504400	4.12058500
H	1.01230600	-1.66982200	5.55212800
H	-2.12772400	-0.48433100	2.81569400
H	2.52908900	0.11589300	4.60209600
H	-1.33179400	-1.75789300	4.75712200
C	1.19840800	1.99669900	2.62215100
O	1.99586600	2.89712100	2.65699700
O	-0.07996100	2.08521200	2.17447100
P	-0.20419300	0.08927500	0.53051800
C	-1.88782900	0.42802200	-0.02824400
C	-2.64991100	-0.54106300	-0.69150300
C	-2.45925800	1.67059800	0.29356300
C	-3.96526500	-0.26727900	-1.05655700
H	-2.23296000	-1.52227200	-0.92088000
C	-3.77088600	1.94835400	-0.07485100
H	-1.88226900	2.41074600	0.85196900
C	-4.51145600	0.97716300	-0.74958500
H	-4.56644600	-1.01611800	-1.57141700
C	1.01773000	1.09890500	-0.33268300
C	2.36300000	0.72973600	-0.20592200
C	0.67264500	2.27669100	-1.00507400
C	3.36216800	1.53634500	-0.74381600
H	2.64183400	-0.19017600	0.31252900
C	1.66513200	3.07924400	-1.55497100
H	-0.37144800	2.57581800	-1.10805400
C	3.00218400	2.70404500	-1.41242700
H	1.41005600	3.99314100	-2.09073300
C	0.25754500	-1.62079100	0.17030000
C	0.33251900	-2.03167600	-1.16972400
C	0.64774900	-2.49496900	1.19198700
C	0.76205600	-3.31413700	-1.48827900
H	0.06567500	-1.34724800	-1.97891100
C	1.08834800	-3.77885800	0.87609000
H	0.62473500	-2.18801700	2.23879800
C	1.13558000	-4.17813800	-0.45716800
H	1.39916700	-4.46606800	1.66233200
H	4.41235600	1.26216500	-0.64936600
H	0.81847300	-3.64375000	-2.52533200
H	-4.22505600	2.90906200	0.16649600
Cl	1.68528300	-5.77575600	-0.84597000
Cl	4.24138200	3.70778300	-2.09533100

Cl	-6.14919800	1.32064800	-1.20148800

CN:

Meta-$PPhe_3$:

0 1

P	0.07337300	-0.56000200	-1.51833000
C	-1.46529800	-1.25393100	-0.76884000
C	-1.73089800	-2.61109800	-1.00771600
C	-2.36739800	-0.51168100	-0.00530800
C	-2.86353900	-3.22470200	-0.47667400
H	-1.03936600	-3.19821300	-1.61760700
C	-3.51391700	-1.12940100	0.51503600
H	-2.19470200	0.54633800	0.19813900
C	-3.76516700	-2.48761400	0.28639000
H	-3.04999200	-4.28200600	-0.66657200
H	-4.65962300	-2.95300500	0.70062000
C	-0.11238300	1.23166500	-1.11166400
C	0.41680700	1.83040500	0.03309500
C	-0.82962000	2.01986600	-2.02375500
C	0.21789500	3.19983600	0.25842400
H	0.98295300	1.24722800	0.76121800
C	-1.03609500	3.37839900	-1.78986800
H	-1.23510800	1.56347100	-2.93036400
C	-0.51144300	3.97842700	-0.64867200
H	-1.60211900	3.97490800	-2.50561200
H	-0.65854600	5.04166900	-0.45853900
C	1.33419200	-1.07953400	-0.27113400
C	2.67452700	-0.94783500	-0.64685700
C	1.03011300	-1.58514700	0.99923500
C	3.69175800	-1.29830600	0.24900000
H	2.93752300	-0.57202400	-1.63828100
C	2.04701500	-1.94445300	1.88516000
H	-0.01083900	-1.69908200	1.30824100
C	3.38131400	-1.80035400	1.52013800
H	1.79431300	-2.33560500	2.87104500
H	4.18183800	-2.07542900	2.20688500
C	5.07066600	-1.15153500	-0.14772300
N	6.17462800	-1.03421000	-0.46578200
C	0.77145500	3.80888200	1.44294700
N	1.21496800	4.29385200	2.39259900
C	-4.44080000	-0.35192800	1.30039500
N	-5.18131600	0.27207400	1.92977100

Meta-In1(a):

0 1

P	-0.13904100	-0.05897500	0.21484300
C	0.54217200	1.09007300	-1.01337800
C	1.17467600	2.25498000	-0.57736800
C	0.48684500	0.77913500	-2.37635200
C	1.75999200	3.10368600	-1.52308500
H	1.20023100	2.50850700	0.48352300
C	1.07174800	1.63331400	-3.31057200
H	-0.01618500	-0.12839100	-2.71802700
C	1.71003400	2.79550200	-2.89024300
H	1.02578300	1.39142400	-4.37220000
C	-1.63217800	-0.80602300	-0.51512100
C	-1.55490600	-2.01863400	-1.21254700
C	-2.84952300	-0.12697700	-0.41194900
C	-2.69801100	-2.56040600	-1.80056200
H	-0.60609700	-2.55127400	-1.30146700
C	-3.98806600	-0.68546000	-1.00164800
C	-3.91780600	-1.90114300	-1.69661600
H	-2.63356700	-3.50459500	-2.34107100
C	1.06258300	-1.41052500	0.39851800
C	0.70766200	-2.52173200	1.17944200
C	2.33727000	-1.32619300	-0.15978600
C	1.62349100	-3.54785100	1.39354200
H	-0.28940300	-2.58878000	1.62233300
C	3.25214100	-2.36426400	0.06201500
H	2.63269100	-0.46840300	-0.76579400
C	2.89839000	-3.47539000	0.83645900
H	1.34315200	-4.40976600	1.99881000
H	3.62173400	-4.27468500	0.99792500
H	2.16725100	3.47037400	-3.61401700
H	-4.81602500	-2.31980200	-2.15106800
H	-2.87942900	0.82621400	0.12782500
C	-0.05495300	1.12953200	4.09524200
C	-0.95534500	2.20259900	4.01634800
C	-1.57178100	2.53717500	2.80912500
C	-1.34269000	1.84750200	1.59201100
C	-0.44032700	0.78077100	1.76324400
C	0.21553900	0.39502700	2.94879400
H	0.42848300	0.87648800	5.04005000
H	-2.26135000	3.39074600	2.82668500
H	0.91756800	-0.44215600	2.98128700
H	-1.16970000	2.78336200	4.91803000
C	2.41946100	4.30936300	-1.08521500
N	2.95038700	5.27386200	-0.73774200
C	-5.25115800	0.00423000	-0.89610200
N	-6.26450700	0.55153600	-0.81490600
C	4.57049300	-2.28187100	-0.51697500
N	5.62544200	-2.21517600	-0.98109800

Meta-Product:

0 1

C	-2.72363700	0.38438100	-4.39593800
C	-1.42639500	0.05600300	-4.79619700
C	-0.39535800	-0.05571400	-3.86180000
C	-0.67649200	0.17226500	-2.51031400
C	-1.98113300	0.47963300	-2.11731900
C	-3.00828400	0.59121000	-3.04683000
H	-3.51522600	0.47045400	-5.14146600

H	0.60417300	-0.32547100	-4.19948700
H	-4.01299100	0.83243200	-2.69718600
H	-1.21079700	-0.11896000	-5.85079500
C	-2.18629600	0.63692500	-0.63505100
O	-3.26968000	0.87210600	-0.14302500
O	-1.06749200	0.48706600	-0.00082300
P	0.51632200	0.00819100	-1.12707500
C	1.27792900	1.44654900	-0.27458900
C	2.55107000	1.30500100	0.28802800
C	0.62684900	2.67836300	-0.22484700
C	3.16335300	2.38423600	0.92491900
H	3.07972600	0.35101500	0.22788700
C	1.26688800	3.76760000	0.37727100
H	-0.36742600	2.80636300	-0.65210200
C	2.53144400	3.62170500	0.96482500
C	2.05883600	-0.36451800	-2.15982400
C	2.69998700	-1.60569300	-2.21947300
C	2.58822300	0.69294100	-2.91188000
C	3.83379900	-1.79560800	-3.01711400
H	2.33232900	-2.45000000	-1.63582400
C	3.71631100	0.50228100	-3.71390700
H	2.12434600	1.68277500	-2.88561200
C	4.34740200	-0.74988500	-3.77060600
C	0.40922000	-1.55818300	-0.19904500
C	-0.10026100	-2.68296800	-0.84520500
C	0.80099500	-1.63009400	1.14108700
C	-0.20288500	-3.88890200	-0.13934600
H	-0.41913000	-2.64260100	-1.88917200
C	0.70257800	-2.83692000	1.83144600
H	1.17142500	-0.74239400	1.65735700
C	0.20252500	-3.97121100	1.19852600
H	4.14658300	2.25949000	1.37840000
H	4.31714800	-2.77241400	-3.04455500
H	1.01104400	-2.88957400	2.87558400
H	5.22953700	-0.88842000	-4.39584100
H	0.11897300	-4.91683500	1.73405500
H	3.00977400	4.47633100	1.44342800
C	0.60979100	5.05114700	0.40225400
N	0.08609800	6.07997400	0.42196000
C	4.23695700	1.60430900	-4.48512600
N	4.65378900	2.48595000	-5.10363300
C	-0.72826500	-5.05676300	-0.80256800
N	-1.14716100	-5.99289100	-1.33294600

Meta-TS2(b): (ν = 484.74i(cm^{-1}))

0 1

C	-1.79887500	-1.39793900	3.89894100
C	-0.47442700	-1.80653300	3.66069900
C	0.08592000	-1.93208100	2.39081900
C	-0.71867600	-1.55489500	1.26995900
C	-2.13827000	-1.36252900	1.52557800
C	-2.66356300	-1.27091100	2.80340500
C	-2.53084900	-2.07821000	0.29604100
O	-3.54260200	-2.27320300	-0.32384300
O	-1.29854400	-2.55418100	-0.02365100
P	0.11407800	-0.40434000	0.03999800
C	-1.01559800	0.01518000	-1.30732100
C	-2.09456300	0.84569600	-0.99365400
C	-0.91430100	-0.56709900	-2.57636000
C	-3.08246500	1.07339800	-1.95993200
C	-1.89452700	-0.32071100	-3.53269500
C	-2.98668300	0.48886300	-3.22804600
C	1.62946900	-1.18409100	-0.56733900
C	2.87428000	-0.57066100	-0.41204100
C	1.53775900	-2.46844700	-1.13119400
C	4.02337900	-1.23807500	-0.85291000
C	2.68927200	-3.11592000	-1.57118400
C	3.93401500	-2.50651700	-1.43941500
C	0.49387100	1.17074400	0.84909000
C	0.14487200	1.39786300	2.18644300
C	1.05770500	2.19739800	0.08276800
C	0.38875300	2.64543900	2.76308800
C	1.30441900	3.43867200	0.67577300
C	0.97340700	3.66383000	2.02014100
H	-2.17031500	-1.30768900	4.91867000
H	1.12825600	-2.23322100	2.27035900
H	-3.74539400	-1.20583800	2.94500300
H	0.16710600	-2.02343900	4.51881300
H	-2.18438600	1.31329100	-0.01115300
H	-0.07150100	-1.21368000	-2.82493100
H	2.97394700	0.41037700	0.05321500
H	-1.80987000	-0.76876000	-4.52246600
H	0.56582000	-2.96074500	-1.20381900
H	2.61398600	-4.10956400	-2.01252400
H	-0.32399800	0.61477300	2.78422500
H	1.29954100	2.04984600	-0.97231400
H	0.11536400	2.82016700	3.80335900
H	4.83871800	-3.01169800	-1.77810400
H	-3.76305300	0.67355000	-3.97051200
H	1.16666000	4.63825200	2.46914900
C	1.89573500	4.49578000	-0.10709900
N	2.37054100	5.34211900	-0.73220600
C	5.31171800	-0.60955000	-0.69359800
N	6.34374100	-0.10819900	-0.56617700
C	-4.20627100	1.91762200	-1.63862400
N	-5.10513100	2.59474300	-1.38090000

Para-$PPhe_3$:

0 1

P	0.00004400	0.00503700	1.63042400
C	-0.99432300	1.31372700	0.79016500
C	-0.89946600	2.60949900	1.31952300

C	-1.82565300	1.08466800	-0.31280100
C	-1.60084600	3.66474500	0.74864900
H	-0.26689800	2.79885800	2.19001700
C	-2.54352800	2.13102900	-0.88541400
H	-1.91763500	0.08419600	-0.73868200
C	-2.42777200	3.42278500	-0.35712200
H	-1.51818200	4.67125900	1.15913100
C	-0.63921100	-1.51678000	0.80335400
C	-0.05558400	-2.10417400	-0.32576000
C	-1.78305100	-2.10037800	1.36719100
C	-0.60796500	-3.25071900	-0.88983800
H	0.83558600	-1.66580300	-0.77808900
C	-2.35067200	-3.23843700	0.80616800
H	-2.23795600	-1.65897300	2.25698000
C	-1.75830000	-3.81603700	-0.32517800
H	-3.24263900	-3.68623300	1.24447500
C	1.63506400	0.20792000	0.79732900
C	2.69713200	-0.55092600	1.31179200
C	1.86854400	1.06807500	-0.28235900
C	3.96542300	-0.47289800	0.74957600
H	2.53068000	-1.21497600	2.16337200
C	3.13829600	1.16507900	-0.84545600
H	1.05835300	1.66990800	-0.69720200
C	4.18621400	0.39155400	-0.33185700
H	3.31810900	1.83639400	-1.68543200
H	-0.15255500	-3.70651100	-1.76921000
H	4.78604900	-1.06929700	1.14856100
H	-3.18999800	1.94967200	-1.74438100
C	-3.16577600	4.50885200	-0.95273400
N	-3.75585400	5.37840700	-1.43192300
C	5.50112000	0.48954400	-0.91530300
N	6.55408100	0.56954700	-1.38311100
C	-2.33312200	-5.00309300	-0.90794300
N	-2.79332500	-5.95414800	-1.37437400

Para-In1(a):

0 1

P	0.95027600	-0.03332000	-0.05764400
C	0.39963800	-0.34407700	1.64400200
C	1.25325600	-0.01526700	2.70273500
C	-0.88238900	-0.84526700	1.89033300
C	0.82111400	-0.17997700	4.01361200
H	2.25919900	0.35528100	2.50001400
C	-1.31861500	-1.01523600	3.20058000
H	-1.54694500	-1.11266400	1.06660500
C	-0.46531100	-0.67957800	4.25902000
H	1.47692100	0.07257800	4.84652400
C	0.18623000	-1.29930600	-1.12531500
C	-1.05374900	-1.05125500	-1.72909300
C	0.83003800	-2.53006100	-1.30281500
C	-1.64898900	-2.03009600	-2.51829000
H	-1.56397600	-0.09598400	-1.59483300
C	0.24017300	-3.50689900	-2.09744500
H	1.78965700	-2.69618800	-0.80349300
C	-0.99798100	-3.25592700	-2.70324100
H	0.73541300	-4.46620100	-2.24790200
C	0.22482400	1.55397900	-0.57312500
C	0.36440900	1.93659300	-1.91541300
C	-0.38785600	2.41148000	0.34474400
C	-0.11075300	3.17044800	-2.33994800
H	0.84362500	1.27075100	-2.63702600
C	-0.86584900	3.65069600	-0.07480700
H	-0.49829400	2.12196200	1.39087300
C	-0.72608300	4.02657700	-1.41509200
H	-1.34559200	4.32427800	0.63492000
H	-2.61281900	-1.84490500	-2.99211100
H	-0.00774700	3.47354500	-3.38162600
H	-2.31433800	-1.40903700	3.40316300
C	4.81904000	1.08686800	-0.56320500
C	5.44618400	-0.09778900	-0.14891300
C	4.69759100	-1.20234400	0.26269200
C	3.28070600	-1.22039700	0.30412900
C	2.73510600	-0.00218400	-0.14193200
C	3.43208200	1.14671200	-0.56455500
H	5.40973600	1.94943300	-0.87569900
H	5.26101600	-2.09172000	0.57326900
H	2.91688500	2.05945800	-0.87494400
H	6.53877700	-0.14671000	-0.14661000
C	-1.22145600	5.30898300	-1.85322700
N	-1.61783300	6.33402800	-2.20647600
C	-1.61083200	-4.27154200	-3.52478000
N	-2.10100400	-5.08403400	-4.18241200
C	-0.91537100	-0.85399100	5.61869900
N	-1.27681700	-0.99263100	6.70630600

Para-Product:

0 1

C	-2.72128400	0.40109000	-4.38212000
C	-1.43061900	0.05214400	-4.78667600
C	-0.39699500	-0.06946800	-3.85645100
C	-0.66932200	0.16787000	-2.50485200
C	-1.96755500	0.49521700	-2.10693600
C	-2.99686600	0.61829500	-3.03276000
H	-3.51490700	0.49482300	-5.12456900
H	0.59795100	-0.35304800	-4.19656900
H	-3.99639000	0.87569500	-2.67974200
H	-1.22248200	-0.13092700	-5.84142600
C	-2.16495200	0.65959900	-0.62392600
O	-3.24204300	0.92060400	-0.13064500
O	-1.04897600	0.48494600	0.00775300
P	0.52823800	-0.00149600	-1.12792700
C	1.28779700	1.45021200	-0.29726800
C	2.54523800	1.30699000	0.29754100

C	0.64817200	2.69288900	-0.30143300
C	3.15352600	2.39593700	0.91415200
H	3.06763500	0.34827900	0.27866400
C	1.26764100	3.79466800	0.27701500
H	-0.33383500	2.80978100	-0.76023000
C	2.51584700	3.64168600	0.89572600
C	2.06844500	-0.38332800	-2.15998700
C	2.71352900	-1.62313100	-2.19066700
C	2.59580200	0.66670000	-2.93165100
C	3.84972100	-1.81976200	-2.97672600
H	2.34962800	-2.46038700	-1.59542600
C	3.72042500	0.48646300	-3.72389700
H	2.11829900	1.65043500	-2.91907400
C	4.35108700	-0.76686000	-3.74575500
C	0.41769700	-1.56304100	-0.19156500
C	-0.10090900	-2.69093900	-0.83436800
C	0.82136900	-1.62979000	1.14462300
C	-0.20666800	-3.89450100	-0.14483900
H	-0.42429100	-2.64209000	-1.87659900
C	0.72661600	-2.83287000	1.83545300
H	1.19755500	-0.74443400	1.65965000
C	0.21386100	-3.96533000	1.18912000
H	-0.61158200	-4.77796200	-0.63818200
H	4.12518600	2.28409400	1.39493700
H	4.11556400	1.30752100	-4.32214400
H	0.78182600	4.77004800	0.26028600
H	4.34691500	-2.78977400	-2.99047700
H	1.04168600	-2.89485000	2.87702200
C	0.11539300	-5.21369200	1.90444200
N	0.03838600	-6.21351800	2.47684200
C	3.15088700	4.77929200	1.51465000
N	3.65980700	5.68945900	2.01033400
C	5.52435500	-0.96724200	-4.55993000
N	6.46375300	-1.12817200	-5.21204100

Para-TS2(b): (ν = 488.58i(cm^{-1}))

0 1

C	-1.33497900	1.47402200	4.05249200
C	0.02558300	1.64992500	3.74276200
C	0.71202900	0.89863000	2.79067400
C	-0.03628700	-0.05715100	2.03478400
C	-1.36481600	-0.37799600	2.53136500
C	-2.00582900	0.37411700	3.50080100
C	-1.18621400	-1.82283800	2.29289700
O	-1.86925700	-2.81168100	2.33048500
O	0.13767300	-1.78224400	1.98960500
P	0.22401200	0.02969900	0.17189700
C	-0.81264300	-1.17990800	-0.68528100
C	-2.19566700	-0.95664600	-0.65855000
C	-0.29033800	-2.34376000	-1.25928200
C	-3.06017500	-1.90787400	-1.19045800
C	-1.15008600	-3.28906600	-1.80385200
C	-2.53483900	-3.07293400	-1.76007100
C	1.98093800	-0.20642800	-0.19197500
C	2.73345100	0.78799900	-0.82858800
C	2.59721900	-1.38741900	0.25440300
C	4.09273100	0.59263400	-1.05177800
C	3.95336600	-1.58544200	0.02584100
C	4.69793900	-0.59743600	-0.63236600
C	-0.33698100	1.63980100	-0.44375000
C	-0.94446600	2.56268400	0.41552000
C	-0.24868100	1.90035500	-1.81960800
C	-1.44059800	3.75817800	-0.09993600
C	-0.73397600	3.09508100	-2.33402600
C	-1.32740100	4.02502300	-1.46819800
H	-1.81331900	2.10566500	4.79955100
H	1.76439200	1.09804000	2.57916000
H	-2.97126500	0.04637800	3.89486600
H	0.58100300	2.43476400	4.26287600
H	-2.60685100	-0.04373800	-0.22334600
H	0.78497700	-2.52081300	-1.28877200
H	2.27409500	1.72285000	-1.15018400
H	-0.75217000	-4.19588000	-2.25851200
H	-4.13755300	-1.74690500	-1.16651400
H	2.01807700	-2.13716600	0.79602700
H	4.68231700	1.36206100	-1.54952100
H	4.43817100	-2.50134700	0.36323800
H	-1.04494600	2.36194900	1.48307700
H	0.19282800	1.16830300	-2.49983000
H	-1.91651100	4.48190800	0.56120600
H	-0.66306300	3.30628700	-3.40065000
C	-1.83659500	5.26729400	-1.99723800
N	-2.24298000	6.26153700	-2.42006400
C	6.10558000	-0.80493100	-0.87176000
N	7.23118900	-0.97187900	-1.06554600
C	-3.42923500	-4.05923700	-2.31507800
N	-4.14704800	-4.84630200	-2.76015100

F:

Meta-PPhe$_3$:

0 1

P	0.02189700	-0.28237500	-1.46566600
C	-1.51018500	-0.96599000	-0.69480900
C	-1.91983200	-2.24050500	-1.11207100
C	-2.26349500	-0.28192800	0.26446700
C	-3.05498500	-2.83320400	-0.56076100
H	-1.34505700	-2.77426700	-1.87240900
C	-3.39842500	-0.88999800	0.78726100
H	-1.98793000	0.71274600	0.61888300
C	-3.81030300	-2.15757800	0.39729800
H	-3.36339300	-3.82756200	-0.88644000

H	-4.70828900	-2.59091600	0.83790300
C	-0.00979800	1.44760700	-0.82153000
C	0.64284900	1.85038900	0.34801100
C	-0.75254200	2.38470100	-1.55352700
C	0.52960600	3.17301900	0.75921000
H	1.23259600	1.15928400	0.95301600
C	-0.86011100	3.70272200	-1.11133100
H	-1.25488000	2.07990300	-2.47447800
C	-0.21378300	4.11105100	0.05474200
H	-1.44598900	4.42326200	-1.68346900
H	-0.27235300	5.13587600	0.42187800
C	1.32402500	-1.02808900	-0.38999600
C	2.64978000	-0.85485900	-0.80921000
C	1.06320600	-1.72823200	0.79382900
C	3.67859200	-1.36437500	-0.02958100
H	2.89643300	-0.32517100	-1.73216000
C	2.11724500	-2.24081000	1.55293100
H	0.03593400	-1.87263600	1.13180300
C	3.43823600	-2.06054100	1.14929900
H	1.90626300	-2.78523400	2.47438200
H	4.27874000	-2.44885300	1.72486200
F	4.94040800	-1.18784500	-0.43017900
F	-4.11709400	-0.22909000	1.69945000
F	1.15435000	3.55125900	1.87804400

Meta-In1(a):

0 1

P	-0.16184100	-0.06249700	0.20913000
C	0.52165400	1.08819600	-1.01456900
C	1.21556700	2.21361800	-0.56122700
C	0.42674600	0.80458100	-2.38025500
C	1.81160000	3.03895500	-1.50419400
H	1.28149200	2.46025700	0.49964600
C	1.03690300	1.65616000	-3.30208600
H	-0.12303000	-0.07189000	-2.72816500
C	1.73538600	2.78065400	-2.86866800
H	0.96393400	1.44395600	-4.36900500
C	-1.64522700	-0.82359400	-0.52085400
C	-1.54903500	-2.03681500	-1.21453000
C	-2.86744800	-0.14952000	-0.42383500
C	-2.68874500	-2.58142000	-1.80767800
H	-0.59631300	-2.56219100	-1.29408500
C	-3.97953600	-0.72450900	-1.02089900
C	-3.91543100	-1.92895900	-1.71281800
H	-2.61930300	-3.52622600	-2.34747000
C	1.05016800	-1.40239000	0.40363300
C	0.70951000	-2.50096100	1.20596100
C	2.31198000	-1.32169800	-0.18994400
C	1.63618200	-3.52082500	1.40896900
H	-0.27766900	-2.56359500	1.66927400
C	3.21310200	-2.35690500	0.03149900
H	2.60943800	-0.48146900	-0.81915200

C	2.89899800	-3.45547600	0.82025000
H	1.37411300	-4.37731700	2.03072100
H	3.64176600	-4.24083400	0.96152900
H	2.21642200	3.46428500	-3.56862000
H	-4.82048100	-2.33541000	-2.16509000
H	-2.92526700	0.80368600	0.11253300
C	-0.06329500	1.15234200	4.08362300
C	-0.97198600	2.21792500	4.01100400
C	-1.60453400	2.54031800	2.80836000
C	-1.38498100	1.84885600	1.59028100
C	-0.47267500	0.78793800	1.75489100
C	0.19785800	0.41499600	2.93607100
H	0.43370100	0.90618900	5.02335200
H	-2.30095100	3.38880400	2.83107400
H	0.90525600	-0.41772400	2.96415800
H	-1.18064100	2.80181800	4.91221400
F	-5.15273600	-0.09658700	-0.93304100
F	2.47511900	4.11662700	-1.08783800
F	4.41588500	-2.29104100	-0.53662400

Meta-Product:

0 1

C	-2.69488200	0.39282900	-4.39065200
C	-1.39724400	0.06414800	-4.78926200
C	-0.36930200	-0.05221100	-3.85213400
C	-0.65236200	0.16960900	-2.49977400
C	-1.95729900	0.47742000	-2.10786500
C	-2.98085300	0.59477100	-3.04126300
H	-3.48511000	0.48300800	-5.13728600
H	0.63238600	-0.31920500	-4.18578700
H	-3.98540900	0.83640200	-2.69131200
H	-1.17918600	-0.10698600	-5.84406300
C	-2.17498400	0.63014200	-0.62413200
O	-3.26976700	0.87020300	-0.15200600
O	-1.07108100	0.47273200	0.02060800
P	0.55585700	-0.00136800	-1.13074200
C	1.28043500	1.45712000	-0.28981500
C	2.54366700	1.33794400	0.29809100
C	0.61277700	2.68333900	-0.28891200
C	3.12892000	2.44506900	0.91328700
H	3.08281900	0.38912800	0.26971300
C	1.23872400	3.77521600	0.29636100
H	-0.37494000	2.81053900	-0.73203400
C	2.48229400	3.67818400	0.90994400
C	2.08708400	-0.37381800	-2.16354200
C	2.68486200	-1.63391300	-2.25749300
C	2.63652600	0.69161000	-2.89413700
C	3.80437600	-1.83003900	-3.07440200
H	2.29370300	-2.48002100	-1.69332700
C	3.74087600	0.47210300	-3.70075300

H	2.21897300	1.70088000	-2.85039400
C	4.34174900	-0.77869400	-3.80771100
C	0.43647200	-1.55904000	-0.19402900
C	-0.13465100	-2.66547100	-0.82692200
C	0.89285800	-1.64581300	1.12306700
C	-0.23112300	-3.85410700	-0.11449600
H	-0.50383600	-2.63048300	-1.85404600
C	0.78743000	-2.85738800	1.80669000
H	1.31369700	-0.77323100	1.62432300
C	0.22421500	-3.97419300	1.19247500
H	4.10592800	2.34732900	1.38746800
H	4.26132700	-2.81852300	-3.13600600
H	1.14261800	-2.93017800	2.83517400
H	5.21206800	-0.90362800	-4.45230700
H	0.12649300	-4.92990100	1.70761800
H	2.92385300	4.56353700	1.36782100
F	-0.77697000	-4.91506600	-0.70976400
F	4.24785200	1.49326500	-4.39492900
F	0.61813000	4.95511200	0.27835800

Meta-TS2(b): (ν = 4854.34i(cm^{-1}))

0 1

C	-1.32159600	-0.41137300	4.14486400
C	-0.06408300	0.18688800	3.94539400
C	0.69732500	0.03208500	2.78972700
C	0.12813100	-0.71687600	1.71034300
C	-1.05267300	-1.50829500	2.02786900
C	-1.76480900	-1.35264700	3.20463400
C	-0.56107300	-2.67501700	1.26956000
O	-0.99245500	-3.74500000	0.92765200
O	0.69068400	-2.20945600	1.02917700
P	0.23714300	0.13762400	0.04628200
C	-0.47025700	-0.86165100	-1.28238200
C	-1.85295100	-1.07851900	-1.23556700
C	0.31594400	-1.46009600	-2.27186700
C	-2.41848900	-1.91811100	-2.18591900
C	-0.28839100	-2.28501500	-3.21884100
C	-1.66062500	-2.52638600	-3.17848100
C	1.97378000	0.52976000	-0.26264700
C	2.88980800	-0.53367900	-0.26212400
C	2.41139200	1.85036100	-0.41092200
C	4.23279800	-0.23943600	-0.44359600
C	3.77231200	2.10718400	-0.58505300
C	4.69237400	1.06353800	-0.60839900
C	-0.76220600	1.64609500	0.08661700
C	-1.54377400	1.95908400	1.20421400
C	-0.81653200	2.43731400	-1.06996400
C	-2.36871900	3.08628500	1.16522200
C	-1.63800900	3.55381300	-1.06809400
C	-2.41673800	3.89449600	0.03410300
H	-1.87030000	-0.24051700	5.06989600
H	1.65966900	0.53719600	2.68520100
H	-2.59408900	-2.02749400	3.43277300
H	0.33976000	0.82758100	4.73378400
H	-2.49526600	-0.61464700	-0.48420900
H	1.39089500	-1.28413200	-2.31390200
H	2.57263000	-1.56590600	-0.10080100
H	0.31747400	-2.74746500	-3.99836400
H	1.70568100	2.68025900	-0.38269200
H	4.11757900	3.13480800	-0.70025800
H	-1.52594100	1.33523100	2.09867200
H	-0.24355700	2.20144700	-1.96939700
H	-2.98257500	3.33404100	2.03145500
H	5.75987900	1.23970500	-0.74248500
H	-2.15321800	-3.17107200	-3.90651300
H	-3.05188900	4.77933900	-0.01397600
F	5.11168700	-1.23882400	-0.45151600
F	-1.68798000	4.31839200	-2.15687600
F	-3.73119000	-2.13800200	-2.14496400

Para-$PPhe_3$:

0 1

P	-0.03243700	-0.03651800	1.46552100
C	1.50597500	0.61351700	0.68291500
C	2.72013600	0.33112000	1.32435100
C	1.52701600	1.34973600	-0.50925000
C	3.93339400	0.75250400	0.78480700
H	2.72085500	-0.23026100	2.26187900
C	2.73151600	1.78648300	-1.05762300
H	0.59347300	1.58649600	-1.02391300
C	3.91621300	1.47587000	-0.40145300
H	4.88533500	0.53905800	1.27152800
C	-1.31609800	0.98682400	0.62319000
C	-2.03586500	0.58513700	-0.50917000
C	-1.57132700	2.25258700	1.17166700
C	-2.98426900	1.42858400	-1.08772900
H	-1.86014100	-0.39651800	-0.95395800
C	-2.50523400	3.11116700	0.59856800
H	-1.02794900	2.57660700	2.06313000
C	-3.20008600	2.67976100	-0.52526800
H	-2.70875400	4.09908800	1.01244200
C	-0.23256200	-1.65142200	0.59768300
C	-1.25494000	-2.49796600	1.05298300
C	0.57020400	-2.07752500	-0.46715800
C	-1.49423000	-3.72760100	0.44580600
H	-1.88100200	-2.19139100	1.89502400
C	0.35307800	-3.31342800	-1.07665100
H	1.37746900	-1.44179300	-0.83497900

C	-0.68074500	-4.11526500	-0.61317200
H	0.97121400	-3.65832900	-1.90589700
H	-3.55526100	1.13135400	-1.96785000
H	-2.28975800	-4.39145500	0.78488500
H	2.76493900	2.36156700	-1.98330700
F	5.07265000	1.88998200	-0.92344600
F	-0.89967300	-5.29515900	-1.19860600
F	-4.10631500	3.49008300	-1.07737700

Para-In1(a):

0 1

P	0.11136500	-0.34958600	1.92614900
C	1.50236900	0.47386600	1.10157500
C	2.77008200	-0.12608300	1.16618100
C	1.34654800	1.70233000	0.45116000
C	3.87008900	0.49230200	0.58485900
H	2.90414200	-1.08579400	1.67168700
C	2.44406700	2.33042500	-0.13484400
H	0.36646400	2.17898100	0.39433900
C	3.68533600	1.71368800	-0.05759400
H	4.86431400	0.04734900	0.62094900
C	-1.38240300	0.58640800	1.51814400
C	-2.05240500	0.35608200	0.31153700
C	-1.82414300	1.58999600	2.38893000
C	-3.15977500	1.12715400	-0.03139200
H	-1.71784600	-0.43126900	-0.36712100
C	-2.92787700	2.36713000	2.05298800
H	-1.31037300	1.75445500	3.33795900
C	-3.57588000	2.12082700	0.84676400
H	-3.29754200	3.15242800	2.71214000
C	-0.04313900	-1.98596000	1.15118100
C	-0.78878900	-2.98806200	1.78910700
C	0.52342500	-2.22665900	-0.10798800
C	-0.95093400	-4.22747400	1.17827200
H	-1.22750600	-2.76756000	2.76774000
C	0.35931000	-3.46376000	-0.72575100
H	1.09745200	-1.45247500	-0.61973200
C	-0.37457200	-4.44374800	-0.06879000
H	0.79145200	-3.67557500	-1.70359600
H	-3.70365400	0.96685700	-0.96203400
H	-1.52069600	-5.02716100	1.65205400
H	2.34856400	3.28706900	-0.64815000
F	4.73684000	2.30707300	-0.61589200
F	-0.53465700	-5.62761800	-0.65615800
F	-4.63482100	2.85973400	0.52499400
C	1.57097200	0.11546500	5.68957900
C	0.54444400	-0.50382200	6.41598600
C	-0.56111600	-1.05547700	5.76439000
C	-0.74281600	-1.03585000	4.35842300
C	0.33917900	-0.41174000	3.70504100
C	1.47458900	0.16710500	4.30464200
H	2.43010800	0.55426300	6.19972600
H	-1.32526600	-1.51950300	6.40222000
H	2.25787000	0.65291100	3.71714500
H	0.61487700	-0.54625600	7.50682900

Para-Product:

0 1

C	-2.70218000	0.40827700	-4.37858400
C	-1.40524600	0.08601500	-4.78383000
C	-0.37511200	-0.03553700	-3.84979700
C	-0.65279500	0.17553200	-2.49426400
C	-1.95725800	0.47859600	-2.09542300
C	-2.98285900	0.59942400	-3.02656200
H	-3.49542600	0.50199800	-5.12163900
H	0.62589800	-0.29911300	-4.18836600
H	-3.98644100	0.83611400	-2.67033500
H	-1.18945500	-0.07663500	-5.84047200
C	-2.18010400	0.62650300	-0.60963500
O	-3.28436400	0.85594000	-0.14849400
O	-1.08219100	0.48002800	0.03854500
P	0.57576800	-0.00742600	-1.14457200
C	1.28601800	1.45047000	-0.30182900
C	2.55755000	1.34134400	0.27209600
C	0.61430500	2.67695600	-0.27604300
C	3.14632500	2.43869600	0.89578700
H	3.10704000	0.39826400	0.23146400
C	1.21047800	3.78961900	0.30888900
H	-0.37723000	2.77280400	-0.71854800
C	2.46317800	3.64787400	0.89576500
C	2.09522600	-0.38313600	-2.17842800
C	2.69893100	-1.64208200	-2.25949800
C	2.65274100	0.67210900	-2.92148100
C	3.82091100	-1.85286300	-3.06481700
H	2.31029600	-2.48505700	-1.68786600
C	3.76382100	0.48020900	-3.73341100
H	2.21131400	1.67132300	-2.86935200
C	4.33211900	-0.78907300	-3.79098300
C	0.43997600	-1.55611300	-0.20318300
C	-0.10102800	-2.68152200	-0.83195300
C	0.85664700	-1.62781200	1.12887800
C	-0.21721200	-3.88310400	-0.13671800
H	-0.43679800	-2.63326200	-1.87064800
C	0.75167300	-2.82719300	1.82824500
H	1.25237900	-0.74587200	1.63578400
C	0.21704700	-3.93541900	1.18186000
H	-0.63767000	-4.77469700	-0.60161100
H	4.12807900	2.37146000	1.36403400
H	4.19988200	1.29274000	-4.31481100
H	0.71424100	4.75980300	0.32705600
H	4.30070900	-2.82949500	-3.13108200
H	1.06958400	-2.91190000	2.86739700
F	3.02772200	4.70853500	1.47055700
F	5.40201900	-0.98183300	-4.56215600

F	0.11293500	-5.08378700	1.85003000

Para-TS2(b): (ν = 471.00i(cm^{-1}))

0 1

C	-2.97603000	0.92249700	3.11584600
C	-2.43975100	-0.37761400	3.11285600
C	-1.21082100	-0.71767300	2.55326400
C	-0.48934500	0.29826500	1.84392000
C	-0.95137400	1.66393800	2.07208400
C	-2.16097300	1.97083500	2.67123700
H	-3.95008300	1.11505400	3.56342500
H	-0.88605200	-1.76032500	2.55431800
H	-2.41709700	3.01371600	2.87641100
H	-3.02550800	-1.18392300	3.56265900
C	0.41786400	2.18409500	2.18917300
O	0.94764100	3.25983300	2.29827300
O	1.06038500	0.98527700	2.17125400
P	0.07087200	-0.17405400	0.11236500
C	1.49319900	-1.28363200	0.13617900
C	1.93793600	-1.82742900	1.34789300
C	2.16442400	-1.58215300	-1.05861400
C	3.03056900	-2.68881100	1.36407100
H	1.45035700	-1.55930300	2.28653500
C	3.25669900	-2.44262300	-1.04977200
H	1.84430800	-1.13679100	-2.00367700
C	3.66650500	-2.98470900	0.16400800
H	3.40233800	-3.12313600	2.29193500
H	3.79848100	-2.69223100	-1.96178400
C	0.49931900	1.27594500	-0.88096300
H	2.38065100	1.57974400	0.14839500
C	1.71151800	1.93934900	-0.63508600
C	-0.37167500	1.75058500	-1.86946100
H	-1.31101400	1.23686400	-2.07835100
C	2.04809700	3.06963700	-1.36845200
C	-0.04114300	2.88420900	-2.60698200
C	1.16253200	3.52464700	-2.34087500
H	-0.70156600	3.27418100	-3.38101800
H	2.98052500	3.60620000	-1.19461200
C	-1.34463500	-0.98633800	-0.66088700
C	-2.61730500	-0.45162000	-0.39970200
C	-1.21091900	-2.10191100	-1.49412100
C	-3.74447300	-1.01719700	-0.98402300
H	-2.73694800	0.40411200	0.27180700
C	-2.33626800	-2.67675000	-2.07906000
H	-0.23060500	-2.54080100	-1.68552400
C	-3.58198700	-2.12071100	-1.81588200
H	-4.74372600	-0.62295500	-0.80022500
H	-2.26267800	-3.54796500	-2.72956400
F	-4.65882800	-2.66682600	-2.37270300
F	4.71239100	-3.80500600	0.17726800
F	1.48083000	4.60944400	-3.04037900

Me:

Meta-PPhe$_3$:

0 1

P	0.23000300	0.00201700	-1.28008400
C	-0.47708000	1.41420300	-0.32540400
C	-1.61183500	2.03150200	-0.87326700
C	0.04678200	1.89841000	0.87657300
C	-2.24233000	3.10419400	-0.23911200
H	-2.01696200	1.66388800	-1.82155600
C	-0.56605600	2.98054800	1.51456800
H	0.92983000	1.43399100	1.31989700
C	-1.69883400	3.57402900	0.96567100
H	-0.15467000	3.35920500	2.45211600
C	1.77595900	-0.34772800	-0.33796100
C	1.88779400	-1.31933600	0.66064600
C	2.90191900	0.42001400	-0.67297300
C	3.10915300	-1.50600600	1.31314700
H	1.02594100	-1.93184600	0.93300300
C	4.12506300	0.25301300	-0.01959700
C	4.21353800	-0.72674500	0.97993300
H	3.19709600	-2.26727000	2.09037500
C	-0.85890400	-1.38617500	-0.74108400
C	-0.79845500	-2.56822200	-1.49295200
C	-1.71970800	-1.32258400	0.35905500
C	-1.57478700	-3.66642300	-1.13120700
H	-0.13980700	-2.62640100	-2.36333100
C	-2.51785300	-2.41519800	0.72569000
H	-1.77371500	-0.40573800	0.95276400
C	-2.42966500	-3.58842800	-0.03084400
H	-1.52193200	-4.58721700	-1.71469300
H	-3.04113300	-4.45111700	0.24350500
H	-2.17154300	4.41813300	1.47366000
H	5.16428300	-0.88120300	1.49583600
C	-3.46591400	3.75495300	-0.82949500
H	-4.31585300	3.69292600	-0.13414900
H	-3.28498300	4.82160200	-1.02740900
H	-3.75748000	3.27489000	-1.77215800
C	-3.45829400	-2.31058400	1.89808300
H	-2.97963400	-1.79977000	2.74497100
H	-4.35224400	-1.72786100	1.62780300
H	-3.79172100	-3.30175300	2.23165200
H	2.82147700	1.17100600	-1.46524000
C	5.32437100	1.09139400	-0.37768100
H	5.64441900	1.70171100	0.47978000
H	6.17645800	0.45722200	-0.66220200
H	5.10294200	1.76635900	-1.21409300

Meta-In1(a):

0 1

P	-0.17440700	-0.05290300	0.19453900
C	0.50393200	1.09409900	-1.03333100
C	1.25533600	2.19036600	-0.59406100
C	0.35542900	0.83518800	-2.39748300
C	1.87742000	3.04014500	-1.51172900
H	1.34423800	2.38877200	0.47674900
C	0.96925600	1.68383000	-3.31957400
H	-0.23845400	-0.01402100	-2.74149700
C	1.72126900	2.76958400	-2.87920000
H	0.85444400	1.49663900	-4.38802100
C	-1.65524300	-0.82885000	-0.52171500
C	-1.53974000	-2.02592200	-1.23764500
C	-2.89704300	-0.19126300	-0.40186300
C	-2.67770300	-2.58020200	-1.82421500
H	-0.57611900	-2.52859000	-1.33779900
C	-4.03945800	-0.74740200	-0.98357200
C	-3.91163100	-1.94842400	-1.69507100
H	-2.59788200	-3.51432000	-2.38191600
C	1.04299000	-1.38371600	0.41103000
C	0.68793700	-2.49627700	1.18642000
C	2.32863600	-1.28467600	-0.12845400
C	1.62756800	-3.49787900	1.41201100
H	-0.31499600	-2.58005300	1.61221200
C	3.27796600	-2.29086500	0.09051400
H	2.60337000	-0.41537600	-0.73080600
C	2.90869800	-3.39320200	0.86930900
H	1.36058400	-4.36570000	2.01658900
H	3.63918600	-4.18417400	1.05351000
H	2.19417200	3.43002300	-3.60980700
H	-4.79704900	-2.39387300	-2.15494900
C	2.70324800	4.21231600	-1.05287700
H	3.77159800	4.03164000	-1.24419900
H	2.42379000	5.12554700	-1.59640800
H	2.57584800	4.39284800	0.02169800
C	4.65211100	-2.20046000	-0.51786500
H	4.91241900	-1.16211000	-0.75996600
H	5.41249500	-2.60370000	0.16411900
H	4.70006600	-2.78473200	-1.44943300
H	-2.94500700	0.75070900	0.15374900
C	-5.37921800	-0.07061000	-0.86201200
H	-5.73445600	0.26807100	-1.84658000
H	-6.13335200	-0.76430400	-0.46361600
H	-5.32510300	0.80171200	-0.19914400
C	-0.09500800	1.13029000	4.08224100
C	-0.97882600	2.21617100	4.01884600
C	-1.59105000	2.56939200	2.81392100
C	-1.37548500	1.89418100	1.58572600
C	-0.48879500	0.80852300	1.74026300
C	0.15900200	0.40569800	2.92408200
H	0.38832700	0.85747200	5.02192600
H	-2.26912800	3.43307600	2.84370600
H	0.84710700	-0.44320200	2.94661500
H	-1.18373200	2.79102300	4.92693200

Meta-Product:

0 1

C	-1.98219700	0.38767800	-4.87609600
C	-0.62215500	0.10408800	-5.01634800
C	0.19271700	-0.05337700	-3.89429800
C	-0.36669600	0.07342000	-2.61732600
C	-1.72441900	0.37786900	-2.48562800
C	-2.53578100	0.53041600	-3.60478200
H	-2.60691900	0.50526600	-5.76278100
H	1.25271600	-0.26591400	-4.02744100
H	-3.58987900	0.76794100	-3.45443400
H	-0.18558100	0.00510300	-6.01099900
C	-2.23727000	0.57085800	-1.07662900
O	-3.40557200	0.85018900	-0.86045800
O	-1.30475100	0.40451800	-0.21665400
P	0.59734800	-0.03760800	-1.06072800
C	0.98427200	1.57004000	-0.30754100
C	1.15683400	2.67430900	-1.14301000
C	1.11479000	1.69988000	1.07773800
C	1.46993800	3.91144800	-0.57748000
H	1.05470500	2.57788300	-2.22666400
C	1.43712500	2.93362400	1.64903500
H	0.94713400	0.83733700	1.72690700
C	1.61383600	4.03637800	0.80113900
H	1.86236900	5.00799800	1.23431500
C	2.27073900	-0.50824900	-1.76010000
C	2.39810500	-1.79647800	-2.30069800
C	3.38809300	0.33259200	-1.75732700
C	3.59641600	-2.25230100	-2.85556400
H	1.53720700	-2.47391900	-2.29362500
C	4.59780600	-0.11307500	-2.29657100
H	3.33332500	1.33350600	-1.32891600
C	4.69996900	-1.38863700	-2.84366100
H	5.65058600	-1.72731500	-3.26177000
C	0.43978200	-1.49014600	0.03948100
C	-0.66256200	-2.34404200	-0.03775200
C	1.48995200	-1.77136300	0.91706800
C	-0.69581300	-3.48198600	0.76498600
H	-1.48387000	-2.12871200	-0.72209500
C	1.44301000	-2.88819600	1.75953500
H	2.36999100	-1.12224200	0.94565300
C	0.34041900	-3.74417900	1.66087900
H	0.29713500	-4.63412300	2.29270200
C	3.68456000	-3.63188500	-3.45393800
H	3.25330400	-4.38286900	-2.77738200
H	3.12336200	-3.68160200	-4.39908800
H	4.72549300	-3.90896100	-3.66373400
C	2.54751800	-3.15142700	2.74846500
H	2.33375800	-2.65521400	3.70760600
H	2.65296400	-4.22589100	2.94733300
H	3.50863200	-2.76600300	2.38319600

C	1.58377000	3.08632800	3.13971500
H	2.58751000	3.45482500	3.39655900
H	0.85870500	3.81511900	3.53011700
H	1.42417100	2.13178000	3.65663200
H	-1.54232400	-4.16656600	0.69679000
H	1.60365900	4.78127800	-1.22218300
H	5.46843100	0.54454700	-2.28486900

Meta-TS2(b): (ν = 487.51i(cm^{-1}))

0 1

C	-2.49778500	0.22945700	3.68380100
C	-1.39596500	1.10011700	3.60152900
C	-0.29173900	0.88442500	2.78089300
C	-0.31388900	-0.25302300	1.90891400
C	-1.34587000	-1.24752000	2.18185600
C	-2.40985300	-1.01082800	3.03413300
H	-3.33563600	0.46239800	4.33952000
H	0.52526800	1.60779000	2.74563400
H	-3.10541100	-1.82036600	3.27181400
H	-1.40715400	2.01296400	4.20341200
C	-0.40375300	-2.37152900	2.01874700
O	-0.46959200	-3.57338000	1.97513700
O	0.73203500	-1.63611000	1.95267500
P	0.13016000	0.11951000	0.13018500
C	1.74426400	0.93319400	0.12067300
C	2.82006200	0.27679900	0.74389000
C	1.91497200	2.21115100	-0.42003800
C	4.07955400	0.87557800	0.80260600
H	2.66329600	-0.70225500	1.20315400
C	3.17177200	2.81396800	-0.35600300
H	1.08125100	2.74222200	-0.87993700
C	4.23918700	2.15156100	0.24146600
H	5.21825700	2.63417900	0.28595400
C	-1.13442700	1.20048400	-0.57713900
C	-2.26123600	1.57382200	0.16053000
C	-1.00985900	1.57809700	-1.92391900
C	-3.24660800	2.34637400	-0.45904900
H	-2.38479700	1.26678400	1.19984100
C	-1.98604400	2.35920900	-2.54230300
H	-0.14201600	1.25495900	-2.50664200
C	-3.10650900	2.73791200	-1.78586300
H	-3.88516100	3.34299900	-2.25606600
C	0.10944800	-1.37728800	-0.88168600
C	1.28784900	-2.03172800	-1.25948700
C	-1.13928400	-1.93261400	-1.17972500
C	1.23740100	-3.25125000	-1.93572300
H	2.25989400	-1.59164600	-1.02791100
C	-1.19449700	-3.15735600	-1.84585800
H	-2.06025300	-1.41833900	-0.89695500
C	-0.02077500	-3.80657400	-2.21469900
H	-0.07525400	-4.76452800	-2.73707000
C	-1.85104300	2.78772500	-3.97869800
H	-1.73567800	3.87947000	-4.04737200
H	-0.97972900	2.32054300	-4.45441100
H	-2.74804200	2.51689300	-4.55328200
C	2.49563100	-3.96435700	-2.35309000
H	3.38945800	-3.39233400	-2.07441200
H	2.55691800	-4.95340600	-1.87628700
H	2.51172100	-4.12405300	-3.44076400
C	5.24314400	0.17710500	1.45384300
H	6.03505500	-0.02272900	0.71725200
H	5.68171600	0.80388300	2.24318200
H	4.93660200	-0.77778000	1.89817700
H	3.31202500	3.81129900	-0.77479000
H	-2.16225800	-3.60424000	-2.07676000
H	-4.13236900	2.63870500	0.10627400

Para-$PPhe_3$:

0 1

P	0.00389700	0.01100700	1.50680500
C	-0.02089800	-1.63542100	0.67339700
C	-0.89648700	-2.59930200	1.19191200
C	0.74535500	-1.96789100	-0.44989000
C	-1.02009800	-3.84934000	0.59050200
H	-1.49900400	-2.36378500	2.07363100
C	0.62612100	-3.22549900	-1.04146900
H	1.43585700	-1.23668200	-0.87617000
C	-0.25855800	-4.18569100	-0.53628600
H	-1.71877300	-4.58016900	1.00468100
H	1.22798400	-3.46388800	-1.92180200
C	1.45391000	0.80529600	0.69101200
C	1.36515100	1.72325600	-0.35950400
C	2.72757000	0.46143600	1.16998000
C	2.51972100	2.27576500	-0.91859300
H	0.38830200	2.01206100	-0.75301400
C	3.87460900	1.00254700	0.59868600
H	2.82236600	-0.24878300	1.99631500
C	3.79067100	1.92509600	-0.45440800
H	2.42738100	2.99206800	-1.73831900
H	4.85620200	0.71028800	0.97970000
C	-1.40388100	0.84779700	0.65711100
C	-1.91778600	2.01581000	1.23811100
C	-1.97964400	0.38818900	-0.53252500
C	-2.96556900	2.70937800	0.63752000
H	-1.49066900	2.38960900	2.17276000
C	-3.03556300	1.08086600	-1.12473700
H	-1.60057800	-0.51929600	-1.00737400
C	-3.54687700	2.25065400	-0.55226600
H	-3.34710400	3.62071700	1.10426900
H	-3.46920800	0.70425900	-2.05421400
C	5.03677800	2.53097100	-1.04399700
H	5.47522600	3.26711700	-0.35296900
H	5.80081600	1.76269400	-1.22817000
H	4.82188500	3.04368400	-1.99037900

C	-0.36657500	-5.54796500	-1.16851700
H	-0.16034800	-5.50246400	-2.24620400
H	0.36142700	-6.24307800	-0.72203800
H	-1.36645800	-5.97746400	-1.02111000
C	-4.70563200	2.98449200	-1.17334900
H	-4.84536700	2.69456900	-2.22272500
H	-5.63964100	2.75999600	-0.63541300
H	-4.55604000	4.07198800	-1.12735900

Para-In1(a):

0 1

P	0.93707400	-0.05303000	-0.05690100
C	0.39194800	-0.35568100	1.64081900
C	1.22291000	0.00219100	2.70634200
C	-0.88402400	-0.87138500	1.89657900
C	0.77463700	-0.15333400	4.01635200
H	2.22438400	0.38985600	2.51023600
C	-1.31919600	-1.02323700	3.20923700
H	-1.53875700	-1.16155800	1.07172900
C	-0.49837900	-0.66759500	4.28902700
H	1.42940200	0.12586900	4.84416100
C	0.18749400	-1.31259400	-1.12924500
C	-1.06045600	-1.08330500	-1.72238400
C	0.83151900	-2.54221600	-1.32262000
C	-1.64908700	-2.07217200	-2.50826500
H	-1.57876200	-0.13312200	-1.57832800
C	0.23304900	-3.51631900	-2.11501000
H	1.79625500	-2.70900200	-0.83472600
C	-1.01178700	-3.29954600	-2.72239800
H	0.74180600	-4.47147200	-2.26370600
C	0.23703300	1.53828500	-0.57213600
C	0.32816200	1.91342100	-1.91897500
C	-0.31474800	2.42982200	0.35443900
C	-0.12985000	3.16236400	-2.32807300
H	0.75723600	1.22832300	-2.65475000
C	-0.76914800	3.67717000	-0.06681800
H	-0.39178500	2.15267300	1.40768900
C	-0.68435600	4.06401700	-1.41018700
H	-1.19880800	4.36586800	0.66386600
H	-2.62265000	-1.88414400	-2.96563100
H	-0.05531900	3.44318000	-3.38040500
H	-2.31437500	-1.43022300	3.40109700
C	4.79301000	1.11482800	-0.57892000
C	5.45555200	-0.05001200	-0.16902300
C	4.73553500	-1.17119200	0.25102000
C	3.31994900	-1.23121100	0.30848900
C	2.73300400	-0.02753800	-0.13487500
C	3.40357000	1.13375600	-0.56553500
H	5.35553100	1.99358300	-0.89928400
H	5.32646300	-2.04508500	0.55765800
H	2.86192900	2.03187900	-0.87388500
H	6.54933700	-0.07188800	-0.17633500
C	-0.97883900	-0.85582900	5.70236100
C	-1.63138500	-4.35567700	-3.59646300
C	-1.18324000	5.41470700	-1.84667100
H	-2.26097100	5.51194700	-1.65041300
H	-1.01153200	5.57700000	-2.91789300
H	-0.67680900	6.21523600	-1.28843100
H	-1.15179400	-4.36264000	-4.58729900
H	-2.70377700	-4.17508700	-3.74302100
H	-1.49760400	-5.35547400	-3.16143700
H	-1.96255900	-0.38699900	5.84692400
H	-1.09126400	-1.92605200	5.93130000
H	-0.27547200	-0.42358900	6.42482100

Para-Product:

0 1

C	-2.67124600	0.45610200	-4.33815900
C	-1.37336500	0.14496500	-4.74794500
C	-0.34699400	0.00341200	-3.81280600
C	-0.62723600	0.18214100	-2.45274800
C	-1.93274200	0.47765300	-2.04785600
C	-2.95344400	0.61748500	-2.98267900
H	-3.46352300	0.56558700	-5.08023500
H	0.65677600	-0.24947000	-4.15227600
H	-3.95742300	0.84700500	-2.62268000
H	-1.15404100	0.00717100	-5.80742300
C	-2.17629500	0.60618900	-0.55872000
O	-3.29784700	0.82656900	-0.12537100
O	-1.09729500	0.45885000	0.10601800
P	0.62620700	-0.02830200	-1.13254800
C	1.29026800	1.44312500	-0.28746800
C	2.53811700	1.36292400	0.33418800
C	0.61103800	2.66558500	-0.31665800
C	3.08544300	2.48902600	0.94955400
H	3.09956300	0.42558700	0.33391900
C	1.18360300	3.78995100	0.26643500
H	-0.36331600	2.74124300	-0.80082400
C	2.42296400	3.71946800	0.91977300
C	2.13250400	-0.40515500	-2.16534600
C	2.67232300	-1.68799500	-2.31277300
C	2.73995500	0.65080700	-2.86196500
C	3.77762500	-1.90494300	-3.13633100
H	2.23914700	-2.53672200	-1.78238200
C	3.83863400	0.42880900	-3.68643800
H	2.34966700	1.66760600	-2.76377400
C	4.37879500	-0.85514300	-3.83782200
C	0.45790500	-1.56804300	-0.18663700
C	-0.13153200	-2.67509700	-0.80025100
C	0.90688900	-1.66198600	1.13333400
C	-0.26302500	-3.87114900	-0.09535400

H	-0.49123200	-2.61496900	-1.83059000
C	0.77539300	-2.86372600	1.82281000
H	1.34545200	-0.79591100	1.63256500
C	0.18850200	-3.98681600	1.22341600
H	-0.72647800	-4.73097300	-0.58345400
H	4.05345100	2.40905400	1.44806800
H	4.28958400	1.26822300	-4.22009900
H	0.65275000	4.74349500	0.22384300
H	4.18166300	-2.91540400	-3.23282800
H	1.13037100	-2.92996200	2.85367800
C	3.01485700	4.94556000	1.56054300
H	3.16373400	5.73991400	0.81460000
H	2.33973400	5.34546400	2.33095800
H	3.98208900	4.72528800	2.02914600
C	5.57965700	-1.10238400	-4.71058400
H	6.45807100	-1.35654200	-4.09850300
H	5.40279500	-1.94616400	-5.39219700
H	5.82775900	-0.21675700	-5.30914900
C	0.02917700	-5.26940600	1.99460800
H	0.95349100	-5.52411800	2.53143500
H	-0.76867400	-5.17075700	2.74629200
H	-0.23224500	-6.10377300	1.33170300

Para-TS2(b): (ν = 487.74i(cm^{-1}))

0 1

C	0.68650200	-1.08969500	4.68844400
C	-0.63940200	-1.11689000	4.21889700
C	-1.09221900	-0.39287000	3.11912800
C	-0.13148700	0.36439300	2.37146500
C	1.16669700	0.55821400	3.00982800
C	1.56713900	-0.15287900	4.12716500
C	1.21110300	1.98644700	2.64161300
O	2.01391900	2.88270400	2.69651900
O	-0.06182100	2.08773900	2.18850000
P	-0.19155700	0.09308400	0.52234600
C	1.02897000	1.10614000	-0.33572100
C	2.37509800	0.74375300	-0.21149400
C	0.68520400	2.28460700	-1.00963200
C	3.36623700	1.56141100	-0.75114900
C	1.68316000	3.08260500	-1.55418400
C	3.03828600	2.73944900	-1.42923900
C	-1.87531300	0.43320400	-0.03170700
C	-2.64300700	-0.53420500	-0.69365400
C	-2.44834000	1.67443400	0.28551400
C	-3.95625400	-0.24974400	-1.05355500
C	-3.76256800	1.94360600	-0.08687700
C	-4.53589200	0.99214800	-0.76273800
C	0.26662700	-1.61718700	0.16827200
C	0.66263300	-2.49428200	1.18478900
C	0.33296100	-2.03348300	-1.17048200
C	1.09418700	-3.77873800	0.85672500
C	0.75595500	-3.32026400	-1.48024000
C	1.13833500	-4.21640400	-0.47118800
H	0.97719200	-1.68801000	5.55091400
H	-2.13063300	-0.47029300	2.79094400
H	2.51197900	0.09358500	4.61931800
H	-1.36109700	-1.75692700	4.73377200
H	2.65520900	-0.17558400	0.30790700
H	-0.36015300	2.58071200	-1.11106700
H	-2.22557300	-1.51585900	-0.92119100
H	1.40829400	3.99583600	-2.08609400
H	4.41414400	1.27579300	-0.64272200
H	-1.87058400	2.41605900	0.84102400
H	-4.54618500	-1.01174400	-1.56713100
H	-4.19972400	2.91249300	0.16203700
H	0.65099900	-2.18502300	2.23117300
H	0.06500800	-1.34720300	-1.97804800
H	1.40849800	-4.45307600	1.65572900
H	0.80136700	-3.63366100	-2.52513100
C	-5.95546700	1.28136200	-1.16631200
H	-6.05818700	1.25426400	-2.26105200
H	-6.63865200	0.52376600	-0.75642000
H	-6.27875000	2.26850400	-0.81380800
C	4.10184600	3.61420300	-2.03401700
H	4.08620600	3.53448400	-3.13156800
H	3.93109900	4.66988600	-1.78138100
H	5.10163900	3.32646400	-1.68594200
C	1.57005500	-5.61662900	-0.80939900
H	2.26604100	-6.01049800	-0.05759400
H	0.69680700	-6.28665400	-0.83842900
H	2.05169100	-5.65766500	-1.79501900

NHMe:

Meta-PPhe$_3$:

0 1

P	0.35835700	0.05906900	-1.38552100
C	-0.37103600	1.37688200	-0.31847200
C	-1.37455400	2.15210300	-0.89678000
C	0.00328100	1.59861200	1.01522800
C	-2.05154600	3.15009000	-0.16298500
H	-1.65273500	1.98944900	-1.94261600
C	-0.65481800	2.58770300	1.74390600
H	0.79014400	1.00056000	1.47723300
C	-1.67346600	3.35364600	1.17894200
H	-0.37629200	2.76673600	2.78459000
C	1.86660400	-0.39591100	-0.42257600
C	1.90805400	-1.42630300	0.52837200
C	3.00975300	0.36101800	-0.67620000
C	3.10092900	-1.66929200	1.20721900
H	1.02157500	-2.02724600	0.73606400
C	4.21656900	0.13102300	0.01781300
C	4.24544200	-0.91185700	0.96490500

H	3.14717600	-2.47215200	1.94603900
C	-0.74980600	-1.36892600	-1.00993200
C	-0.52961200	-2.54797800	-1.74251300
C	-1.76703300	-1.32405300	-0.06178600
C	-1.31819000	-3.66541400	-1.48744900
H	0.25837300	-2.58914200	-2.49794300
C	-2.58509200	-2.44917500	0.18887900
H	-1.94852700	-0.40989300	0.50913800
C	-2.33630800	-3.63112200	-0.53408900
H	-1.14418000	-4.58913100	-2.04315700
H	-2.93962900	-4.52088700	-0.35605300
H	-2.17125800	4.11466100	1.77941000
H	5.16276400	-1.13338400	1.50969200
H	2.97939500	1.15604900	-1.42788400
C	6.62336400	0.58905200	0.27833500
H	7.34697900	1.31620500	-0.10715500
H	6.64815400	0.64313500	1.37813800
H	6.95641800	-0.42286400	-0.01654400
C	-4.32820000	-3.52033400	1.56173000
H	-3.66989900	-4.30467800	1.97823500
H	-5.03542400	-3.21363200	2.34067100
H	-4.90815400	-3.97110500	0.74093900
C	-3.87617900	4.79881000	-0.01762500
H	-4.61619700	5.23656800	-0.69706800
H	-4.41482200	4.29003200	0.80260800
H	-3.29632200	5.62484000	0.42367400
N	5.31552700	0.91455100	-0.23053300
H	5.27506200	1.49080000	-1.06042800
N	-3.02685400	3.90556600	-0.76304400
H	-3.38006600	3.56188600	-1.64605400
N	-3.59847300	-2.36359100	1.11026700
H	-3.57218400	-1.56451600	1.72929300

Meta-In1(a):

0 1

P	-0.18407600	-0.12027000	0.21655200
C	0.51868900	0.99543800	-1.02690700
C	1.28262700	2.07351400	-0.59089700
C	0.37567200	0.70399100	-2.38912900
C	1.93803400	2.90847400	-1.52149000
H	1.36390100	2.28860600	0.47728500
C	1.02108500	1.52898000	-3.30840400
H	-0.22951900	-0.14061300	-2.72116400
C	1.78971800	2.61359600	-2.89383300
H	0.91990400	1.32922900	-4.37653600
C	-1.65687300	-0.90286700	-0.50913700
C	-1.52412000	-2.11725300	-1.20202800
C	-2.88112300	-0.24665400	-0.42855500
C	-2.65780800	-2.65846400	-1.80400700
H	-0.56421500	-2.63009800	-1.26634400
C	-4.02972900	-0.80201800	-1.03465400
C	-3.89553200	-2.02468000	-1.72525600
H	-2.57979100	-3.60306500	-2.34523900
C	1.02295100	-1.45320700	0.47792900
C	0.65345800	-2.53759700	1.29075800
C	2.29227400	-1.37736900	-0.08439700
C	1.58994700	-3.54033200	1.52092200
H	-0.34515800	-2.59744800	1.72731700
C	3.24493600	-2.39685400	0.14898800
H	2.56867400	-0.52988300	-0.71598900
C	2.86658300	-3.48316100	0.96382300
H	1.32338600	-4.39376900	2.14682100
H	3.57376300	-4.28822300	1.16122800
H	2.27359300	3.24173100	-3.64116900
H	-4.76175900	-2.48145200	-2.20333800
H	-2.93215100	0.70663000	0.10810600
C	-0.09412900	1.16559400	4.07211200
C	-0.99522400	2.23549400	3.99344800
C	-1.62739300	2.54790100	2.78720600
C	-1.41674200	1.84659900	1.57306100
C	-0.51137700	0.77718400	1.74208400
C	0.15630400	0.41640200	2.92843300
H	0.40497700	0.92300000	5.01192600
H	-2.31866200	3.40172400	2.80466300
H	0.85609900	-0.42245300	2.96313300
H	-1.19888800	2.82974700	4.88937500
C	3.26289100	4.93488000	-1.98428500
H	3.78577300	5.69643200	-1.39549600
H	3.99522300	4.47688200	-2.66816900
H	2.49512100	5.43834200	-2.59821400
C	5.48110800	-3.34524800	-0.27313400
H	5.12898900	-4.31224400	-0.67293100
H	6.37760500	-3.05094200	-0.82953400
H	5.76898600	-3.50084500	0.77971700
C	-6.45686300	-0.68880500	-1.46453400
H	-7.26833400	0.01782400	-1.25856700
H	-6.40966100	-0.84056800	-2.55540200
H	-6.71542300	-1.65683800	-1.00000400
N	-5.22518100	-0.14864900	-0.95306200
N	4.48836200	-2.31133600	-0.40366900
N	2.69751600	3.95834800	-1.08907300
H	-5.26706500	0.66780900	-0.35942900
H	2.63299700	4.19820200	-0.10921900
H	4.66700800	-1.54916100	-1.04203700

Meta-Product:

0 1

C	-2.60627900	0.40114400	-4.22530500
C	-1.31093300	0.09567800	-4.64701700
C	-0.27615900	-0.04277000	-3.72084700
C	-0.54407700	0.13330800	-2.35771500
C	-1.84734300	0.42289600	-1.93997400
C	-2.87597400	0.55975700	-2.86705800
H	-3.40590100	0.50815900	-4.95990700

H	0.72665600	-0.28965400	-4.06782000
H	-3.87738800	0.78454300	-2.49698000
H	-1.10017000	-0.04002000	-5.70855300
C	-2.08645600	0.54899700	-0.44774500
O	-3.21170900	0.76742400	-0.01753200
O	-1.01020900	0.40428500	0.21547900
P	0.73231900	-0.07875600	-1.05985800
C	1.39435500	1.40540300	-0.22970500
C	2.64974600	1.32721200	0.38791800
C	0.70177300	2.60801200	-0.29507200
C	3.17389000	2.47875000	0.97079400
H	3.21821500	0.39623700	0.39565000
C	1.24652400	3.78332800	0.26462000
H	-0.27581400	2.65644500	-0.77816700
C	2.49351000	3.69258000	0.91629900
C	2.21783400	-0.47051200	-2.11406800
C	2.67248100	-1.78124200	-2.30947800
C	2.84191200	0.58641400	-2.77953000
C	3.74242700	-2.00347200	-3.17857100
H	2.20754200	-2.62142500	-1.79508600
C	3.92269100	0.37035300	-3.65859800
H	2.49805200	1.61360500	-2.62569600
C	4.36194700	-0.95689500	-3.85270700
C	0.56052700	-1.61723400	-0.10666500
C	-0.17250300	-2.65156500	-0.67810900
C	1.17396000	-1.76279600	1.14155500
C	-0.31947600	-3.88382300	-0.00466200
H	-0.64672100	-2.52606300	-1.65533900
C	1.03074700	-2.98072600	1.80452100
H	1.72685700	-0.94382100	1.60092100
C	0.30054600	-4.03021600	1.25388500
H	4.14120800	2.43389700	1.47400900
H	4.10412600	-3.02153500	-3.33495100
H	1.49381900	-3.11515700	2.78385900
H	5.19263300	-1.16606900	-4.52630500
H	0.20594600	-4.96540100	1.80459000
H	2.93512000	4.57553500	1.37711000
N	4.52718100	1.42672000	-4.28274700
H	4.06657500	2.32373500	-4.21458400
N	0.56950600	4.96862900	0.16052700
H	-0.39627200	4.91103200	-0.13347700
N	-1.03177800	-4.89913800	-0.58004400
H	-1.59041000	-4.66239400	-1.38863900
C	-1.35632600	-6.11576400	0.11989900
H	-1.91081200	-5.92946400	1.05689400
H	-1.97714300	-6.74378700	-0.52856300
H	-0.44871100	-6.68666200	0.37234800
C	5.53431300	1.26362800	-5.29860400
H	5.17915300	0.66394600	-6.15576800
H	5.82881200	2.25165200	-5.66958200
H	6.43546200	0.77398700	-4.89581400
C	0.97332700	6.15489600	0.87176100
H	1.95300000	6.51638000	0.52129800
H	0.23899900	6.94695600	0.68753000
H	1.04251400	5.99251000	1.96234100

Meta-TS2(b): (ν = 488.90i(cm^{-1}))

0 1

C	0.58212800	-1.05074900	4.69115000
C	-0.73320900	-1.01782400	4.19481600
C	-1.12881200	-0.28224600	3.08002200
C	-0.11962300	0.42175900	2.34296300
C	1.17362100	0.55613600	3.00733700
C	1.51665900	-0.16113500	4.13880800
C	1.29080100	1.98035400	2.63977800
O	2.13358400	2.83809100	2.72100100
O	0.03723100	2.13908600	2.15740800
P	-0.17565800	0.13176200	0.49525800
C	1.04357900	1.12573300	-0.39139100
C	2.38224600	0.81602000	-0.15654600
C	0.67506100	2.19933300	-1.21173800
C	3.40851400	1.60424700	-0.71996100
C	1.68947000	2.96292700	-1.78371300
C	3.03331300	2.68749900	-1.54166400
C	-1.85974000	0.49049800	-0.05226200
C	-2.63964800	-0.49744500	-0.64775600
C	-2.37786000	1.76900600	0.21730500
C	-3.97716500	-0.22778300	-1.01700100
C	-3.69380100	2.03139700	-0.15312200
C	-4.48792600	1.06107100	-0.76019900
C	0.25839200	-1.59170600	0.15469400
C	0.57192600	-2.48247500	1.18897400
C	0.34860200	-1.97509700	-1.18492100
C	0.96377900	-3.77591400	0.83975700
C	0.73351600	-3.28516100	-1.53491200
C	1.04224800	-4.18175600	-0.48750600
H	0.82763700	-1.65311300	5.56481700
H	-2.16398000	-0.31052100	2.73404700
H	2.46094800	0.04911200	4.64886300
H	-1.49613400	-1.61614600	4.70056600
H	2.65256100	-0.03628600	0.47280200
H	-0.37259400	2.43391900	-1.39742300
H	-2.23207000	-1.49321300	-0.83233200
H	1.42954200	3.80208500	-2.43113900
H	-1.76810400	2.51970200	0.72115500
H	-4.12036500	3.01674500	0.04219000
H	0.52627300	-2.18361600	2.23575200
H	0.13111300	-1.25773100	-1.98162100
H	1.21924400	-4.48684200	1.62714800

H	-5.51585500	1.30274100	-1.02893700
H	3.79788700	3.31589500	-1.99711500
H	1.35387100	-5.20009500	-0.71830900
N	0.79756200	-3.66196600	-2.84497000
H	0.70106100	-2.93561600	-3.54098500
N	-4.73318900	-1.19700100	-1.60935200
H	-4.34764800	-2.13046500	-1.64409600
N	4.71820400	1.29937200	-0.47965700
H	4.90173400	0.62832300	0.25391500
C	5.80326600	2.15483900	-0.88861500
H	6.74634400	1.72864500	-0.52923300
H	5.86283000	2.22823900	-1.98605000
H	5.70827200	3.17814700	-0.48421100
C	-6.13553200	-1.03194800	-1.89361200
H	-6.30551100	-0.22893700	-2.62881800
H	-6.52451600	-1.96422600	-2.31739800
H	-6.72088100	-0.79224100	-0.98859600
C	1.30288100	-4.94241500	-3.26985400
H	1.28356500	-4.98553600	-4.36418600
H	2.34022500	-5.11782600	-2.93459100
H	0.67949500	-5.76692700	-2.88850700

Para-PPhe$_3$:

0 1

P	-0.04807300	-0.16789000	1.76934300
C	-0.01519600	1.51811200	1.02790700
C	0.83579000	2.47523300	1.60819200
C	-0.75889400	1.90983600	-0.08891600
C	0.95683000	3.75277200	1.08517900
H	1.42769400	2.20757500	2.48863600
C	-0.65736500	3.19442000	-0.62357100
H	-1.43437400	1.19581100	-0.56625300
C	0.20786200	4.14491100	-0.04777400
H	1.63146400	4.47417200	1.55191600
H	-1.25391900	3.45523100	-1.49769500
C	-1.41755800	-0.95725900	0.82756300
C	-1.25409600	-1.63432700	-0.39156300
C	-2.71661100	-0.85005300	1.33818300
C	-2.33858000	-2.17097700	-1.07032800
H	-0.25500600	-1.74152400	-0.82120900
C	-3.81929000	-1.36824000	0.66335600
H	-2.87890600	-0.33747400	2.29096500
C	-3.65017200	-2.04751800	-0.56040500
H	-2.18433300	-2.69654500	-2.01565100
H	-4.81372200	-1.25233700	1.09401600
C	1.43649000	-0.92289800	0.98603700
C	1.94885400	-2.11336000	1.53076000
C	2.09181200	-0.39040900	-0.12913200
C	3.05153700	-2.74870500	0.98064800
H	1.46924100	-2.55477200	2.40923400
C	3.20572900	-1.01042400	-0.69189400
H	1.72463700	0.53581800	-0.57814900
C	3.71182400	-2.20736900	-0.14549400
H	3.42939600	-3.67265400	1.42443200
H	3.68446600	-0.55849200	-1.56044600
C	-0.27647800	5.82030100	-1.78951100
H	-1.37566500	5.76410400	-1.74458300
H	-0.00374000	6.86142600	-1.99618500
H	0.05715900	5.19819700	-2.63989600
C	-6.07790900	-2.34491400	-0.86621300
H	-6.32412200	-1.26769100	-0.85249500
H	-6.73808100	-2.83995600	-1.58725200
H	-6.30362800	-2.75394700	0.13132600
C	5.45567700	-2.40318600	-1.87932500
H	5.86634000	-1.38516500	-1.78320100
H	6.28796900	-3.08090900	-2.10002500
H	4.76715700	-2.40805900	-2.74323800
N	-4.70843300	-2.59584900	-1.23520600
H	-4.53048300	-2.89672900	-2.18387100
N	0.31789000	5.41847000	-0.54087900
H	1.08846500	5.97253500	-0.19281100
N	4.81672800	-2.82711600	-0.66124200
H	5.02478000	-3.75398400	-0.31620600

Para-In1(a):

0 1

P	0.98838200	-0.13004000	-0.11396200
C	1.13261000	-0.14627200	1.67948300
C	2.34619800	0.17375700	2.29835200
C	0.00908600	-0.39491700	2.48454700
C	2.44642900	0.25076100	3.68150800
H	3.23236400	0.35373300	1.68599200
C	0.09538800	-0.32255300	3.86343000
H	-0.94760400	-0.65503400	2.02415100
C	1.31852100	0.00496100	4.49754200
H	3.40672000	0.49435200	4.13441600
C	-0.22525500	-1.37025600	-0.61677600
C	-1.58477000	-1.03459200	-0.74920700
C	0.15795700	-2.70373300	-0.81771900
C	-2.52451500	-1.99411300	-1.08303800
H	-1.91488500	-0.00503600	-0.59467900
C	-0.77704700	-3.67284500	-1.15554700
H	1.21424100	-2.96269600	-0.69872300
C	-2.14356100	-3.34015100	-1.29765200
H	-0.44571200	-4.69963000	-1.30788900
C	0.30772800	1.47269400	-0.58841600

C	-0.11264300	1.68657700	-1.90970200
C	0.24970200	2.54766000	0.31230300
C	-0.57910600	2.92459500	-2.32735500
H	-0.07656900	0.86676700	-2.63254400
C	-0.21475000	3.78868900	-0.09027200
H	0.57052100	2.40962400	1.34729600
C	-0.64042800	4.00965200	-1.42118100
H	-0.25757100	4.61206800	0.62511800
H	-3.57471900	-1.71431300	-1.18617500
H	-0.89904100	3.05387400	-3.36048300
H	-0.78472600	-0.52594900	4.47646500
C	4.40370300	0.37324000	-2.26350500
C	5.07933700	-0.81365600	-1.95434500
C	4.50100200	-1.75915300	-1.10269700
C	3.23073700	-1.61143800	-0.48894600
C	2.61119600	-0.39773400	-0.86186000
C	3.14647400	0.59032000	-1.71020300
H	4.85301500	1.11700500	-2.92427700
H	5.09223800	-2.66433800	-0.90478500
H	2.60257500	1.51209400	-1.93270600
H	6.06966300	-0.99253300	-2.38444700
N	-1.09489700	5.23028600	-1.80554000
H	-1.11186200	5.96537100	-1.11236600
N	-3.07356800	-4.27265300	-1.63176900
H	-4.03966700	-3.97971400	-1.67397400
N	1.39240400	0.07380300	5.85245500
H	0.55514500	-0.12718800	6.38122400
C	-1.54162800	5.53351500	-3.14109600
H	-1.85342500	6.58272400	-3.18129400
H	-0.74184100	5.38486700	-3.88538400
H	-2.40109500	4.90920400	-3.43686600
C	-2.76840200	-5.66600500	-1.83218600
H	-2.34240400	-6.12950200	-0.92631200
H	-3.69047500	-6.19893000	-2.08797600
H	-2.05006500	-5.81040200	-2.65562600
C	2.59991200	0.39172400	6.57021200
H	2.98356300	1.38988500	6.30140300
H	2.38672100	0.38687100	7.64451800
H	3.39923600	-0.34270700	6.37537600

Para-Product:

0 1

C	-2.32880600	0.78701700	-4.57724300
C	-0.96678100	0.83385200	-4.86892000
C	-0.03052200	0.60040100	-3.86255900
C	-0.44626500	0.33637500	-2.54791500
C	-1.81932800	0.35004500	-2.24388900
C	-2.74388800	0.54748900	-3.26959800
H	-3.06496100	0.95229400	-5.36578000
H	1.02876700	0.63193700	-4.11377300
H	-3.79963300	0.52768600	-2.99606700
H	-0.62413000	1.04653100	-5.88235200
C	-2.33158200	0.27163000	-0.79861500
O	-3.53570000	0.05063100	-0.62985200
O	-1.44334400	0.49137300	0.05555800
P	0.83651400	-0.01470300	-1.28203300
C	1.23213900	1.42840700	-0.29074300
C	2.16073900	1.34092400	0.75882100
C	0.62459400	2.65923400	-0.55223300
C	2.47987600	2.45491500	1.51562500
H	2.64273400	0.38811200	0.99265400
C	0.93897300	3.78511400	0.19666900
H	-0.12233200	2.73879000	-1.34484900
C	1.87648000	3.70788800	1.25168600
C	2.33684600	-0.39386700	-2.25054900
C	2.32353000	-1.37613900	-3.25539800
C	3.55401500	0.26710300	-2.01235100
C	3.46486100	-1.71421300	-3.96751800
H	1.39180500	-1.87658400	-3.52791600
C	4.70341900	-0.05864000	-2.71393600
H	3.61282000	1.06348100	-1.27025900
C	4.69445600	-1.06792500	-3.70250800
C	0.56970100	-1.47143200	-0.24075900
C	0.29386000	-1.33847000	1.12774900
C	0.66514200	-2.76839200	-0.77206600
C	0.14201500	-2.44986200	1.94355000
H	0.17151300	-0.34442300	1.55436100
C	0.49037100	-3.88545500	0.02581900
H	0.87624600	-2.92886000	-1.82849100
C	0.23116000	-3.75541400	1.40978200
H	-0.06500700	-2.30450300	3.00311300
H	3.20180500	2.37009300	2.32989500
H	5.63261200	0.47470200	-2.50474700
H	0.44367000	4.72867400	-0.02829000
H	3.39938700	-2.47667200	-4.74263200
H	0.56122500	-4.88192700	-0.41410600
N	2.19840200	4.79625600	1.99949400
H	2.82324200	4.65942300	2.78175700
N	5.82862000	-1.38989800	-4.37570400
H	6.66619700	-0.86597000	-4.16289200
N	0.07792000	-4.85674000	2.18949700
H	0.11548100	-5.76088200	1.73988100
C	1.57771200	6.08577200	1.83497500
H	0.48635900	6.04193200	1.99009200
H	2.00059800	6.78051700	2.56862200
H	1.76059800	6.49845800	0.82949400
C	-0.21500200	-4.80109800	3.59880500
H	-1.16785200	-4.28401600	3.80179900
H	-0.29102500	-5.82266500	3.98644700
H	0.57935100	-4.28076400	4.15866000
C	5.88442300	-2.38626700	-5.41478500

H	6.91353900	-2.45921900	-5.78234300
H	5.58314500	-3.37880200	-5.04182700
H	5.23231200	-2.13063500	-6.26685300

Para-TS2(b): (ν = 487.56i(cm^{-1}))

0 1

C	0.61714700	-1.07588700	4.72768200
C	-0.70446600	-1.09466300	4.24245200
C	-1.14174000	-0.36394500	3.14224200
C	-0.17020800	0.39202600	2.40238000
C	1.12307500	0.57695100	3.05930700
C	1.50610100	-0.13828300	4.17993100
C	1.16831700	2.00991700	2.71168700
O	1.97162500	2.90648600	2.78574700
O	-0.10127800	2.11897900	2.25701100
P	-0.20549400	0.13301200	0.55354500
C	1.03370300	1.13648900	-0.27012900
C	2.38381800	0.75751900	-0.15837400
C	0.72551400	2.34415500	-0.90782100
C	3.38969500	1.56199800	-0.66179500
C	1.72469000	3.15417200	-1.42645600
C	3.08512900	2.78447200	-1.30677000
C	-1.86980700	0.49547900	-0.01559800
C	-2.65117700	-0.45874300	-0.69045200
C	-2.44896200	1.73942300	0.29112600
C	-3.95013300	-0.17304600	-1.06973200
C	-3.74933900	2.03622200	-0.08938400
C	-4.53232200	1.08557200	-0.78545000
C	0.23553800	-1.57447100	0.21750300
C	0.63025900	-2.45991300	1.22939400
C	0.31079100	-2.00846900	-1.12148000
C	1.05941300	-3.74647000	0.92476900
C	0.72503400	-3.28774200	-1.43393600
C	1.10620300	-4.19553100	-0.41343700
H	0.89581300	-1.67791200	5.59168800
H	-2.17675100	-0.43478100	2.80173300
H	2.44449600	0.10601000	4.68597100
H	-1.43439600	-1.73430200	4.74671000
H	2.64797200	-0.18331600	0.33074000
H	-0.31410100	2.66255100	-1.00614200
H	-2.24383700	-1.44619300	-0.91361900
H	1.45216300	4.08239100	-1.92704600
H	4.43274000	1.25499200	-0.56583600
H	-1.87473500	2.47702700	0.85624600
H	-4.54164400	-0.92851000	-1.59027400
H	-4.16705700	3.00974400	0.16429800
H	0.61820100	-2.15083100	2.27617600
H	0.04758400	-1.32704900	-1.93510600
H	1.36404400	-4.40733600	1.73512700
H	0.77078100	-3.60713000	-2.47675900
N	-5.80597100	1.35544700	-1.16535500
H	-6.31841300	0.62717100	-1.64316200
N	1.50783600	-5.45162900	-0.73076600
H	1.55386100	-5.69819800	-1.70980800
N	4.07865800	3.56591900	-1.80147400
H	5.03164000	3.26453900	-1.65259900
C	-6.47473400	2.60264700	-0.89344700
H	-7.49098900	2.55563600	-1.29907300
H	-6.54517400	2.80202600	0.18858400
H	-5.95634900	3.45420200	-1.36384800
C	3.85320100	4.83725300	-2.44026200
H	4.81883700	5.26244000	-2.73418300
H	3.23541600	4.73362100	-3.34729500
H	3.35227900	5.55323700	-1.76729500
C	1.95322300	-6.41579000	0.24311800
H	1.16546100	-6.63786200	0.98113200
H	2.21108900	-7.34730600	-0.27190300
H	2.84367800	-6.06593100	0.79187700

NO_2:

Meta-PPhe$_3$:

0 1

P	-0.08464000	-0.78529300	1.62223800
C	1.46736900	-1.46443400	0.88586500
C	1.70673700	-2.83746500	1.05419300
C	2.40566700	-0.69210700	0.19835500
C	2.84700600	-3.43995900	0.52524300
H	0.98734600	-3.44634300	1.60756000
C	3.54039100	-1.31650900	-0.30885100
H	2.27675300	0.37875700	0.04301600
C	3.78429000	-2.67681000	-0.16774500
H	3.01216300	-4.50886900	0.66124700
H	4.68885400	-3.11308600	-0.58762700
C	0.15297000	1.02159700	1.32270800
C	-0.29169900	1.68454700	0.17633600
C	0.82237200	1.75130700	2.31682800
C	-0.04606700	3.04795000	0.05857900
H	-0.81797900	1.16869500	-0.62677000
C	1.06585500	3.11620200	2.16763500
H	1.15941800	1.24192200	3.22304300
C	0.62658200	3.78364200	1.02697800
H	1.59223000	3.66495200	2.94872700
H	0.79089900	4.84984300	0.88187100
C	-1.30750600	-1.19468700	0.29926100
C	-2.65729800	-1.03767200	0.63153500
C	-0.96987000	-1.63864800	-0.98623300
C	-3.62023100	-1.30271400	-0.33360400
H	-2.97032700	-0.71135400	1.62421500
C	-1.95951100	-1.91095700	-1.93285300
H	0.07839000	-1.77180200	-1.26014500
C	-3.30345800	-1.73854200	-1.61554700
H	-1.67803200	-2.25423500	-2.92853700

H	-4.09736900	-1.93578200	-2.33370700
N	-5.04133800	-1.12349700	0.02252200
O	-5.86224800	-1.29809500	-0.84570000
O	-5.30007900	-0.81257800	1.16073800
N	-0.52026300	3.74461200	-1.15409700
O	-1.11147300	3.09334500	-1.98212400
O	-0.28935300	4.92627900	-1.24691300
N	4.52664700	-0.49299300	-1.03619100
O	4.33241100	0.69817400	-1.09034200
O	5.46953500	-1.05820000	-1.53618200

Meta-In1(a):

0 1

P	-0.13210700	-0.06708500	0.21535700
C	0.55356700	1.07888700	-1.01318700
C	1.17365400	2.24934500	-0.57262400
C	0.51157400	0.76687300	-2.37693600
C	1.74608500	3.07763100	-1.52738800
H	1.20054800	2.52359600	0.48200200
C	1.09586100	1.62360800	-3.31023300
H	0.01840300	-0.14482600	-2.72172100
C	1.72196000	2.79308100	-2.88891500
H	1.05906300	1.37885800	-4.37144200
C	-1.62585900	-0.81347900	-0.51398000
C	-1.55445500	-2.02417600	-1.21611800
C	-2.84246200	-0.13427400	-0.39874400
C	-2.70073000	-2.56517000	-1.80035000
H	-0.60660000	-2.55734100	-1.31193700
C	-3.96019200	-0.70551500	-0.98792100
C	-3.92134900	-1.90650900	-1.68888300
H	-2.63904100	-3.50763400	-2.34394200
C	1.06735400	-1.42033400	0.40081000
C	0.71438200	-2.52802500	1.18879100
C	2.33988500	-1.33966700	-0.16414100
C	1.62752500	-3.55663700	1.40561800
H	-0.28086300	-2.59057900	1.63624000
C	3.22647900	-2.38393900	0.07125400
H	2.65579400	-0.49615700	-0.77797200
C	2.90124000	-3.49178500	0.84258900
H	1.34713800	-4.41435600	2.01655000
H	3.63733500	-4.28002500	0.99187100
H	2.18361100	3.48376500	-3.59266500
H	-4.83243000	-2.30479300	-2.13259500
H	-2.88532600	0.81746300	0.14266300
C	-0.06648500	1.14510400	4.08840900
C	-0.97289400	2.21230800	3.99626100
C	-1.58512000	2.53471100	2.78370200
C	-1.34531200	1.83632000	1.57384800
C	-0.43793500	0.77662000	1.75851900
C	0.21472200	0.40300600	2.94959400
H	0.41332100	0.90281500	5.03783900
H	-2.27953400	3.38428400	2.79073800
H	0.92170600	-0.42960800	2.99191500
H	-1.19533600	2.79860200	4.89234600
N	2.40763500	4.31779500	-1.07374000
O	2.89152700	5.03236000	-1.91771100
O	2.42522300	4.53967500	0.11254000
N	4.57350100	-2.30847500	-0.53017200
O	5.33072800	-3.22357600	-0.31736900
O	4.83354200	-1.33684500	-1.19752600
N	-5.25248300	0.00162700	-0.86746300
O	-6.21123400	-0.49397900	-1.40941100
O	-5.27138700	1.02850200	-0.23463900

Meta-Product:

0 1

C	-2.69884400	0.36285500	-4.43087800
C	-1.39913200	0.03092000	-4.82026600
C	-0.37407000	-0.07449300	-3.87861400
C	-0.66440600	0.16334600	-2.53091000
C	-1.97097900	0.47559600	-2.14902300
C	-2.99245700	0.58048700	-3.08538800
H	-3.48543500	0.44414000	-5.18221300
H	0.62728900	-0.34703900	-4.20829600
H	-3.99918200	0.82588100	-2.74460900
H	-1.17686300	-0.15137200	-5.87223800
C	-2.18204200	0.64648400	-0.66977800
O	-3.26542900	0.88499700	-0.18155900
O	-1.06352200	0.50254900	-0.03012700
P	0.51587000	0.01450800	-1.13532900
C	1.28136200	1.45827500	-0.29501700
C	2.55707500	1.32275300	0.26513500
C	0.62572300	2.68847400	-0.24591400
C	3.17097800	2.40381700	0.89843700
H	3.08864400	0.37044200	0.20646000
C	1.28118400	3.75538200	0.35338500
H	-0.36920800	2.83675500	-0.66215200
C	2.53720900	3.64113000	0.93958700
C	2.06200200	-0.37549500	-2.15936600
C	2.67985800	-1.62862300	-2.22901900
C	2.61125800	0.67812000	-2.90390300
C	3.81040900	-1.83796200	-3.02721100
H	2.29408600	-2.46982400	-1.65285300
C	3.72759900	0.44592100	-3.69212800
H	2.18514400	1.68330400	-2.88616800
C	4.34718300	-0.79813700	-3.77450200
C	0.41225500	-1.54150800	-0.18811800
C	-0.12277800	-2.67897300	-0.80228500
C	0.83092300	-1.59009600	1.14054400
C	-0.23270000	-3.87464100	-0.09210400
H	-0.45754000	-2.64072100	-1.84172600
C	0.71489900	-2.79863100	1.81572200
H	1.22796000	-0.72060500	1.66467600
C	0.19206100	-3.94636500	1.23169500

H	-0.65202200	-4.75649900	-0.57610400
H	4.15565700	2.28112900	1.34896500
H	4.27341600	-2.82428100	-3.06074500
N	4.28552400	1.56788600	-4.47361300
O	5.24435200	1.33883300	-5.17108200
O	3.75114900	2.64594200	-4.36995800
N	1.16181400	-2.85925900	3.22228000
O	1.02271100	-3.90592400	3.80712200
O	1.63925900	-1.85898700	3.70125600
N	0.60479000	5.06772900	0.38082500
O	1.17034000	5.97430700	0.94266700
O	-0.47100300	5.15585300	-0.15993500
H	2.99627500	4.50898700	1.40974500
H	0.12308900	-4.86546400	1.81081000
H	5.22437800	-0.92834800	-4.40572100

Meta-TS2(b): (ν = 488.37i(cm^{-1}))

0 1

C	-1.81038800	-1.62054900	4.02378400
C	-0.47533400	-1.98658300	3.77474800
C	0.07337200	-2.11196000	2.49984600
C	-0.75835700	-1.77953800	1.38539000
C	-2.18017700	-1.63396000	1.65561200
C	-2.69296000	-1.53920100	2.93860300
C	-2.56224100	-2.37261000	0.43678300
O	-3.57282100	-2.60477700	-0.17193800
O	-1.31752500	-2.80857100	0.10606600
P	0.01734300	-0.61699700	0.12758000
C	-1.16402800	-0.23115600	-1.18413800
C	-2.27280200	0.53853700	-0.82019400
C	-1.07516400	-0.78607400	-2.46673600
C	-3.27991700	0.71259700	-1.76100500
C	-2.09588600	-0.57577000	-3.38973100
C	-3.21973900	0.17028600	-3.03775400
C	1.53465500	-1.35592500	-0.52403200
C	2.76954500	-0.72168400	-0.36912100
C	1.46146000	-2.62700800	-1.12154300
C	3.89925500	-1.36860200	-0.85243000
C	2.61718000	-3.24034100	-1.59945800
C	3.85342900	-2.61015500	-1.47296600
C	0.38604000	0.97045000	0.91684100
C	0.04024100	1.21289000	2.25328900
C	0.93970600	1.99106700	0.13489600
C	0.27555700	2.46901200	2.81599500
C	1.16759000	3.22160200	0.73130600
C	0.85040300	3.48500100	2.06000200
H	-2.17150600	-1.52657500	5.04685900
H	1.12467200	-2.37579900	2.37033200
H	-3.77430800	-1.50588800	3.09333700
H	0.18443300	-2.16659800	4.62746000
H	-2.37621600	0.99230300	0.16620200
H	-0.20943400	-1.38499500	-2.75254400
H	2.88393300	0.24680800	0.11657700
H	-2.01910100	-1.00248000	-4.38936200
H	0.49876000	-3.13732100	-1.19103000
H	2.55310200	-4.22275000	-2.06662800
H	-0.41945000	0.43332700	2.86296200
H	1.18798700	1.85641200	-0.91914000
H	0.00433000	2.65322000	3.85508400
H	4.77317200	-3.06723500	-1.83481100
H	-4.03761500	0.33928100	-3.73612100
H	1.04581000	4.47211800	2.47623800
N	5.20743600	-0.70126900	-0.69133800
O	6.17096500	-1.24200900	-1.17520100
O	5.22981500	0.34210100	-0.08456300
N	1.76079500	4.29791400	-0.08848600
O	1.94506200	5.36440700	0.44369000
O	2.02377000	4.04180100	-1.23844200
N	-4.46330900	1.50702700	-1.37844500
O	-5.33076900	1.64876200	-2.20498500
O	-4.48997500	1.96622200	-0.26202000

Para-PPhe$_3$:

0 1

P	-0.00585500	-0.01061400	1.74238900
C	-0.40111100	-1.60155600	0.89250500
C	-1.51272100	-2.30818800	1.37842400
C	0.34512200	-2.13500300	-0.16582100
C	-1.89082400	-3.51850200	0.80900700
H	-2.09278100	-1.90772600	2.21310900
C	-0.01171500	-3.35446800	-0.73714300
H	1.21201900	-1.60207600	-0.55881300
C	-1.12492300	-4.01881200	-0.23878400
H	-2.75365700	-4.07797100	1.16646200
C	1.57913700	0.45049000	0.91558700
C	1.66877500	1.30788500	-0.18816900
C	2.75024700	-0.10041900	1.45855600
C	2.90665400	1.60616800	-0.75259000
H	0.77005400	1.74861300	-0.62197100
C	3.99275100	0.17808000	0.89961300
H	2.69334200	-0.75694700	2.32973800
C	4.04312400	1.03067500	-0.19803500
H	4.91146200	-0.24362900	1.30375300
C	-1.18976000	1.13974500	0.91353600
C	-1.32487300	2.41463300	1.48394200
C	-1.94190000	0.80916400	-0.22123100
C	-2.18071000	3.35743400	0.92434200
H	-0.75396400	2.67632300	2.37763500
C	-2.81180600	1.73810800	-0.78722500
H	-1.85096700	-0.17750000	-0.67830000
C	-2.90990700	2.99505800	-0.20309100
H	-3.40299200	1.50093500	-1.66999600

H	2.99648000	2.26928600	-1.61123200
H	-2.29547500	4.35258700	1.35061600
H	0.55437900	-3.78775300	-1.56000800
N	-3.82707500	3.98359200	-0.80086400
O	-4.44639800	3.65251200	-1.78363300
O	-3.90562600	5.06716300	-0.27282600
N	5.35632100	1.33882300	-0.79541500
O	5.37598600	2.05802400	-1.76548600
O	6.33439800	0.85184800	-0.28044200
N	-1.51006300	-5.30805000	-0.84343500
O	-0.82327000	-5.73362400	-1.74136900
O	-2.48966500	-5.86208100	-0.40438200

Para-In1(a):

0 1

P	-0.48975300	1.07115600	3.06266600
C	-1.21280400	-0.20281600	1.98811100
C	-1.83007300	-1.31304400	2.57558800
C	-1.20691500	-0.03684400	0.59909000
C	-2.45575900	-2.26014400	1.77176900
H	-1.80910200	-1.44315100	3.65850500
C	-1.82782500	-0.98179600	-0.21365200
H	-0.71640300	0.82280000	0.13861900
C	-2.44022100	-2.07015200	0.39440900
H	-2.94500400	-3.13478900	2.19620600
C	0.90252300	1.81927600	2.14937000
C	0.69704800	2.96939100	1.37508900
C	2.16012000	1.20466000	2.19756700
C	1.75369400	3.51620900	0.65231700
H	-0.28107700	3.45023300	1.32600200
C	3.22215000	1.75188800	1.48391900
H	2.27742500	0.29826900	2.79980200
C	2.99338800	2.89473300	0.72725000
H	4.21377700	1.30328000	1.50605100
C	-1.73311400	2.39130200	3.22932500
C	-1.36688400	3.55331800	3.92594500
C	-3.02960400	2.24517400	2.72690100
C	-2.29335500	4.57128100	4.11769500
H	-0.35499500	3.67117200	4.32073000
C	-3.96488500	3.26205000	2.91041500
H	-3.32064200	1.34397300	2.18562200
C	-3.57380000	4.40007400	3.60145700
H	-4.97951900	3.17890500	2.52531600
H	1.62569800	4.40777700	0.04130200
H	-2.03858300	5.48335100	4.65431000
H	-1.83899200	-0.88430600	-1.29754200
N	-4.56280800	5.48243400	3.79549000
O	-5.65351400	5.33523000	3.30090600
O	-4.21729800	6.44509300	4.43571500
N	4.11837600	3.47501600	-0.03828300
O	3.89080300	4.45444300	-0.70607500
O	5.19291900	2.93366900	0.05248700
N	-3.10480100	-3.07463000	-0.46370900
O	-3.07400700	-2.89631200	-1.65709500
O	-3.63983000	-4.00807900	0.08256100
C	0.73524300	-0.84584400	6.93748500
C	-0.18832600	0.20753600	7.01807500
C	-0.58754500	0.84124100	5.84956800
C	-0.02940100	0.37840300	4.64172200
C	0.88766200	-0.67429600	4.46821100
C	1.24924100	-1.26022900	5.70729700
H	1.04841400	-1.34813800	7.85718900
H	-1.31242800	1.65840500	5.88378900
H	1.96426000	-2.09258100	5.72581100
H	-0.59006000	0.52331100	7.98212000

Para-Product:

0 1

C	-2.70472300	0.40331000	-4.38814500
C	-1.41083800	0.06130900	-4.78850400
C	-0.38122300	-0.06283400	-3.85417100
C	-0.66193600	0.16494700	-2.50279100
C	-1.96293700	0.48658900	-2.10931900
C	-2.98831200	0.61190900	-3.03903800
H	-3.49478600	0.49900200	-5.13409900
H	0.61632800	-0.34057700	-4.19136900
H	-3.99046400	0.86467200	-2.69021300
H	-1.19704900	-0.11417800	-5.84337400
C	-2.16448000	0.64442000	-0.62685200
O	-3.24124200	0.89926100	-0.13198500
O	-1.04677600	0.47175600	0.00653100
P	0.52482700	-0.00444400	-1.11732900
C	1.28120500	1.45068200	-0.28796000
C	2.53677300	1.31345300	0.31311000
C	0.63585700	2.69086100	-0.30533600
C	3.14186500	2.40887000	0.92377400
H	3.06208300	0.35633200	0.30265800
C	1.24829100	3.80081000	0.26674900
H	-0.34422800	2.80092400	-0.76964800
C	2.48477600	3.63128400	0.87918700
C	2.07048900	-0.38504600	-2.14686800
C	2.68992100	-1.63753700	-2.20708200
C	2.61521500	0.67373600	-2.89511100
C	3.81946400	-1.84107000	-3.00235200
H	2.31037700	-2.48100900	-1.63110000
C	3.73680200	0.49275200	-3.69318200
H	2.15634800	1.66544800	-2.85986800
C	4.31709700	-0.77185200	-3.73020600
C	0.41784200	-1.56831200	-0.18176600
C	-0.12395900	-2.68916000	-0.81919700
C	0.83907500	-1.63824800	1.14939800
C	-0.23933000	-3.89202600	-0.12844100
H	-0.45991400	-2.63636300	-1.85703500
C	0.73776600	-2.83954800	1.84424300

H	1.23288800	-0.75804300	1.65959700
C	0.20067300	-3.94106000	1.18850400
H	-0.65984800	-4.78017600	-0.59652200
H	4.11065200	2.32613800	1.41296100
H	4.16368600	1.30553200	-4.27797500
H	0.77709900	4.78178200	0.25163700
H	4.30969800	-2.81153000	-3.05746000
H	1.05903600	-2.92688900	2.88064400
N	3.12994400	4.80481900	1.50285900
O	2.54170800	5.85772500	1.45066000
O	4.20553400	4.63984100	2.02537500
N	5.50990400	-0.97994100	-4.57416000
O	5.96210700	-2.09767300	-4.64025100
O	5.96147500	-0.01909600	-5.15003200
N	0.08578100	-5.21547700	1.92500800
O	0.49297700	-5.24361000	3.06128900
O	-0.40928300	-6.15258200	1.34660500

Para-TS2(b): (ν = 486.67i(cm^{-1}))

0 1

C	0.71568500	-1.07556800	4.69774300
C	-0.61333100	-1.12256800	4.24103500
C	-1.08490300	-0.40676300	3.14217200
C	-0.13773700	0.36130700	2.39674000
C	1.15878100	0.57702900	3.01820100
C	1.58288500	-0.13249500	4.12917800
C	1.19609800	1.99749200	2.61828500
O	1.99214200	2.89758800	2.64351700
O	-0.08096000	2.07849800	2.15951300
P	-0.20532900	0.08891100	0.53449500
C	1.02214500	1.09912600	-0.32761000
C	2.36712600	0.75483600	-0.13655500
C	0.66983400	2.23945000	-1.05896600
C	3.36888300	1.56133900	-0.66890900
C	1.66480100	3.04026500	-1.60625600
C	2.99239900	2.68516100	-1.39125100
C	-1.88901200	0.43100700	-0.03542300
C	-2.65211000	-0.54905000	-0.68195800
C	-2.44368200	1.68495500	0.27200400
C	-3.96628000	-0.27259900	-1.04951500
C	-3.75402200	1.96651600	-0.09852200
C	-4.48319200	0.98088300	-0.75325000
C	0.25617700	-1.62314600	0.15276500
C	0.62164000	-2.50824700	1.17387900
C	0.34173000	-2.00866100	-1.19415100
C	1.05333200	-3.79316900	0.84754500
C	0.76394800	-3.29037200	-1.52487200
C	1.10778700	-4.15422300	-0.49063600
H	1.02110600	-1.67114800	5.55681300
H	-2.12435000	-0.49999200	2.82185900
H	2.53292900	0.11767200	4.60810300
H	-1.32171700	-1.77112500	4.76277100
H	2.64290600	-0.13752000	0.42889300
H	-0.37609200	2.50811600	-1.20993700
H	-2.23863900	-1.53443200	-0.89728800
H	1.42550500	3.92798800	-2.18872100
H	4.42299900	1.32775600	-0.53233700
H	-1.85795700	2.42654600	0.81874200
H	-4.58441300	-1.01331600	-1.55326700
H	-4.21397200	2.92859700	0.12025600
H	0.58459300	-2.21063900	2.22270200
H	0.09012200	-1.30862300	-1.99423700
H	1.34782500	-4.50449300	1.61696200
H	0.83644500	-3.62004200	-2.55957700
N	-5.87982600	1.27778100	-1.14240200
O	-6.31640000	2.36575100	-0.85739600
O	-6.49241000	0.41345200	-1.71978700
N	1.56513100	-5.51909000	-0.83588300
O	1.62078000	-5.80649400	-2.00628200
O	1.85237800	-6.25644900	0.07441500
N	4.05252700	3.53905700	-1.96812500
O	5.19827000	3.22138800	-1.76130000
O	3.70686600	4.50025900	-2.61082700

OMe:

Meta-$PPhe_3$:

0 1

P	-0.10587000	0.00945000	1.60710700
C	0.62809200	1.46258100	0.74510300
C	1.75334600	2.05937900	1.32216300
C	0.13072200	2.00853300	-0.44870800
C	2.38943300	3.15241000	0.73120500
H	2.15355100	1.66453700	2.26019900
C	0.74363500	3.10329200	-1.04293700
H	-0.75073900	1.57128000	-0.92229500
C	1.88139500	3.68255400	-0.46130900
H	3.26575600	3.58386400	1.21262700
H	0.35922300	3.53301200	-1.96890100
C	-1.71223100	-0.17486200	0.72442800
C	-1.88811200	-0.92175700	-0.45197400
C	-2.82229400	0.48993300	1.25296500
C	-3.12553000	-0.98675600	-1.07695200
H	-1.04296500	-1.45903300	-0.88877800
C	-4.07189800	0.44311000	0.63209800
H	-2.71789100	1.06543300	2.17683100
C	-4.22782000	-0.30084400	-0.54342900
H	-3.26989800	-1.56401500	-1.99122800
H	-4.90946400	0.97814600	1.07720500
C	0.86215300	-1.37949900	0.87957500
C	0.66554700	-2.65941100	1.42852700
C	1.77723500	-1.24755200	-0.16625200
C	1.34318100	-3.76383000	0.93703700

H	-0.03745500	-2.79159300	2.25565200
C	2.47929700	-2.34933600	-0.66504100
H	1.95623700	-0.26700000	-0.61312500
C	2.26141100	-3.61692600	-0.11557200
H	1.19091300	-4.75873500	1.35757300
H	3.18828400	-2.20353600	-1.47866100
O	-5.38901400	-0.41681600	-1.21912600
O	2.41376300	4.73890800	-1.10888700
O	2.88545500	-4.74057800	-0.52500000
C	3.55818500	5.35412800	-0.56318700
H	4.39963800	4.64640100	-0.49623000
H	3.82756500	6.17182300	-1.23981400
H	3.35351100	5.76509100	0.43825200
C	-6.52054500	0.26571700	-0.73001900
H	-6.34823800	1.35315700	-0.69801200
H	-7.34030200	0.05098000	-1.42344300
H	-6.79613600	-0.08502500	0.27714300
C	3.82656200	-4.64426000	-1.56900600
H	4.66037100	-3.97786000	-1.29635600
H	4.21343100	-5.65486100	-1.73682800
H	3.36042700	-4.27553900	-2.49660100

Meta-In1(a):

0 1

P	-0.18755800	-0.15112700	0.20838500
C	0.50619700	0.98833900	-1.01972200
C	1.18645400	2.12467500	-0.56463100
C	0.43340900	0.67847400	-2.37688800
C	1.80405100	2.96662600	-1.49411500
H	1.20235500	2.34577100	0.50195900
C	1.06001700	1.52694300	-3.29525800
H	-0.10453900	-0.20640500	-2.72149100
C	1.73848800	2.65713100	-2.86278500
H	1.01164500	1.30253600	-4.36153200
C	-1.67328000	-0.91025700	-0.51596700
C	-1.57011000	-2.10944900	-1.23919800
C	-2.89339800	-0.24991400	-0.39763800
C	-2.71362300	-2.63197300	-1.83442900
H	-0.61593000	-2.62926100	-1.33417300
C	-4.03897000	-0.79173700	-0.99796700
C	-3.94715400	-1.98742000	-1.72090800
H	-2.65106300	-3.56308600	-2.39934700
C	1.01861600	-1.49106600	0.43039100
C	0.64786600	-2.61546000	1.18727800
C	2.30846200	-1.37141900	-0.07586200
C	1.59022100	-3.60892400	1.41969300
H	-0.36378000	-2.71051300	1.58708800
C	3.25437300	-2.38153200	0.16565100
H	2.61795000	-0.50713900	-0.66597300
C	2.89067200	-3.50413500	0.91670100
H	1.31672700	-4.48822000	2.00456200
H	3.60596900	-4.30086900	1.11495500
H	2.22583100	3.33191000	-3.56750800
H	-4.82352500	-2.42430200	-2.19739700
H	-2.95246800	0.69114500	0.15713400
C	-0.08321800	1.03680100	4.09121600
C	-0.95777600	2.13046100	4.02934600
C	-1.57616300	2.48432300	2.82773200
C	-1.37512700	1.80226700	1.60076400
C	-0.49494000	0.71115600	1.75365400
C	0.15729000	0.30597500	2.93439300
H	0.40297700	0.76286600	5.02906400
H	-2.24779300	3.35280800	2.85898300
H	0.83688000	-0.54989700	2.95456100
H	-1.15118800	2.71047900	4.93662500
O	2.47121300	4.08757100	-1.16349500
O	4.47572800	-2.18347300	-0.36252400
O	-5.17881800	-0.09371500	-0.83185200
C	2.54935000	4.44458500	0.19947800
H	3.11549400	5.38035800	0.24456700
H	1.54740400	4.60251900	0.62879700
H	3.07515500	3.67285400	0.78349300
C	5.47322700	-3.15476500	-0.13471700
H	5.18498800	-4.12944900	-0.55900000
H	6.37824900	-2.79593500	-0.63525900
H	5.67485900	-3.27339200	0.94145000
C	-6.35739300	-0.57517300	-1.43695500
H	-7.15014400	0.13785900	-1.18866400
H	-6.24992700	-0.63123900	-2.53175700
H	-6.62949600	-1.56951200	-1.04893500

Meta-Product:

0 1

P	0.42547300	0.26054700	-1.27909000
C	-0.08136900	1.53861700	-0.04516700
C	-1.05377400	2.45654200	-0.47535900
C	0.42802000	1.61798800	1.25145400
C	-1.52869000	3.43808900	0.39970400
H	-1.43407700	2.39058300	-1.49612000
C	-0.04301100	2.61237400	2.11703000
H	1.18337400	0.90969200	1.59502100
C	-1.01321200	3.51308700	1.70353200
H	0.35291500	2.67872000	3.13195000
C	1.84625400	-0.53228700	-0.40805400
C	1.74494100	-1.69196100	0.37458900
C	3.08313700	0.09837700	-0.54497400
C	2.87943300	-2.18910300	1.01042100
H	0.78619100	-2.19927300	0.49160700
C	4.21966700	-0.39695400	0.10922700
C	4.11736300	-1.55336300	0.89151000
H	2.80650600	-3.09148300	1.62014800
C	-0.89931000	-1.00238700	-1.03749600
C	-0.97209800	-2.04587100	-1.97572500
C	-1.81066500	-0.96110800	0.01442800

C	-1.94072900	-3.03324700	-1.83450300
H	-0.27081700	-2.08078100	-2.81242300
C	-2.79611300	-1.95226000	0.14574700
H	-1.78417200	-0.16627900	0.76200600
C	-2.86002300	-2.99717100	-0.78200200
H	-1.99707600	-3.84716000	-2.55937700
H	-3.61447200	-3.77815900	-0.70119600
H	-1.39167000	4.29129300	2.36724200
H	4.98544200	-1.96583200	1.40334700
H	3.19649200	0.99666000	-1.15709100
O	5.36353900	0.29577900	-0.07506300
O	-2.46590800	4.35329900	0.07486600
O	-3.63449400	-1.81963300	1.19582300
C	6.53266700	-0.15857600	0.56626000
H	7.33200700	0.53758600	0.29163700
H	6.41396400	-0.15972800	1.66149600
H	6.80533600	-1.17255300	0.23290600
C	-4.64602500	-2.78440900	1.37093400
H	-4.21993300	-3.78901900	1.52269000
H	-5.20770700	-2.49261300	2.26443100
H	-5.32846200	-2.81035400	0.50643800
C	-3.02590300	4.31271000	-1.21723500
H	-3.76143100	5.12245400	-1.26582300
H	-2.25946400	4.47300500	-1.99211300
H	-3.53116600	3.35168100	-1.40412600

Meta-TS2(b): (ν = 486.56i(cm^{-1}))

0 1

C	0.68793300	-1.07302300	4.72621900
C	-0.64138600	-1.10149200	4.26761200
C	-1.10660000	-0.36769200	3.17921500
C	-0.15453400	0.39862200	2.43165900
C	1.14700700	0.59222000	3.06041800
C	1.56076100	-0.12790500	4.16685600
C	1.18841000	2.02121300	2.69524000
O	1.99109900	2.91751500	2.74664800
O	-0.08715000	2.12211900	2.24927000
P	-0.24163900	0.14047900	0.57860600
C	0.96266200	1.16489300	-0.29519300
C	2.30248000	0.81436600	-0.13672600
C	0.59781300	2.31709400	-1.00508800
C	3.30727900	1.63090400	-0.67710700
C	1.60210100	3.10865900	-1.54822000
C	2.95155600	2.78367900	-1.38573600
C	-1.93905100	0.48216400	0.05829100
C	-2.71970000	-0.51183800	-0.52760000
C	-2.48280100	1.74702700	0.34740100
C	-4.05780900	-0.24721200	-0.85925300
C	-3.80715400	1.99512800	0.00728200
C	-4.59975100	1.01502900	-0.59536800
C	0.21195100	-1.56825900	0.19787400
C	0.65624600	-2.42959700	1.20028000
C	0.20034000	-1.96202000	-1.15201300
C	1.07916400	-3.71527300	0.83874900
C	0.61115200	-3.25214800	-1.49284200
C	1.05223800	-4.12636000	-0.48332900
H	0.98818000	-1.67915700	5.57988000
H	-2.14747500	-0.44602300	2.85902300
H	2.50958300	0.11632500	4.65218500
H	-1.35614100	-1.74973600	4.78175400
H	2.60531600	-0.08377700	0.40579500
H	-0.44949600	2.59037400	-1.13240900
H	-2.33328500	-1.50989600	-0.73657600
H	1.33700900	4.00404300	-2.11199500
H	-1.88008600	2.50397400	0.85082600
H	-4.24358700	2.97179800	0.22058800
H	0.68717300	-2.12029100	2.24543500
H	-0.12007200	-1.25836500	-1.92142400
H	1.43424700	-4.40054000	1.60923600
H	-5.63534400	1.24265600	-0.84363000
H	3.71208300	3.43155000	-1.81857600
H	1.37496500	-5.12622900	-0.77585300
O	-4.73633300	-1.26500900	-1.41870800
O	4.57304900	1.22749100	-0.46711900
O	0.62247600	-3.73427100	-2.74780000
C	0.16139500	-2.90483200	-3.79203400
H	-0.88656400	-2.60726600	-3.62832100
H	0.78486200	-2.00220500	-3.88880500
H	0.23067200	-3.49313600	-4.71250100
C	-6.09116200	-1.06714900	-1.75974800
H	-6.20140800	-0.26862800	-2.51005200
H	-6.44713700	-2.01206600	-2.18259000
H	-6.69311000	-0.81727200	-0.87206800
C	5.62510100	2.01307400	-0.98301900
H	6.55608000	1.51281600	-0.69767400
H	5.57079400	2.08091500	-2.08096500
H	5.61086800	3.02782500	-0.55543900

Para-$PPhe_3$:

0 1

P	-0.10587000	0.00945000	1.60710700
C	0.62809200	1.46258100	0.74510300
C	1.75334600	2.05937900	1.32216300
C	0.13072200	2.00853300	-0.44870800
C	2.38943300	3.15241000	0.73120500
H	2.15355100	1.66453700	2.26019900
C	0.74363500	3.10329200	-1.04293700
H	-0.75073900	1.57128000	-0.92229500

C	1.88139500	3.68255400	-0.46130900
H	3.26575600	3.58386400	1.21262700
H	0.35922300	3.53301200	-1.96890100
C	-1.71223100	-0.17486200	0.72442800
C	-1.88811200	-0.92175700	-0.45197400
C	-2.82229400	0.48993300	1.25296500
C	-3.12553000	-0.98675600	-1.07695200
H	-1.04296500	-1.45903300	-0.88877800
C	-4.07189800	0.44311000	0.63209800
H	-2.71789100	1.06543300	2.17683100
C	-4.22782000	-0.30084400	-0.54342900
H	-3.26989800	-1.56401500	-1.99122800
H	-4.90946400	0.97814600	1.07720500
C	0.86215300	-1.37949900	0.87957500
C	0.66554700	-2.65941100	1.42852700
C	1.77723500	-1.24755200	-0.16625200
C	1.34318100	-3.76383000	0.93703700
H	-0.03745500	-2.79159300	2.25565200
C	2.47929700	-2.34933600	-0.66504100
H	1.95623700	-0.26700000	-0.61312500
C	2.26141100	-3.61692600	-0.11557200
H	1.19091300	-4.75873500	1.35757300
H	3.18828400	-2.20353600	-1.47866100
O	-5.38901400	-0.41681600	-1.21912600
O	2.41376300	4.73890800	-1.10888700
O	2.88545500	-4.74057800	-0.52500000
C	3.55818500	5.35412800	-0.56318700
H	4.39963800	4.64640100	-0.49623000
H	3.82756500	6.17182300	-1.23981400
H	3.35351100	5.76509100	0.43825200
C	-6.52054500	0.26571700	-0.73001900
H	-6.34823800	1.35315700	-0.69801200
H	-7.34030200	0.05098000	-1.42344300
H	-6.79613600	-0.08502500	0.27714300
C	3.82656200	-4.64426000	-1.56900600
H	4.66037100	-3.97786000	-1.29635600
H	4.21343100	-5.65486100	-1.73682800
H	3.36042700	-4.27553900	-2.49660100

Para-In1(a):

0 1

P	0.97936600	-0.13443600	-0.12681100
C	1.10346100	-0.16969900	1.67339300
C	2.32065300	0.10970300	2.29744100
C	-0.03654000	-0.40267700	2.45856500
C	2.41314300	0.16265700	3.68712900
H	3.21448100	0.27515200	1.69310800
C	0.04526400	-0.35225400	3.84026700
H	-0.99548600	-0.62990800	1.98664000
C	1.27106700	-0.06764500	4.46646900
H	3.37594100	0.37606300	4.14812500
C	-0.23207700	-1.38123900	-0.64012400
C	-1.59023900	-1.04564700	-0.77784000
C	0.16850800	-2.70501400	-0.84712400
C	-2.51886700	-2.01305000	-1.12677700
H	-1.92837700	-0.02003600	-0.61742400
C	-0.75990300	-3.68149800	-1.20148900
H	1.22673200	-2.95391800	-0.72248700
C	-2.11183700	-3.33966800	-1.34404500
H	-0.41840700	-4.70306600	-1.36089300
C	0.28126200	1.47142000	-0.58404000
C	-0.12756500	1.68787700	-1.90527200
C	0.20522200	2.52887700	0.33432400
C	-0.61093700	2.92785700	-2.31306200
H	-0.07244900	0.87802800	-2.63751700
C	-0.27447000	3.76833000	-0.06153500
H	0.52018500	2.38232500	1.36943000
C	-0.68692900	3.97910000	-1.38669200
H	-0.34362000	4.59842300	0.64166000
H	-3.57509900	-1.76835700	-1.24101800
H	-0.92366200	3.06357000	-3.34674700
H	-0.82751900	-0.53544400	4.46736600
C	4.41328500	0.41868000	-2.22576300
C	5.08187300	-0.77596200	-1.92774800
C	4.49085900	-1.73738000	-1.10403800
C	3.21126300	-1.59752700	-0.50819700
C	2.60236700	-0.37657300	-0.86894900
C	3.14842700	0.62912700	-1.68976400
H	4.87525300	1.17314600	-2.86497200
H	5.07695100	-2.64698600	-0.91396600
H	2.60930200	1.55574200	-1.90309900
H	6.07846700	-0.94764100	-2.34562900
C	-1.57649200	5.47331600	-2.99770400
H	-1.89651700	6.52002300	-3.01302800
H	-0.76165600	5.33175100	-3.72436400
H	-2.42591300	4.82922200	-3.27323700
C	-2.72534000	-5.55908400	-1.90324700
H	-2.29069300	-6.01115500	-0.99823400
H	-3.65099900	-6.08390400	-2.16023400
H	-2.01075700	-5.65293800	-2.73552700
C	2.46044600	0.23587600	6.49443900
H	2.84873900	1.23184800	6.23067600
H	2.22200500	0.21208200	7.56252300
H	3.22718700	-0.52330400	6.27563000
O	-1.14012800	5.20713900	-1.68159400
O	1.25784800	-0.04040100	5.80847400
O	-3.07714200	-4.21025700	-1.68125800

Para-Product:

0 1

C	-2.69945600	0.46774800	-4.39759400
C	-1.39957200	0.14214400	-4.78837300
C	-0.38810700	-0.00446400	-3.83791000
C	-0.68311600	0.18429900	-2.48195100
C	-1.99190200	0.49288500	-2.09556500
C	-2.99735800	0.63708600	-3.04655500
H	-3.48116100	0.58143500	-5.15024100
H	0.61688100	-0.27055800	-4.16319900
H	-4.00396100	0.87637800	-2.70051600
H	-1.16679800	-0.00351600	-5.84393100
C	-2.26522800	0.62903800	-0.61035200
O	-3.39829900	0.85712700	-0.20745000
O	-1.20512100	0.48054800	0.07798900
P	0.56330500	-0.03911100	-1.15628800
C	1.22497400	1.41757500	-0.29405900
C	2.48105500	1.32535500	0.32418000
C	0.55453300	2.64038900	-0.29251600
C	3.03366200	2.42660800	0.95938400
H	3.03901300	0.38612900	0.30768100
C	1.11795600	3.76332200	0.31041000
H	-0.42165900	2.73051900	-0.76969100
C	2.35849800	3.65751500	0.95426400
C	2.07493500	-0.42826900	-2.16302200
C	2.64615000	-1.69751200	-2.25526500
C	2.68059900	0.61979300	-2.88489100
C	3.77607400	-1.93643400	-3.04502600
H	2.22317800	-2.53636100	-1.70156700
C	3.79311000	0.39981200	-3.67676500
H	2.27099800	1.63201000	-2.82500900
C	4.35280400	-0.88757500	-3.76554200
C	0.35639700	-1.56666800	-0.20748200
C	-0.18146000	-2.68848500	-0.83581600
C	0.72708400	-1.64217500	1.14146400
C	-0.34195100	-3.88821100	-0.14022300
H	-0.48645600	-2.64476000	-1.88465200
C	0.57834800	-2.83120200	1.83733800
H	1.12014300	-0.76352900	1.65662000
C	0.04474600	-3.96539700	1.20304700
H	-0.76943200	-4.74690100	-0.65500600
H	4.00016100	2.36445700	1.45990700
H	4.26166200	1.20954800	-4.23710600
H	0.57537100	4.70644000	0.28186700
H	4.19211300	-2.94182200	-3.08387900
H	0.86071300	-2.90948700	2.88753700
O	2.97094500	4.67539300	1.58196900
O	5.43480700	-1.01358700	-4.55416600
O	-0.06470100	-5.07390900	1.95755700
C	6.04419900	-2.28092400	-4.66410000
H	6.40944900	-2.63419700	-3.68707700
H	5.34586800	-3.02410500	-5.07981400
H	6.89211300	-2.16162100	-5.34613900
C	-0.58446100	-6.24327800	1.36463900
H	-1.61862700	-6.09005000	1.01844800
H	0.03719200	-6.56941700	0.51597600
H	-0.57376000	-7.01699200	2.13905100
C	2.33581700	5.93527300	1.60858400
H	2.19789400	6.33481100	0.59181300
H	1.35820100	5.87719000	2.11200000
H	2.99353400	6.60353000	2.17362600

Para-TS2(b): (ν = 488.89i(cm^{-1}))

0 1

C	0.97253600	-1.79007300	4.01654700
C	-0.39298200	-1.68232100	3.69423600
C	-0.90758100	-0.77231300	2.77417900
C	0.01827000	0.05089900	2.05179600
C	1.38319200	0.08084800	2.56879600
C	1.84810100	-0.81545900	3.51432300
C	1.47862300	1.54594900	2.42308100
O	2.33859500	2.38364400	2.52503200
O	0.17476900	1.77618600	2.13632000
P	-0.23256100	0.08514700	0.20116900
C	0.96765400	1.15935100	-0.60139000
C	2.28790300	0.71055300	-0.70218900
C	0.65114000	2.46466600	-1.01071700
C	3.29006800	1.54244600	-1.19962600
C	1.63682400	3.29254700	-1.51556500
C	2.96662900	2.84213600	-1.60839900
C	-1.92785600	0.59970100	-0.12227600
C	-2.81207600	-0.19964300	-0.85173100
C	-2.39478100	1.80615700	0.43900500
C	-4.13344000	0.19526100	-1.05425200
C	-3.70384800	2.20463500	0.23738700
C	-4.58470000	1.40730600	-0.51586800
C	0.07406700	-1.56409700	-0.45352900
C	0.50412300	-2.61215300	0.36533900
C	-0.00875800	-1.76881800	-1.84448100
C	0.82182300	-3.85575900	-0.18194400
C	0.29565900	-3.00090000	-2.39210100
C	0.71104400	-4.05925600	-1.56265000
H	1.31185800	-2.53020200	4.73996600
H	-1.97622600	-0.75066200	2.55160000
H	2.85032900	-0.69141700	3.93403900
H	-1.09785100	-2.36402600	4.17824500
H	2.54941300	-0.30414300	-0.39297100

H	-0.37231100	2.83576800	-0.93814000
H	-2.48335200	-1.15330600	-1.26719800
H	1.40929700	4.30586300	-1.84704100
H	4.30907500	1.16583800	-1.26517500
H	-1.72632400	2.41670500	1.04973800
H	-4.79605700	-0.45040100	-1.62775200
H	-4.08218400	3.13505000	0.66151800
H	0.60799100	-2.47515600	1.44297300
H	-0.30651100	-0.95164200	-2.50631200
H	1.15677000	-4.65241600	0.47966300
H	0.23210600	-3.17639700	-3.46604200
O	3.85277300	3.71770700	-2.10504200
O	-5.83184800	1.87596300	-0.65912800
O	0.98756000	-5.21739300	-2.17748800
C	5.20236400	3.32195000	-2.22952800
H	5.30196700	2.46088200	-2.90842100
H	5.73986600	4.17803600	-2.64956200
H	5.63352600	3.06589100	-1.24949600
C	1.43455200	-6.31059400	-1.40260300
H	0.67815300	-6.60555000	-0.65913400
H	1.59928700	-7.13759400	-2.10027400
H	2.37818700	-6.07323500	-0.88774000
C	-6.76737500	1.10983400	-1.38896500
H	-7.70457500	1.67514900	-1.38141600
H	-6.43698300	0.96597900	-2.42917600
H	-6.92888300	0.12780500	-0.91822100

Cy:

$PPhe_3$:

0 1

P	0.00421500	-0.00994200	-1.30987500
C	1.50001600	0.86929000	-0.59456000
C	1.40352500	2.35818800	-0.97127400
C	1.90378600	0.69219600	0.87654000
H	2.32694700	0.44934500	-1.19872700
C	2.68884400	3.11521900	-0.63797400
H	0.56684500	2.82405900	-0.42190300
H	1.16768800	2.45687300	-2.04332400
C	3.18525600	1.46966600	1.19537900
H	1.09540900	1.03238900	1.54265600
H	2.06491800	-0.37238800	1.09814800
C	3.05604500	2.94832100	0.83507400
H	2.57447500	4.18058000	-0.89017800
H	3.51108900	2.72574400	-1.26305500
H	3.43802200	1.35152800	2.26027300
H	4.02089800	1.03221200	0.62113800
H	3.99063800	3.48319900	1.06298800
H	2.26729900	3.40577300	1.45834900
C	-1.50105100	0.85589400	-0.60095100
C	-2.75351000	0.04177800	-0.96895000
C	-1.54059900	1.29737400	0.86822100
H	-1.54173900	1.78012800	-1.20852600
C	-4.03931200	0.80126400	-0.63968800
H	-2.75027600	-0.90914500	-0.40812700
H	-2.72670700	-0.22291800	-2.03843000
C	-2.83881500	2.04701600	1.18406900
H	-1.45380200	0.42468900	1.53436200
H	-0.68480800	1.94947500	1.09075400
C	-4.07051200	1.21561000	0.83032700
H	-4.91610200	0.18371200	-0.88775000
H	-4.09577900	1.70441400	-1.27189700
H	-2.85711700	2.33327800	2.24692000
H	-2.85997000	2.98491000	0.60165400
H	-4.99112300	1.77575800	1.05416700
H	-4.08782700	0.30906600	1.46085500
C	0.00812600	-1.74412700	-0.59854700
C	1.33959200	-2.41697700	-0.97473600
C	-0.34548500	-2.00154900	0.87300300
H	-0.77406600	-2.24314800	-1.20221700
C	1.33515400	-3.90985000	-0.64566700
H	2.16343600	-1.93367400	-0.42064300
H	1.54738200	-2.26007000	-2.04555400
C	-0.33324500	-3.50063200	1.18931200
H	0.36596100	-1.48281300	1.53457500
H	-1.34076300	-1.59456100	1.10080300
C	1.00269300	-4.14563100	0.82646300
H	2.30988700	-4.35443100	-0.89846400
H	0.58128300	-4.41586200	-1.27349400
H	-0.56271000	-3.66024300	2.25401400
H	-1.13720800	-3.99367300	0.61481500
H	0.98359300	-5.22320000	1.04976400
H	1.79938700	-3.70459100	1.45136800

TS2(b): (ν = 484.77i(cm^{-1}))

0 1

C	1.51911200	-3.55976400	1.80327700
C	2.20802600	-2.36116800	2.06498400
C	1.64411400	-1.09314200	1.96574500
C	0.28699400	-0.97488000	1.49501400
C	-0.46401500	-2.23966100	1.49036600
C	0.13028100	-3.48165900	1.62522300
H	2.02640800	-4.51993000	1.88498400
H	2.24051300	-0.21506500	2.21335500
H	-0.49808000	-4.37348100	1.70596200
H	3.26022500	-2.41916800	2.35805000
C	-1.62442500	-1.61541500	2.14599100
O	-2.76862200	-1.91766300	2.38483200
O	-1.03883200	-0.43595100	2.46069200
P	-0.00509200	0.17195200	0.03716100
C	0.93060100	1.74013600	0.22498300
C	0.60197600	2.36914500	1.59172700

C	2.44642200	1.67636100	-0.02527100
H	0.50619300	2.38487900	-0.56457300
C	1.23913100	3.75278400	1.71602200
H	0.98009800	1.71020200	2.39122200
H	-0.48779500	2.43169300	1.73756000
C	3.05448900	3.07394300	0.10882200
H	2.92689400	0.99580200	0.69247200
H	2.65290800	1.27892700	-1.02852400
C	2.74598100	3.69376700	1.47050500
H	1.02371300	4.16899700	2.71084400
H	0.77494100	4.42988800	0.97835500
H	4.14061900	3.01642200	-0.05365300
H	2.64649900	3.72126200	-0.68643600
H	3.18385500	4.70042300	1.54020500
H	3.21798700	3.08520400	2.26119000
C	-1.79886400	0.61750500	-0.14199200
C	-2.08786300	1.97609800	-0.80325000
C	-2.61426500	-0.50240900	-0.81447700
H	-2.12552000	0.69185100	0.90518000
C	-3.58840200	2.27515800	-0.72894000
H	-1.76916400	1.97378000	-1.85848500
H	-1.53867500	2.78415700	-0.29885500
C	-4.10803300	-0.18938100	-0.72830800
H	-2.32442400	-0.57974200	-1.87696700
H	-2.40708600	-1.47646500	-0.34746400
C	-4.42398400	1.16398400	-1.36094300
H	-3.79249100	3.23932600	-1.21748000
H	-3.87360900	2.38688800	0.33090500
H	-4.67938900	-0.98975300	-1.22099800
H	-4.40659900	-0.18871400	0.33368400
H	-5.49556300	1.39251200	-1.26354000
H	-4.20547600	1.11640700	-2.44190800
C	0.49317700	-0.75650200	-1.46894800
C	0.48686800	0.14766500	-2.71387600
C	1.79405300	-1.56531800	-1.33235900
H	-0.32312700	-1.49346800	-1.57229500
C	0.80461500	-0.65304200	-3.97781300
H	1.23674900	0.94777300	-2.59603900
H	-0.48920300	0.64180700	-2.82594600
C	2.08852500	-2.32958100	-2.62494500
H	2.63993000	-0.89907800	-1.09898100
H	1.70736800	-2.27450600	-0.49848800
C	2.12504400	-1.40588000	-3.83936800
H	0.82820900	0.02537600	-4.84308900
H	-0.00963300	-1.37544800	-4.15904200
H	3.04107300	-2.86865400	-2.51634000
H	1.30667700	-3.09411300	-2.77491400
H	2.33617600	-1.98002800	-4.75354100
H	2.94745600	-0.67880100	-3.72100900

In1(a)

0 1

P	-0.23609400	-1.09951900	-2.08976900
C	1.24937900	-0.07642200	-1.69579300
C	1.23510100	1.28869900	-2.40182300
C	1.54901100	0.07545000	-0.19452400
H	2.06310500	-0.67564400	-2.14476000
C	2.54222500	2.04544600	-2.15413100
H	0.38802900	1.89140900	-2.02839700
H	1.08902100	1.15487600	-3.48213500
C	2.86411200	0.82683000	0.01894400
H	0.73700600	0.62900100	0.30016900
H	1.60335700	-0.90600000	0.29344400
C	2.83958200	2.19189900	-0.66395800
H	2.49110000	3.03113900	-2.63959200
H	3.36772200	1.49529400	-2.63707400
H	3.05390100	0.93446800	1.09697000
H	3.69265800	0.22553300	-0.39328800
H	3.79692100	2.71285800	-0.51495400
H	2.05928100	2.81709900	-0.19609600
C	-1.79499700	-0.17715700	-1.74806100
C	-3.00042600	-1.09229800	-2.03203700
C	-1.91061700	0.54143800	-0.39656100
H	-1.78666600	0.60302500	-2.53113300
C	-4.31514900	-0.32321800	-1.90658900
H	-3.00721800	-1.93080200	-1.31574200
H	-2.90489400	-1.53270000	-3.03565400
C	-3.24947000	1.27732800	-0.29094600
H	-1.81966000	-0.17908300	0.43165100
H	-1.09304500	1.26646600	-0.28425300
C	-4.43351500	0.34628300	-0.53928300
H	-5.15855000	-1.00730700	-2.08084000
H	-4.35993900	0.44746500	-2.69515100
H	-3.32797800	1.75176800	0.69830900
H	-3.26831200	2.09121100	-1.03618300
H	-5.37874000	0.90410100	-0.46509500
H	-4.45861800	-0.43048600	0.24490400
C	-0.13134200	-2.68589300	-1.17564800
C	1.29502800	-3.25779400	-1.26729000
C	-0.66947300	-2.72638400	0.26172200
H	-0.75493300	-3.33079300	-1.82124300
C	1.32421600	-4.68575800	-0.72599900
H	1.99610300	-2.63908000	-0.68380600
H	1.61052900	-3.24604700	-2.31929000
C	-0.60841500	-4.16063200	0.79787700
H	-0.07752500	-2.06767400	0.91835500
H	-1.70720500	-2.36764100	0.30390900
C	0.80036800	-4.74448200	0.70733900
H	2.34892600	-5.08253400	-0.78076900
H	0.69858000	-5.32196700	-1.37569100
H	-0.97056600	-4.18017100	1.83652900
H	-1.29705000	-4.78808500	0.20660700

H	0.80647800	-5.77987500	1.07953100
H	1.47482400	-4.16594200	1.36278200
C	-0.66058600	-0.74445600	-6.12670800
C	-0.46726000	-2.06401500	-6.55509500
C	-0.20144700	-3.06953500	-5.62597400
C	-0.09978200	-2.86505500	-4.22506600
C	-0.28584300	-1.51420100	-3.86799900
C	-0.57352500	-0.46372800	-4.76905600
H	-0.88194500	0.05051700	-6.84109600
H	-0.07365700	-4.08392800	-6.02967100
H	-0.74483400	0.56376800	-4.43424200
H	-0.53549500	-2.29857800	-7.62159900

Product:

0 1

C	-3.27587700	0.97959300	-3.62821500
C	-2.58384600	-0.18457200	-3.95840500
C	-1.41656200	-0.50318400	-3.27277300
C	-0.93651900	0.30898300	-2.22998500
C	-1.68006600	1.44270700	-1.84336600
C	-2.81732400	1.78013800	-2.58704100
H	-4.17883300	1.25565300	-4.17534000
H	-0.88331100	-1.40765400	-3.55724900
H	-3.35256000	2.68871200	-2.30671200
H	-2.93904900	-0.83928600	-4.75453400
C	-1.41179100	2.30011900	-0.59239800
O	-1.41492100	3.52826900	-0.75617200
O	-1.25982100	1.64613000	0.46089500
P	0.63933100	-0.22010300	-1.46884700
C	0.32431700	-1.66437800	-0.37739100
C	-0.37293400	-1.26553300	0.93080000
C	-0.45258600	-2.77706900	-1.09826000
H	1.32787100	-2.04944800	-0.12976100
C	-0.54554300	-2.49029500	1.83033500
H	-1.34483100	-0.80398900	0.69837400
H	0.20410200	-0.48838900	1.45358900
C	-0.60221900	-3.99645100	-0.18746400
H	-1.45545400	-2.40194300	-1.36035400
H	0.04362100	-3.06115400	-2.04045000
C	-1.29428200	-3.61695900	1.12026200
H	-1.07433000	-2.19653300	2.74886100
H	0.44942100	-2.85502600	2.13918900
H	-1.16725500	-4.77986000	-0.71337000
H	0.39660700	-4.41271600	0.02921100
H	-1.37570500	-4.49513000	1.77773800
H	-2.32346800	-3.28576900	0.89965900
C	1.91627200	-0.68156600	-2.75240500
C	2.82757800	-1.82357200	-2.26478900
C	1.49130600	-0.92857800	-4.20651000
H	2.52639200	0.23791700	-2.76888600
C	4.05613500	-1.94803100	-3.16874000
H	2.26284900	-2.77086900	-2.27942800
H	3.15585000	-1.66262400	-1.22907700
C	2.73143700	-1.02690000	-5.09848300
H	0.93447600	-1.87701900	-4.28140000
H	0.83136700	-0.12611200	-4.56314200
C	3.66418200	-2.14153100	-4.63131300
H	4.68118400	-2.78319700	-2.82056100
H	4.66410100	-1.03213400	-3.07057400
H	2.41780800	-1.19483700	-6.13907000
H	3.26831600	-0.06318200	-5.07832200
H	4.56244200	-2.18027000	-5.26496100
H	3.15255000	-3.11275000	-4.74436700
C	1.46178400	1.10735500	-0.51168900
C	2.78492100	0.65802000	0.12606000
C	1.66675500	2.39233600	-1.33205600
H	0.74412000	1.32901900	0.29040000
C	3.29509600	1.76675400	1.05180900
H	3.54136500	0.46619100	-0.65457500
H	2.65704900	-0.27414800	0.69895700
C	2.17372200	3.49985800	-0.40657500
H	2.41014700	2.21836900	-2.12928300
H	0.73292100	2.71630400	-1.81028700
C	3.46336600	3.08820000	0.30263100
H	4.24709900	1.45722300	1.50764900
H	2.57243500	1.90015200	1.87417600
H	2.33137000	4.42178300	-0.98546600
H	1.38360600	3.71710500	0.33159400
H	3.78798500	3.87767500	0.99654400
H	4.26565600	2.97543700	-0.44726700

Furyl:

PPhe$_3$:

0 1

P	-0.02720400	-0.02354000	-1.19974300
C	-1.60515200	0.40752200	-0.41346000
C	-2.78571900	0.79044800	-0.98775500
C	-3.71041300	0.99540800	0.08509500
H	-2.96542300	0.90769000	-2.05335900
C	-3.01817500	0.71484700	1.22500800
H	-4.74950100	1.30432800	0.01815400
H	-3.28820100	0.72092800	2.27733000
C	0.45382600	-1.49654100	-0.23426300
C	1.15357900	-1.67623400	0.92660500
C	1.23821000	-3.09121400	1.12717400
H	1.55468800	-0.88614900	1.55684300

C	0.58386300	-3.65611900	0.07361400
H	1.72218500	-3.61741500	1.94515500
H	0.38078600	-4.68481700	-0.20975200
C	1.05054400	1.18013300	-0.35471200
C	0.84057800	2.15414500	0.58106800
C	2.10812400	2.79206500	0.78324100
H	-0.10241000	2.37565900	1.07304000
C	2.98298200	2.15366000	-0.04226500
H	2.33627000	3.61367300	1.45621200
H	4.04465700	2.27286800	-0.23732800
O	2.35212100	1.18583300	-0.73712800
O	0.11534600	-2.70351300	-0.75500600
O	-1.75227700	0.35988100	0.93635200

TS2(b): (ν = 480.23i(cm^{-1}))

0 1

C	-3.44571500	1.95884100	-0.79504400
C	-2.30407400	2.30275500	-1.54357400
C	-1.19963300	1.47227700	-1.71239400
C	-1.17927400	0.24174900	-0.98312300
C	-2.44566800	-0.18140800	-0.41052200
C	-3.54624400	0.65522100	-0.30209800
H	-4.27260600	2.66157900	-0.70266600
H	-0.34017100	1.80667300	-2.29684500
H	-4.49501500	0.26352300	0.07370200
H	-2.27139400	3.28465300	-2.02326700
C	-2.24286600	-1.57946200	-0.80394600
O	-2.79927500	-2.63544400	-0.64361400
O	-1.10596500	-1.39269500	-1.53449800
P	0.41415100	-0.04070700	-0.02977300
C	0.77023600	1.42221400	0.92335200
C	1.76033000	2.36399500	0.87724800
C	1.39468900	3.35238600	1.84196600
H	2.63775500	2.34463400	0.23703700
C	0.21640000	2.93139100	2.38525600
H	1.93963500	4.25628100	2.09687200
H	-0.44009900	3.34267400	3.14639600
C	0.32202100	-1.38615900	1.14492300
C	-0.00457200	-2.71010900	1.01729300
C	0.14189900	-3.27059200	2.32031500
H	-0.32688000	-3.20436800	0.10464700
C	0.54158300	-2.24555900	3.12897200
H	-0.03382600	-4.30013100	2.61750800
H	0.77107700	-2.18257200	4.18877200
C	1.74633700	-0.23557000	-1.19577200
C	1.71134100	-0.68397500	-2.48760200
C	3.06779500	-0.67577400	-2.93373800
H	0.81673300	-0.98868900	-3.02550300
C	3.80851500	-0.23061600	-1.87799500
H	3.44195800	-0.96495500	-3.91115900
H	4.87131800	-0.05589400	-1.73810500
O	-0.16973200	1.76793600	1.83463300
O	0.65599800	-1.10666800	2.42924900
O	3.02030500	0.03406700	-0.82101000

In1(a)

0 1

P	0.80508000	-0.04447300	-1.99531000
C	-0.90075600	0.27545300	-1.58467600
C	-1.99008800	0.46589200	-2.38583800
C	-3.09212500	0.65541000	-1.49632800
H	-1.98635700	0.44821200	-3.47260900
C	-2.57954800	0.55966300	-0.23582100
H	-4.12995200	0.83620600	-1.75967600
H	-3.02165700	0.63509700	0.75340600
C	1.23643800	-1.62633400	-1.27092100
C	2.40058400	-2.06555900	-0.70352100
C	2.16032900	-3.42549600	-0.33598800
H	3.31030900	-1.48601600	-0.56923000
C	0.87421900	-3.69093800	-0.70523200
H	2.85239100	-4.11214200	0.14230100
H	0.24610900	-4.57361600	-0.62938200
C	1.74707500	1.17376000	-1.07664600
C	1.37544400	2.24503600	-0.31506100
C	2.59476100	2.86884800	0.09638100
H	0.35793000	2.54317500	-0.07620300
C	3.60020500	2.12754400	-0.44895000
H	2.70523200	3.75259200	0.71763800
H	4.68288500	2.20501600	-0.41791500
O	3.09770200	1.10210500	-1.16305100
O	0.31065400	-2.60902300	-1.26982000
O	-1.25498600	0.32700200	-0.27798500
C	1.27377500	0.08557600	-6.44902200
C	1.88355800	1.11481900	-5.71361300
C	1.76885000	1.10438600	-4.33086100
C	1.04783500	0.03982400	-3.75624400
C	0.40961200	-1.03237600	-4.40316300
C	0.56380400	-0.93429600	-5.81209200
H	1.36165900	0.09074500	-7.53945400
H	2.22455400	1.89233100	-3.72407100
H	0.11389400	-1.69689900	-6.46185000
H	2.43539400	1.90893600	-6.21929600

Product:

0 1

C	-2.64599700	0.57601300	-4.46769500
C	-1.39059400	0.07470700	-4.82333100
C	-0.40517600	-0.14605600	-3.85904200
C	-0.69674800	0.14156200	-2.52395500
C	-1.95853400	0.61770700	-2.17491200
C	-2.94061700	0.84607500	-3.13186800

H	-3.39978800	0.74275400	-5.23840000
H	0.56538100	-0.53826100	-4.15914400
H	-3.91739700	1.21625300	-2.81725100
H	-1.17401700	-0.14921700	-5.86873000
C	-2.16581300	0.78043600	-0.69767300
O	-3.19494200	1.18313400	-0.20442100
O	-1.10461100	0.40324000	-0.04236700
P	0.41896600	-0.07172000	-1.08130300
C	1.22513500	1.36005700	-0.31348000
C	2.56143100	1.64960600	-0.18960700
C	2.63849800	2.90341200	0.48345500
H	3.38990500	1.03917500	-0.53965100
C	1.34518900	3.28360500	0.69983700
H	3.53293200	3.45191100	0.76334700
H	0.89690900	4.15880000	1.16171100
C	0.33945300	-1.64739900	-0.19282200
C	0.34192100	-2.92786200	-0.67176600
C	0.15218100	-3.77168700	0.46801300
H	0.45981600	-3.22780600	-1.71011600
C	0.05798000	-2.93270100	1.53816000
H	0.09530100	-4.85593300	0.48604500
H	-0.07234000	-3.09791000	2.60356000
C	1.96690200	-0.51221300	-2.03580500
C	2.92166200	-1.48974000	-1.95265600
C	3.95759300	-1.13242100	-2.87650400
H	2.90220300	-2.35422000	-1.29624800
C	3.55687900	0.03604400	-3.44935300
H	4.87533000	-1.67501100	-3.08410200
H	3.99389000	0.68571400	-4.20216400
O	0.49514600	2.36170700	0.23124700
O	2.36537400	0.41487100	-2.94942200
O	0.16016200	-1.65049000	1.14425600

Thiophene:

$PPhe_3$:

0 1

P	0.09998200	0.02391300	-1.22362800
C	-0.94405400	1.34794900	-0.53471700
C	-1.22584300	2.54061100	-1.15717600
S	-1.68942300	1.27675400	1.02632500
C	-2.03678500	3.40531900	-0.36437400
H	-0.86110700	2.78659300	-2.15518800
C	-2.36382600	2.84503400	0.84178900
H	-2.37316100	4.39171500	-0.68141300
H	-2.98592700	3.26905800	1.62802700
C	-0.52952500	-1.40519400	-0.26428500
C	-0.05696600	-1.95352800	0.90394700
S	-1.92920400	-2.25661300	-0.82024500
C	-0.83933700	-3.05855800	1.35399300
H	0.82343200	-1.57051500	1.42155100
C	-1.89114000	-3.32875300	0.52105000
H	-0.62975600	-3.62568400	2.26019900
H	-2.64643900	-4.10562900	0.62340300
C	1.65579300	0.32001300	-0.30595400
C	1.94212800	1.25692500	0.65704200
S	3.05899100	-0.62342100	-0.67978800
C	3.29778500	1.20103000	1.10140600
H	1.20230200	1.96273500	1.03574800
C	4.01618300	0.22413500	0.46831300
H	3.71780100	1.85934900	1.86095800
H	5.06083500	-0.04374000	0.61425400

TS2(b): (ν = 465.21i(cm^{-1}))

0 1

C	-3.87409300	0.52318600	-1.42874400
C	-2.86032200	1.28865700	-2.03508600
C	-1.51613400	0.92949700	-2.05967900
C	-1.12506800	-0.23644600	-1.32423300
C	-2.20701200	-1.11501600	-0.90709500
C	-3.54295100	-0.74416500	-0.94193700
H	-4.90684500	0.86893900	-1.44402400
H	-0.78120800	1.58734700	-2.52789200
H	-4.31746400	-1.47121100	-0.68402800
H	-3.13599300	2.23105400	-2.51623400
C	-1.46087900	-2.32515200	-1.27365400
O	-1.60699700	-3.51677300	-1.17785700
O	-0.40121200	-1.70733100	-1.86582800
P	0.29497900	0.06869400	-0.12658100
C	-0.18898300	1.51188300	0.80927600
C	0.40080000	2.75494400	0.83406600
C	-0.36211600	3.70131900	1.57274400
H	1.34128700	2.98387900	0.33314700
C	-1.51618900	3.16035000	2.07467200
H	-0.06778700	4.73856000	1.72286100
H	-2.27949800	3.65113900	2.67594100
C	0.56835500	-1.25614600	1.04205700
C	0.89348800	-2.56393400	0.74932800
C	1.11837100	-3.33738400	1.91793400
H	0.95216900	-2.94331200	-0.27068400
C	0.96627300	-2.60325100	3.06706000
H	1.37618700	-4.39521300	1.90909500
H	1.07158000	-2.94267600	4.09611500
C	1.79499300	0.43087300	-1.01711900
C	1.96683300	0.25167900	-2.37140200
C	3.29148700	0.55662000	-2.79083800
H	1.17358400	-0.10999500	-3.02647100
C	4.09182800	0.94319100	-1.74831500
H	3.63722500	0.48557300	-3.82062400
H	5.14163900	1.22985800	-1.78080300
S	3.25874800	0.94434500	-0.25081900
S	0.55141100	-0.97654800	2.75057800
S	-1.69740700	1.50871900	1.66071500

In1(a)

0 1

P	-0.53919600	-0.23227200	-1.84377900
C	-1.54398800	1.12697200	-1.25766700
C	-1.98401900	2.18779800	-2.01207800
S	-2.00672400	1.28333200	0.39901800
C	-2.70177800	3.14017400	-1.23539300
H	-1.80393100	2.26450000	-3.08489400
C	-2.78637300	2.77721500	0.08342700
H	-3.14299100	4.05045600	-1.63822700
H	-3.28371600	3.30848200	0.89306500
C	-1.02642900	-1.70428900	-0.95537400
C	-0.41925400	-2.15327400	0.19834100
S	-2.40557800	-2.65976100	-1.37270300
C	-1.06897400	-3.29260500	0.74595600
H	0.46387100	-1.68299300	0.63244600
C	-2.15640600	-3.66377800	-0.00092400
H	-0.73983900	-3.81062800	1.64549400
H	-2.82713800	-4.50272700	0.18014100
C	1.13424800	0.14280900	-1.31305200
C	1.56784700	1.29207400	-0.69336300
S	2.44780000	-0.93561400	-1.63376500
C	2.97413000	1.29358100	-0.47263000
H	0.89824300	2.10354700	-0.40677500
C	3.57310400	0.15101500	-0.93235300
H	3.51590200	2.10629900	0.00844000
H	4.62812300	-0.11457600	-0.89357800
C	-1.23671200	-0.64329500	-6.25020600
C	0.00579200	-0.11894000	-5.86079900
C	0.28243700	0.01233400	-4.50605900
C	-0.71626500	-0.40249000	-3.60652000
C	-1.98376100	-0.92908000	-3.90275200
C	-2.18762000	-1.02872700	-5.30235100
H	-1.45580100	-0.74776100	-7.31690200
H	1.23814700	0.41992700	-4.16385900
H	-3.13539000	-1.42630700	-5.68919500
H	0.74152800	0.18165600	-6.60856700

Product:

0 1

C	-2.58012800	0.15842400	-4.58258000
C	-1.24492600	-0.13716000	-4.86799700
C	-0.27768700	-0.13620400	-3.86144500
C	-0.66167700	0.17060400	-2.55214800
C	-2.00123500	0.44358400	-2.27452500
C	-2.96644800	0.44606500	-3.27474000
H	-3.31983100	0.15360000	-5.38435000
H	0.75401200	-0.38027700	-4.11053400
H	-4.00223100	0.66452600	-3.01079800
H	-0.94884000	-0.37562900	-5.89028400
C	-2.30996600	0.68548900	-0.82484200
O	-3.42663400	0.92459600	-0.41623800
O	-1.23475100	0.59049800	-0.10859600
P	0.44487400	0.16049200	-1.07867200
C	1.07878600	1.61396900	-0.19815300
C	2.40155300	1.83975200	0.12982500
C	2.59672900	3.05845800	0.83114300
H	3.20990300	1.15398000	-0.11867900
C	1.42152200	3.74410000	1.00629600
H	3.56472000	3.41085500	1.18370400
H	1.27961200	4.70755600	1.49358000
C	0.39938200	-1.38454000	-0.14334800
C	-0.32630400	-2.50372500	-0.45608400
C	-0.07133300	-3.58049500	0.44598000
H	-1.01290600	-2.55786200	-1.30211500
C	0.83729400	-3.24656600	1.41245200
H	-0.54613900	-4.55826600	0.37811100
H	1.20555400	-3.86223700	2.23063600
C	2.03002300	-0.21481900	-2.00221400
C	2.61507400	-1.44320100	-2.19462400
C	3.71154400	-1.41446700	-3.10953500
H	2.26747200	-2.35058200	-1.69956500
C	3.93872700	-0.16106300	-3.60640500
H	4.30597000	-2.28670700	-3.37877600
H	4.71076000	0.15633300	-4.30508400
S	2.83711200	0.98802800	-2.95833000
S	1.39405700	-1.62889200	1.24105800
S	0.07591600	2.89792100	0.38372000
